# Breaking the Boundaries of Bayesian Optimization Utilizing Continuous Chemistry Digital Twins

**DOI:** 10.1021/acs.oprd.5c00449

**Published:** 2026-05-21

**Authors:** Bao Tuan Chau, Yuma Miyai, Thomas D Roper

**Affiliations:** 1 Center for Pharmaceutical Engineering and Sciences, 6889 Virginia Commonwealth University, Richmond, Virginia 23284, United States; 2 Department of Chemical Engineering, Massachusetts Institute of Technology, Cambridge, Massachusetts 02139, United States; 3 Department of Chemical and Life Sciences Engineering, 6889 Virginia Commonwealth University, Richmond, Virginia 23284, United States

**Keywords:** Bayesian optimization, design space, digital twins, boundary expansion, algorithm

## Abstract

A novel chemistry optimization methodology has been developed and applied to digital twins for a nucleophilic aromatic substitution reaction and a two-step process to produce an intermediate for the antibiotic ciprofloxacin. Bayesian optimization (BO) has been shown to be a highly effective algorithm for the optimization of a multitude of problems from machine learning to chemistry; however, all current methodologies require expert knowledge about the problem of interest for BO to be efficient. In this work, we propose a data analysis strategy, Breaking-the-Boundaries (BtB), to enhance the strengths of BO while diminishing the requirement for expert knowledge by allowing for the design space to be expanded autonomously. BtB analyzes the data set in between each BO iteration to determine which parameter would require a boundary expansion to move the design space toward the true global optimum. We apply our BtB-BO methodology to digital twins and provide evidence that it obtains optimal conditions that traditional BO can never reach when given a weak initial design space as small as 0.55% of the full design space. BtB-BO maintains the BO optimization rate and finds optimal solutions for complex optimization problems with weak priors in fewer than 30 iterations in all case studies.

## Graphical abstract

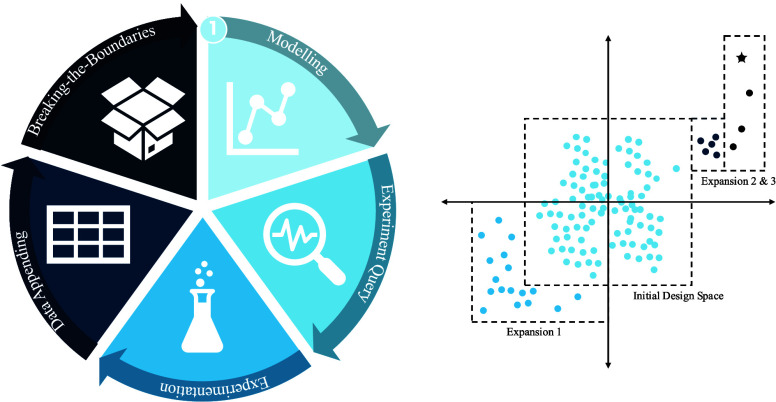

## Introduction

Chemical reaction optimization involving statistical modeling is a rapidly evolving field of research used to understand the underlying relationship between reaction parameters and desired outputs.^1–3^ Chemical reaction design spaces tend to be multidimensional with multiple outputs creating a complex optimization problem that requires experienced chemists’ intuition and multiple iterative procedures.^1,4^ The optimization process utilizes ever higher levels of human and chemical resources as the numbers of reactions, synthetic steps, and process complexity increase.^1^ To counteract the increasing complexity, optimization methods have been developed to leverage statistical modeling and algorithmic experiment optimization.^1,4–8^ A structured method that closely resembles the intuition-based optimization process typically used is called one factor at a time (OFAT) where experiments are conducted in an iterative manner where all but one parameter is kept constant.^1,9,10^ While OFAT is a structured straightforward method, it is inefficient and can lead to false optimum conditions.^9,10^ Design of Experiments (DoE) is the most popular statistical method for optimization in many industries and is extremely prevalent in the pharmaceutical industry.^1,9,11^ DoE allows deeper analysis compared to OFAT and intuition-based optimization processes by studying how the factors interact.^1,9,11^ This application provides for the creation of a statistical model that can predict the underlying function within the explored boundaries. The quantity of experiments required by DoE varies based on the design, with one of the most common being full factorial design, which follows the equation of X^k^ where X is levels and k is parameters.^9,12^ Despite being a robust statistical methodology, DoE’s primary inefficiency comes from its exponential scaling with increased numbers of parameters. As chemistry and pharmaceutical processes become more complex, DoE will ultimately become an inefficient optimization methodology due to the experiment count scaling.

Recent work has shown that Bayesian optimization (BO) is a powerful tool for optimization, particularly for automated optimization platforms.^1,4–8^ BO is an iterative algorithm based on response surface modeling typically utilized in hyperparameter tuning of machine learning models.^4,13^ BO algorithms are derived from Bayesian statistics, which aims to obtain the global maximum within the designated design space utilizing predictions and uncertainty to select iterations to test.^4,13–15^

BO has been the most common method for chemistry self-optimization platforms, which allows for fully autonomous optimization of a designated chemical reaction without human interference.^16–21^ Combining the two techniques allows for increased throughput for chemistry while allowing existing chemists to designate their time elsewhere. Although the optimization campaign occurs autonomously, chemist intuition is required to designate the design space for the BO algorithms to work within similar to DoE.^1,4,13,22,23^ However, weak design space selection becomes detrimental to the process.^23,24^ A large design space is more expensive to explore and optimize reducing the benefits of BO, but a small design space may not contain the global/desired optimum.^25^ Our hypothesis is BO algorithms can utilize autonomous data analysis to discover a global maximum by expanding specific boundaries of a design space reducing the need for chemist intuition.

Shahriari et al. proposed the first two methods for expanding the BO boundaries. The first of two methods is isotropic volume doubling, which drastically increases the design space in set intervals designated by initial Latin hypercube sampling (LHS). The second utilizes regularization of the expected improvement acquisition function to remove the need for a bounded design space. Iterative volume doubling leads BO back toward the large design space issue while the regularization of expected improvement could prevent obtaining a global optimum if the initial design space is far away.^26^

Additional work was conducted by Nguyen et al. that attempts to expand the boundaries of the design space given that the initial design space is in a “weakly specified” design space. The proposed filtering method expands the design space by utilizing the upper and lower confidence bounds and removing low-value sections. This method takes into account the statistics generated by Gaussian processes (surrogate function) of BO and builds upon the first two proposed methods.^24^ Filtering addresses the overexpansion of the design space but prevents the optimization from exploring potentially poor regions by purposefully removing it to save on optimization resources.

Berk et al. developed a method in which they expand and contract the boundaries of the design space utilizing the seen data, Gaussian process modeling, and Thompson sampling to find the global optimum. Their work improved upon the previous filtering method by further reducing the design space while expanding in the desired direction to reduce optimization costs. This method, compared to volume doubling, expands at a reduced rate, which, given a strong initial design space, would benefit optimization cost.^25^ While this method is applicable in material science due to the small incremental adjustments, its strength would decrease in chemistry applications, due to the fact that many raw materials, especially pharmaceutical intermediates, are expensive. Therefore, material consumption and iteration reduction of experiment numbers become paramount. In the event that the global optimum is far from the initial design space, the applicability of methodologies employing incremental changes may be reduced.

This research displays the first examples of Breaking-the-Boundaries (BtB), which utilizes a chemistry-tailored data analysis step before each iteration of traditional BO in a simulated setting with kinetic-based digital twins. The baseline BO algorithm is single-task Bayesian optimization from the Summit python library created to be tailored toward reaction optimization.^27^ In this work, we explore the Breaking-the-Boundaries Bayesian optimization (BtB-BO) methodology utilizing a prebuilt benchmark in Summit for an S_N_Ar reaction of 2,4-difluoronitrobenzene^27,28^ and an in-house kinetic-based digital twin model created on a continuous-flow ciprofloxacin intermediate setup. There are several reports on machine learning-based optimization methods in chemistry and a few on boundary expansion for BO. Established methods utilized strong user-defined bounded design spaces to efficiently use the optimization process. Our methodology removes BO’s reliance on a well-known designated design space with boundary expansion of specific parameters.

## Results and Discussion

### Digital Twin and Breaking-the-Boundary Bayesian Optimization Loop Setup

In this work, digital twins are utilized to complete in silico optimization campaigns for both BtB-BO and traditional BO in flow chemistry. These digital twins are used to simulate physical chemistry and benchmark BtB-BO against BO across different initial design spaces. Both digital twins were modeled by an experimental design space, making extrapolation outside of that space possibly inaccurate.^29^ This experimental design space is labeled as the hard boundaries in BtB-BO, preventing boundary expansion outside that space to ensure that the digital twins are accurate for all simulated experiments. Outside simulated experiments, hard boundaries can be set at parameter limits, such as the minimum or maximum temperature possible in a given system. In the case studies, the digital twins are called to predict the suggested experimental parameters from the BtB-BO or BO.

BtB functions as a data analysis step, before the BO iterations, to evaluate whether design space boundary expansions are required to obtain higher performing parameters. BtB-BO contains multiple coefficients that control the aggressiveness of boundary expansion. The input parameter threshold coefficient (*K*_T,p_) controls the threshold for the input parameter, designating where BtB classifies as near the design space boundaries. The objective threshold coefficient (*K*_T,obj_) controls the threshold for the objective(s), determining the requirements to designate if experiments have converged. The log expansion check (*n*) dictates how many iterations must be completed before suggesting additional boundary expansions. Convergence objective count (*m*) determines how many experiments need to be within the objective threshold before determining that there has been convergence. The expansion coefficient controls how much the boundaries are expanded on the basis of the range of the parameters. The initial screening size represents how many Latin hypercube sampling experiments are used before the optimization campaign begins to mimic screening experiments prior to traditional chemistry optimization. All optimization campaigns have a singular suggested parameter on each iteration after the initial screening set is analyzed. BtB-BO was designed for autonomous optimization campaigns with autonomous checks to halt the optimization campaigns if the goal was achieved, solutions are near objective maximums, or if optimization campaigns have plateaued. For the case studies, these are disabled to fully explore the BO with and without BtB.

### Nucleophilic Aromatic Substitution Digital Twin

The first digital twin we demonstrated our methodology on is a multistep continuous reaction of 2,4-difluoronitrobenzene. This kinetic model was developed by Hone et al. utilizing a continuous-flow platform to fit kinetic parameters.^28^ The reactor setup and controllable parameters are displayed in Scheme 1 and Table 1, respectively.

$$\mathrm{Objective}=\frac{\mathrm{STY}}{1000}-\frac{E-\mathrm{factor}}{100}$$ 1

**Scheme 1:**
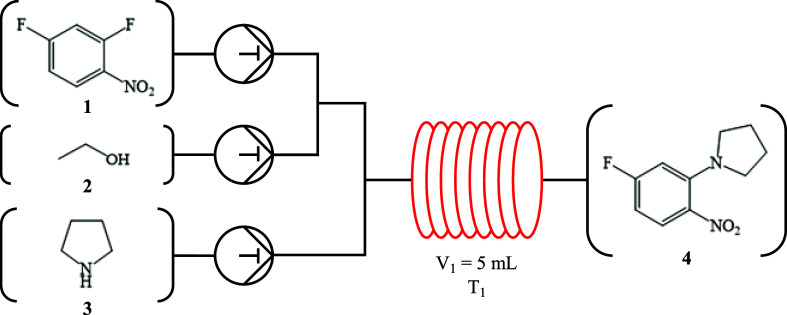
Schematic of a Multistep Continuous Reaction of 2,4-Difluoronitrobenzene (**1**) with Pyrrolidine (**3**) in Ethanol (**2**) to Form the Desired Ortho-Substituted Product (**4**)^28^ ^a^

a (Reproduced/Adapted with permission from ref 28. Copyright 2017 React. Chem. Eng.)

**Table 1:** Summary of the Minimum and Maximum Values for the Boundaries of the Design Space^27^

parameter	lower boundary	upper boundary
residence time (min)	0.5	2.0
pyrrolidine (equiv)	1.0	5.0
2,4-difluoronitrobenzene (mg/mL)	0.1	0.5
temperature (°C)	30	120
space-time yield (kg/m^3^h)	0	13000
environmental factor	0	100

Space-time yield and environmental factor (*E*-factor) are the two objectives that are being optimized and are combined into an objective function (eq 1) to be utilized in the optimization campaign.

We used an initial screening size of 10 experiments, an input parameter threshold coefficient (*K*_T,p_) of 0.25, an objective threshold coefficient (*K*_T,obj_) of 0.04, a log expansion check (*n*) of 5, a convergence objective count (*m*) of 3, a goal of 0.75, an expansion coefficient of 1, and a single suggested parameter set as our parameters for BtB-BO. To see the full optimization campaign of BtB-BO, autonomous optimization checks are disabled, which prevents halting if BtB-BO exceeds the goal, is near maximum potential, or is plateauing. For each run of the BtB-BO experiments and the equivalent BO baseline, the random seed is held constant for the initial screening sample created by the LHS. Each experimental starting condition is displayed in Table 2.

**Table 2:** Summary of Initial Boundary Ranges of Experiments

	initial boundary range			
case study	residence time (min)	pyrrolidine (equiv)	2,4-difluoronitrobenzene (mg/mL)	temperature (°C)
1	0.5–2.0	1.0–5.0	0.1–0.3	30–120
2	1.25–2.0	1.0–5.0	0.1–0.5	30–120
3	1.25–2.0	1.0–5.0	0.1–0.3	30–120

Three specific case studies are displayed in Table 2, where each of the initial design spaces is created to hinder the maximum possible objective. In each case study, the amount the design space can expand is limited by the design space of the digital twin (Table 1) based on the assumption that the digital twin cannot extrapolate outside the designated design space for which it was developed. A traditional BO benchmark is run with the same initial screening samples and design space as a comparison to BtB-BO. Five repeats are conducted with BtB-BO with each keeping every starting condition constant. The results are highlighted in Figure 1 for each case study.

**Figure 1:**
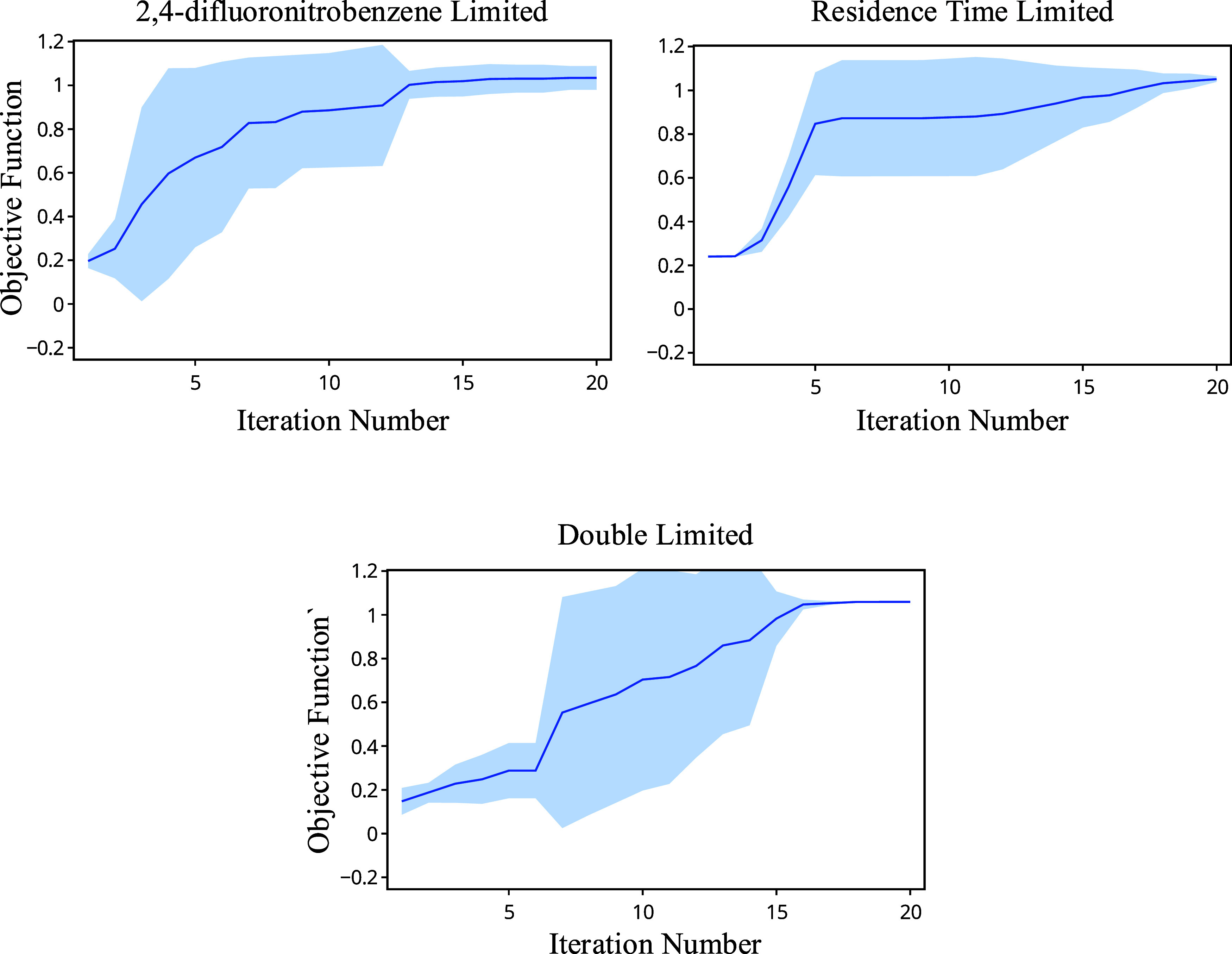
Comparison of the performance of BO with BtB-BO on all three case studies. The average max-hold transform with a 95% confidence interval over five repeats is displayed. The label above each plot refers to the parameter that is limited from the full design space. Initial screening samples are different for each case study.

In the 2,4-difluoronitrobenzene limited and residence time-limited case studies (1 and 2), a singular expansion of their limited parameter is required to fully restore the original design space. In both case studies, the BtB-BO algorithm was able to expand the parameters in the first half of the optimization campaign. After the discovery of the full design space, traditional BO occurs, discovers the maximum, and exceeds the established goal (0.75) with minimal additional iterations.

In the double-limited (3) case study, an expansion for both the 2,4-difluoronitrobenzene and residence time is required to fully restore the design space. In these trials, 2,4-difluoronitrobenzene was instantly expanded after the screening experiments, resulting in the full range for the parameter. As shown in Figure 2, the second expansion (residence time) location varied from iteration 6 to 14. This suggests that the stochastic nature of BO could lead to large variations in expansion locations if there is no parameter convergence, which is required for BtB to consider boundary expansion. Regardless of the expansion location being early or late into the optimization campaign, BtB-BO was able to surpass the established goal and obtain a global maximum comparable to the single limited parameter case studies.

**Figure 2:**
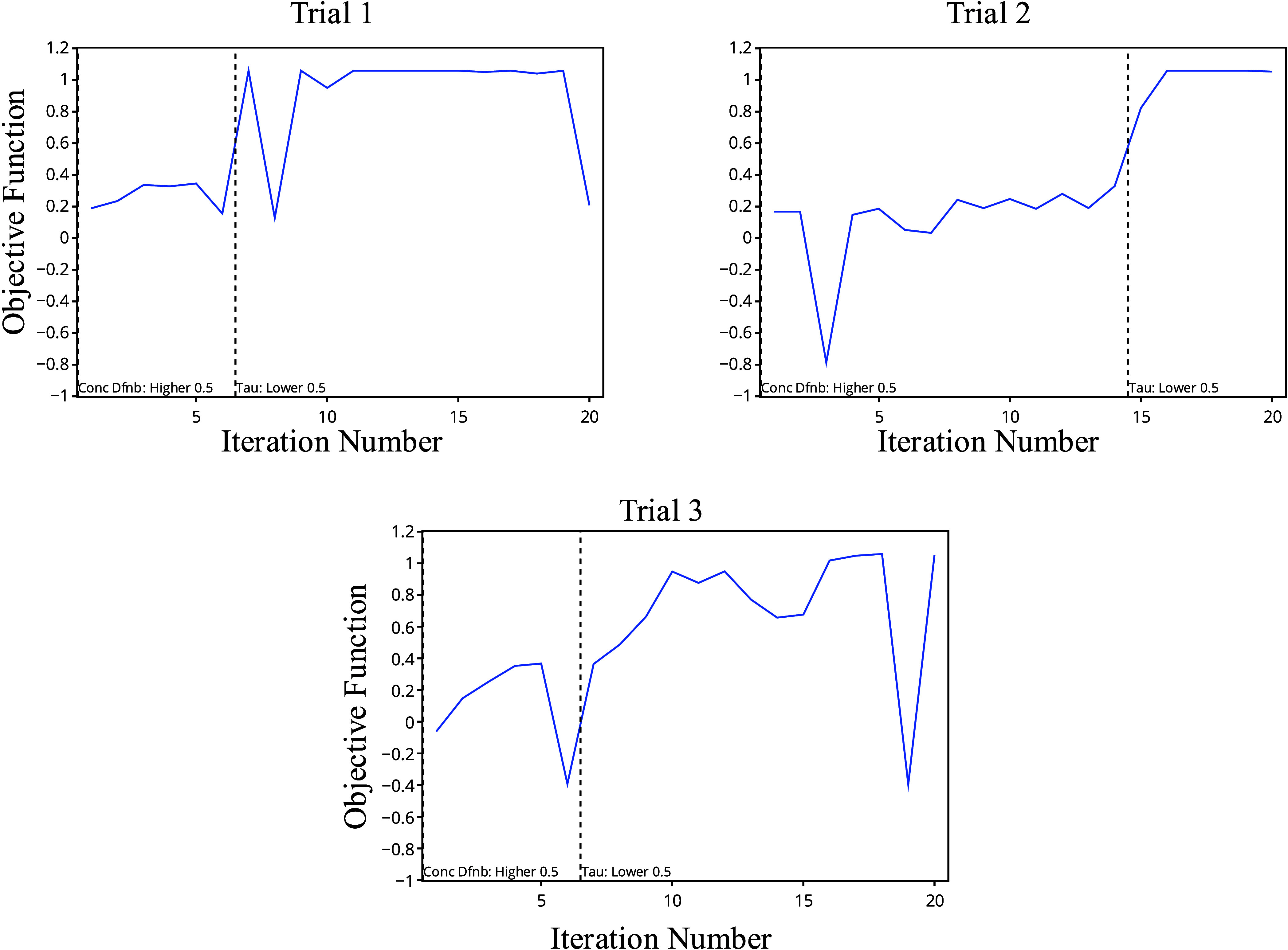
Comparison of the first three trials of the double limited optimization campaign. Vertical dotted lines indicate the boundary expansion locations, labeled with which specific boundaries are expanded, the direction, and the new value.

When utilizing the BtB workflow, BO can significantly exceed traditional performance because of the ability to dynamically update the design space. The expansion of the design space leads to further exploration of the digital twin, resulting in finding a global optimum or true optimum. These case studies on the digital twin suggest that BtB-BO is beneficial in scenarios where boundary expansion is required while also being harmless to performance when not required. Compared to BO, BtB-BO can access a design space that is traditionally unavailable because it has not been previously explicitly stated. With these findings, we were confident that the BtB workflow is effective in simple boundary expansion scenarios. To reinforce the findings with the nucleophilic aromatic substitution digital twin, additional case studies are required to analyze the ability of BtB-BO to complete multiple boundary expansions of individual parameters in combination with multiple parameter expansions in a singular step.

### Ciprofloxacin Intermediate Digital Twin

The second digital twin we demonstrate our methodology on is a two-step continuous synthesis of a ciprofloxacin intermediate. This kinetic model was developed by Miyai et al., utilizing an advection-diffusion-reaction equation to predict the output concentration. The reactor setup and the controllable parameters are displayed in Scheme 2 and Table 3, respectively.

**Scheme 2:**
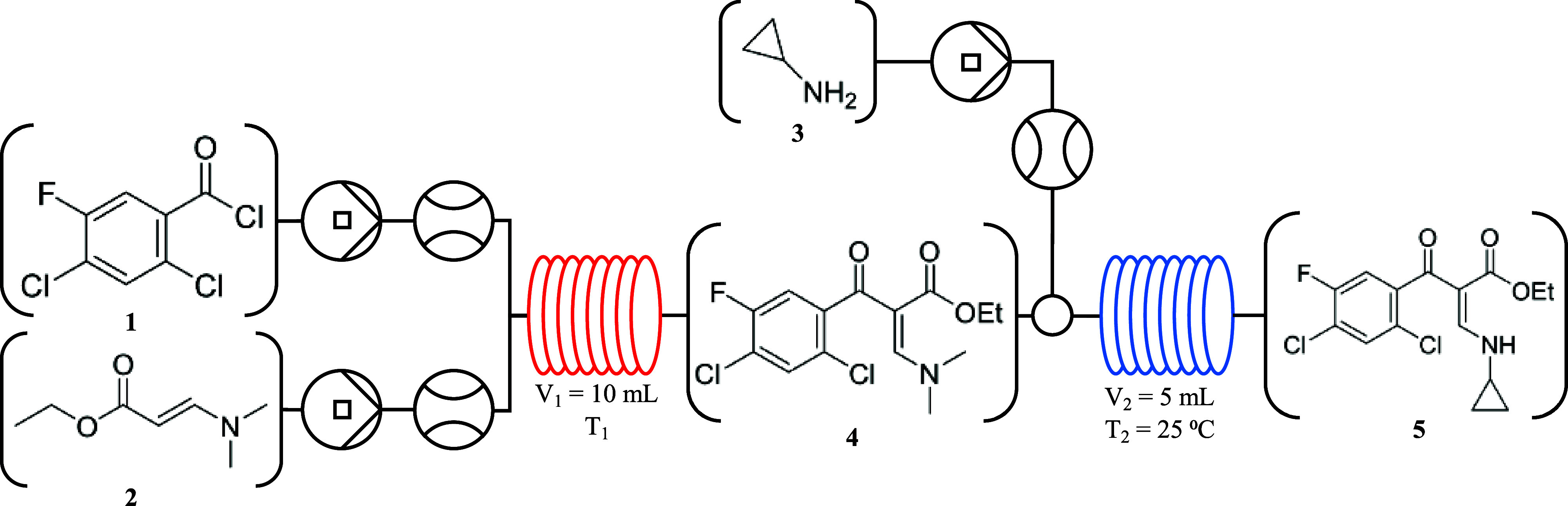
Schematic of the Two-Step Continuous Synthesis of Ciprofloxacin Intermediate (**5**) Containing 2,4-Dichloro-5-fluorobenzoyl Chloride (**1**) Reacting with Ethyl 3-(Dimethylamino)­acrylate (**2**) in Reactor 1 before Further Reacting with Cyclopropylamine (**3**)

**Table 3:** Summary of the Minimum and Maximum Values for the Boundaries of the Design Space

parameter	lower boundary	upper boundary
acrylate flow rate (mL/min)	0.5	5.0
fluoro flow rate (mL/min)	0.5	5.0
cyclo flow rate (mL/min)	0.5	5.0
temperature (°C)	100	200
output concentration (mg/mL)	0	110

The digital twin is utilized in BtB-BO by starting the initial design space in poorly performing locations and comparing the results of the general BO and BtB-BO. The general BO is utilized to visualize the maximum of the initial design space and the full design space. BtB-BO was repeated five times to ensure repeatability robustness. In this case study, we use an initial screening size of 10 experiments created with LHS, an input parameter threshold coefficient (*K*_T,p_) of 0.25, an objective threshold coefficient (*K*_T,obj_) of 0.04, a log expansion check (*n*) of 5, a convergence objective count (*m*) of 3, a goal of 95 mg/mL, an expansion coefficient of 1, and a single suggested parameter set. To see the full optimization campaign of BtB-BO, autonomous optimization checks are disabled, which prevents halting if BtB-BO exceeds the goal, is near maximum potential, or is plateauing. For each run of the BtB-BO experiments and the equivalent BO baselines, the random seed is held constant for the initial screening sample created by LHS, making the screening set identical for the entire case study. Each experimental starting condition is displayed in Table 4.

**Table 4:** Summary of Initial Boundaries of Experiments

	initial boundary range			
case study	acrylate flow rate (mL/min)	fluoro flow rate (mL/min)	cyclo flow rate (mL/min)	temperature (°C)
1	0.5–1.0	2.75–5.0^a^	2.75–5.0^a^	100–200
2	2.75–5.0^a^	0.5–1.0	2.75–5.0^a^	100–200
3	2.75–5.0^a^	2.75–5.0^a^	0.5–1.0	100–200
4^b^	0.5–1.0^a^	4.0–5.0	4.0–5.0	100–200
5^b^	4.0–5.0	0.5–1.0^a^	4.0–5.0	100–200
6^b^	4.0–5.0	4.0–5.0	0.5–1.0^a^	100–200
7	0.5–1.0	4.0–5.0	4.0–5.0	100–200
8	4.0–5.0	0.5–1.0	4.0–5.0	100–200
9	4.0–5.0	4.0–5.0	0.5–1.0	100–200

a Boundaries that were prohibited from expansion.

b Experiments that ran for 30 iterations.

Case studies 1–3 observe the expansion of a singular boundary, 4–6 observe the expansion of two boundaries, and 7–9 observe the expansion of all significant parameters. Significant parameters are parameters that, if hindered, can drastically alter the desired output. The only nonsignificant parameter for the digital twin was determined to be temperature, while all three flow rates influence the output concentration.

Case study 1 contains limited design spaces for the significant parameters where acrylate flow rate was initially set at 0.5–1.0 mL/min and fluoro and cyclo flow rates at 2.75–5.0 mL/min. The parameter ranges are designated in a position to induce poor performance in the output concentration and analyze the performance of BtB-BO, where the only expandable parameter is the acrylate flow rate. Similar initial designs are created for case studies 2 and 3 with fluoro and cyclo, respectively. In all three case studies, it is required to expand the limited parameter at least two times to obtain an output concentration that meets the established goal.

In case study 1, all runs started with an initial screen set that yielded a maximum of 47.81 mg/mL. When BO was run with only the initial design space, it reached a maximum value of 61.69 mg/mL after 20 additional iterations compared to 123.49 mg/mL with the full design space. However, when we utilized the BtB-BO method, the acrylate flow rate was able to expand from 0.5 to 1.0 mL/min to 0.5–4.5 mL/min, which allowed for a new optimal value of 112.61–113.28 mg/mL for the output concentration. All runs of BtB-BO succeeded and surpassed our established goal of 95 mg/mL and reached the optimal value discovered by BO. Each run expanded the acrylate flow rate three times, leading to a final range of 0.5–4.5 mL/min. Plots of the screening and optimization experiment results are displayed in Figure 3.

**Figure 3:**
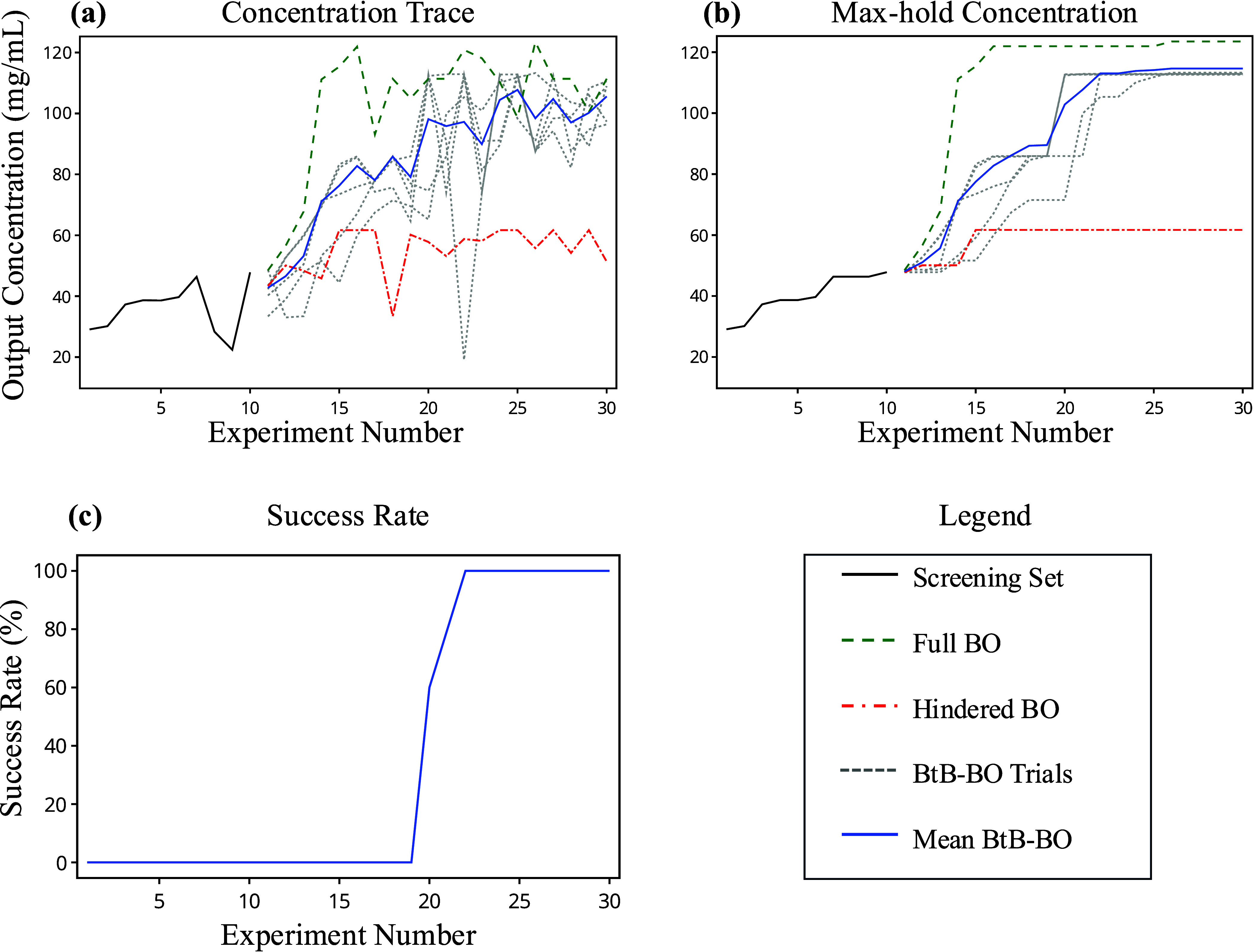
(a) Plot of raw output concentration of case study 1 optimization campaign against experiment. The dotted gray and solid-colored lines indicate the trials and the average data from BtB-BO, respectively. The black dotted-dashed and black dashed lines represent BO with initial design space and full design space, respectively. (b) Max-hold transform of all trials, averaged trials, and baseline comparison. (c) The success rate plot describes the point at which the trials surpass the established goal (95 mg/mL). 100% represents all the trials surpassing the goal.

After obtaining success in each trial before the 20th iteration of the optimization campaign in case study 1, we repeated the experimental processes with case studies 2 and 3 to validate the performance on slightly different initial design spaces. Case study 2 has a screening set with a maximum concentration of 44.16 mg/mL, the hindered design space BO obtained a maximum value of 51.41 mg/mL and the full design space achieved a value of 89.24 mg/mL. After utilizing BtB-BO, we obtain an optimal value of 98.17–103.48 mg/mL. Due to the stochastic nature of Bayesian optimization, two trials converged at an optimum after two boundary expansions reaching a flow rate range of 0.5–2.5 mL/min, while the remaining three converged after three boundary expansions with a range of 0.5–4.5 mL/min. Case study 3 followed a similar trend where the screening set has a maximum value of 48.29 mg/mL, the hinder design space BO reached a maximum value of 52.05 mg/mL, and the full design space obtained a value of 108.26 mg/mL. With BtB-BO, we obtain a maximum value of 104.27–115.20 mg/mL with every trial expanding three times to a final range of 0.5–4.5 mL/min.

In case studies 1–3, BtB-BO outperformed traditional BO when given the same initial design space due to the impossibility of BO to navigate outside the constraints while also reaching the proximity of the peak values observed when utilizing BO with the full design space and the same initial screening set. BtB-BO also succeeded in 100% of the trials by surpassing the established goal within the 20 iterations, proving its robustness and repeatability in repeated expansions.

In addition to multiple expansions on single-parameter case studies, we alternated the parameters that were held constant to form case studies 4–6. This would require BtB-BO to expand the boundaries of two parameters rather than a single parameter multiple times. To ensure that the initial design space provided poor performance, we decreased the ranges of 2.75–5.0 mL/min flow rates to 4.0–5.0 mL/min, which also keeps the requirement for multiple expansions to reach a design space that would yield a high concentration.

Case study 4 had an initial screening set that obtained a maximum value of 38.54 mg/mL, and the hindered design space BO obtained a maximum value of 44.56 mg/mL after 30 iterations compared to the 109.95 mg/mL by the full design space BO. BtB-BO initially ran with 20 iterations and obtained an output range of 76.00–122.11 mg/mL with an 80% success rate. Trial 1 failed to expand the boundaries at a pace that would allow a design space with an optimal solution and time for exploitation to achieve the optimal solution. All other trials had a final output range of 114.04–122.11 mg/mL, which more closely resembles the results seen in case studies 1–3. An additional 10 iterations were run on all trials to see if BtB-BO would converge at a point if given more time. With the additional 10 iterations, a final output concentration range of 114.04–122.11 mg/mL was achieved, resulting in a 100% success rate for the case study. All trials had varying boundary expansion locations with the cyclo and fluoro flow rates with six total expansions occurring during the optimization campaign. Plots for case study 4 are displayed in Figure 4.

**Figure 4:**
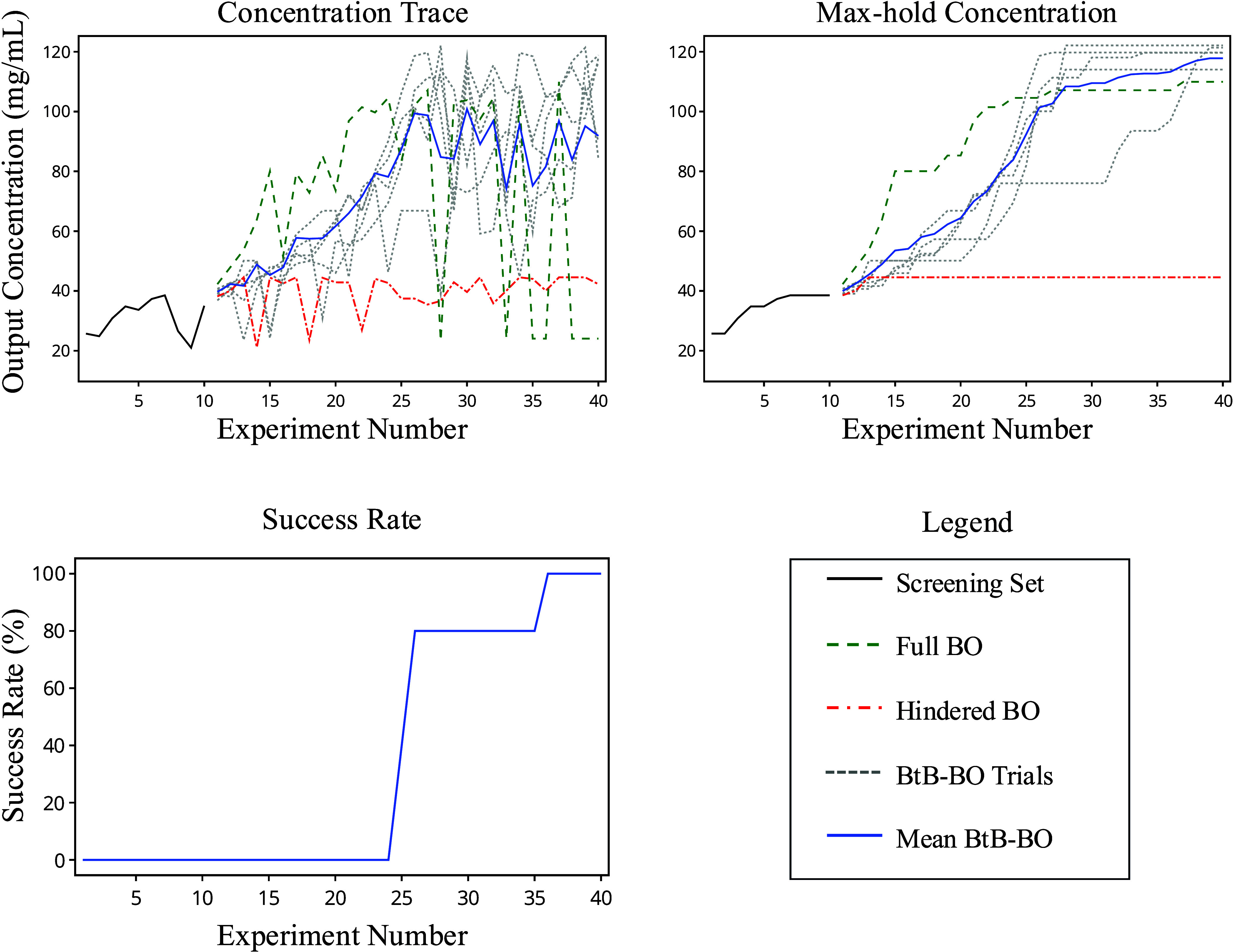
Raw concentration traces and the success rate plots of case study 4. All trials succeeded by experiment number 36 (iteration 26) by surpassing the established goal.

Case studies 5 and 6 were run with 30 iterations rather than the initial 20 iterations to ensure time for BtB-BO to reach the design space with an optimal solution. Case study 5 has a screening set with a maximum concentration of 34.72 mg/mL with hindered BO and full design space BO achieving maximum values of 37.13 and 89.07 mg/mL, respectively. By iteration 20, BtB-BO had a 60% success rate with a range of 70.69–114.36 mg/mL. BtB-BO achieved a 100% success rate by iteration 30 with a range of 110.95–123.96 mg/mL after six total expansions. Case study 6 has a screening set that obtained a maximum value of 41.20 mg/mL with hinder and full design space BO achieving maximum values of 46.40 and 98.38 mg/mL, respectively. In 20 iterations, BtB-BO had a 20% success rate with a range of 70.69–111.71 mg/mL. Pushing to 30 iterations, BtB-BO ended with a 100% success rate with a final optimal value range of 106.13–116.04 mg/mL with four of the trials expanding six total times and one trial expanding five total times.

In case studies 4–6, BtB-BO continued to outperform hindered BO and achieved comparable optimal values as the full design space BO in a starting location that required more boundary expansions than case studies 1–3 for multiple parameters. The increase in the required amount of iteration to achieve a 100% success rate is justifiable due to the increase in the complexity of the boundary expansion and the restriction on the boundary expansion frequency. The total iteration count in cases 4–6 could be reduced if given a smaller log expansion check (*n*) but still relies on the convergence of several data points which depend on the stochastic nature of BO. Case studies 4, 5, and 6 all had various success rates at 20 iterations, but shortly after they obtained 100% success rates at iterations 26, 27, and 24, respectively.

A final set of case studies was attempted to further test the limits of the BtB-BO workflow and algorithm by allowing all significant parameters to expand their boundaries. Case studies 7–9 keep the same initial boundary ranges and screening sets as case studies 4–6 but were only run for 20 iterations. In all previous case studies, the output concentration tends to be higher when the flow rates are near a 1:1:1 ratio. Allowing full access to all significant parameters will test the capabilities of BtB-BO boundary expansion by trying to converge at a middle point to meet the ideal ratio as rapidly as possible.

Case study 7 had an identical screening set as case study 4 that yielded a maximum value of 38.54 mg/mL, and the hindered and full design space BO obtained a maximum of 44.56 and 111.83 mg/mL, respectively. BtB-BO was able to achieve a 100% success rate by iteration 14 and obtain a maximum concentration range of 109.66–116.25 mg/mL by iteration 20. During the optimization campaign, trial 1 had more suggested data points that promoted the expansion of the acrylate flow rate, which caused it to mimic the optimization campaign of case study 1. This caused it to obtain a different optimal point, which led to a similar final design space as the first case study where the optimization campaign was slower to converge. In practical scenarios, this outlier will occur occasionally; however, if we remove it from the success plot, BtB-BO was able to reach a 100% success rate at iteration 9. To compare against full design space BO, we ran 4 additional trials, 20 iterations, and an identical screening set. BO obtained a 100% success rate at iteration 8 with a maximum concentration range of 109.69–116.88 mg/mL. This indicates that the boundary expansion process does not have a large influence on the optimization rate of BO while still obtaining comparable ranges for the final optimal value.

Case studies 8 and 9 were run in the same method as case study 7 while maintaining the same initial screening set and design space as case studies 5 and 6. Case study 8 had an initial screening set with a maximum value of 34.72 mg/mL where the hindered and full design space BO obtained optimal values of 37.11 and 105.74 mg/mL, respectively. BtB-BO obtained a 100% success rate at iteration 14 with an optimal value range of 97.71–104.70 mg/mL at iteration 20. When additional trials of full design space BO were conducted, it failed to reach a 100% success rate in 20 iterations due to trial 2 failing to surpass 95 mg/mL. All other trials succeed by iteration 16 making BtB-BO slightly faster than traditional BO for the optimization of this case study. Case study 9 has an initial screening set with a maximum value of 41.20 mg/mL with hindered and full design space BO obtaining optimal values of 46.40 and 98.00 mg/mL, respectively. BtB-BO obtained a 100% success rate at iteration 14 with an optimal value range of 100.17–114.51 mg/mL by iteration 20. Additional trials were conducted with BO for case study 9, which managed to obtain a 100% success rate at iteration 8 with an optimal value range of 95.20–109.37 mg/mL. For case study 9, BtB-BO was the slower optimization campaign to reach a 100% success rate; however, its optimal value range was higher than BO. The optimal range being higher can be explained by the BtB-BO gradually expanding the design space, which allows it to have an overall smaller space to explore compared to the full design space BO.

Case studies 7–9 display BtB-BO robustness and adaptability when given free rein over all the significant parameters for the digital twin allowing it to reach an optimal value rapidly. In comparison with case studies 4–6, BtB-BO was able to succeed at an earlier iteration number because of the ability to converge at an optimal midpoint where the flow rates are near the ideal 1:1:1 ratio reducing the required iteration count from 30 to 20. When comparing the most similar initial design spaces (case studies 1, 4, and 7) where acrylate was held at a low flow rate at the start, BtB-BO did best when given full access to all significant parameters—this is displayed in Figure 5. Case study 7 was able to have a 100% success rate at iteration 14 (9 if excluding trial 1), which is significantly lower than case study 4, which achieved it at iteration 26 (16 if excluding Trial 1) and comparable to case study 1, which achieved it at iteration 12 in a much simpler initial design space.

**Figure 5:**
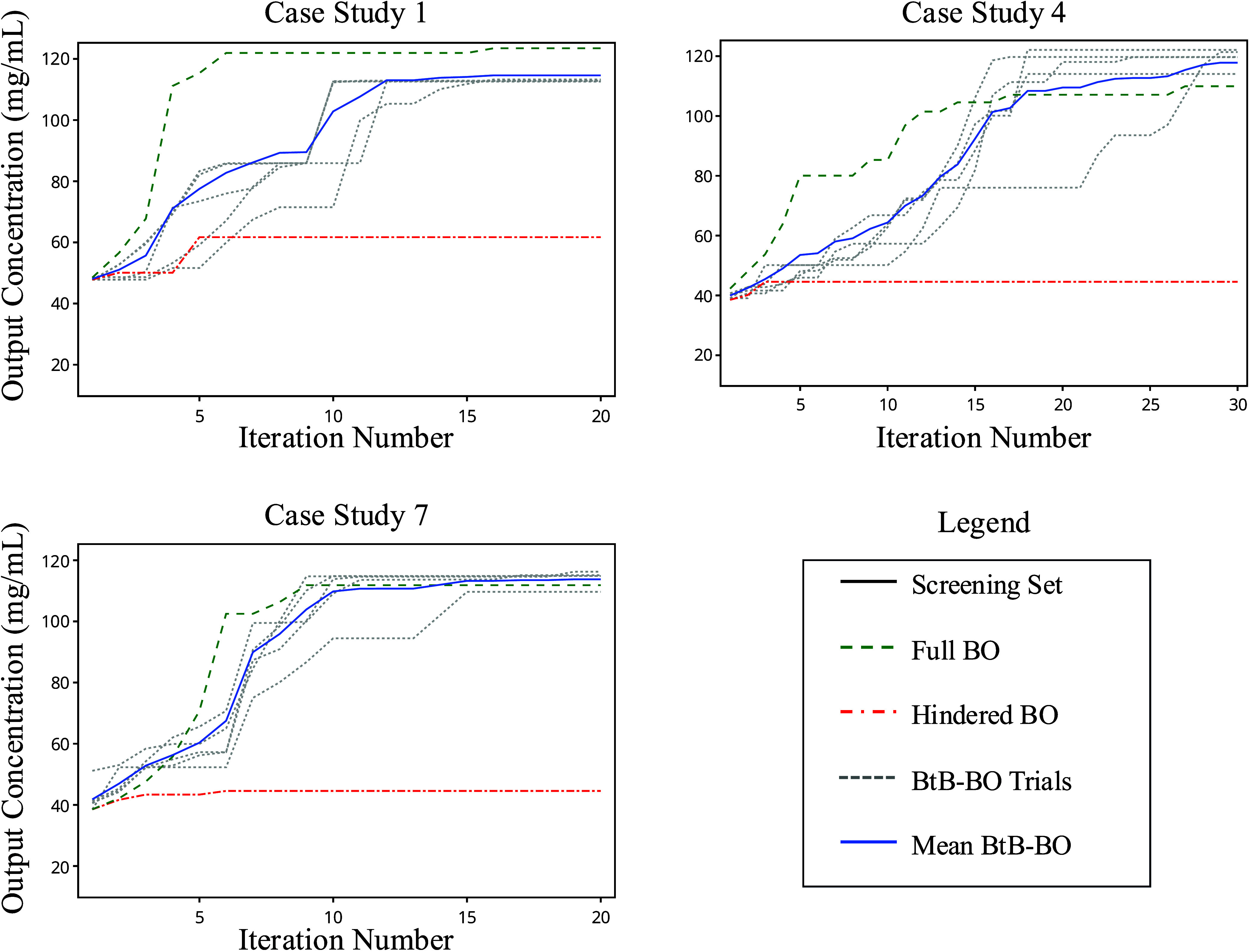
Comparison of the three acrylate limited case studies (1, 4, and 7) optimization campaigns.

Max-hold-transformed plots display the average performance of BtB-BO against BO with the same initial screen and the hindered or full design space.

For each of these case study sets, BtB-BO methodology and workflow were able to achieve comparable results to traditional full design space BO while also altering the design space during the optimization campaign autonomously. The ability to autonomously expand the design space gave BO the ability to exceed the current limitations of poorly defined design spaces, which would hinder optimization campaigns. In the final set of case studies (case studies 7–9), we were able to display that BtB-BO is comparable to full design BO and, in some scenarios, can outperform the optimization rate, success rate, and/or final optimal value. Our methodology achieves optimums that are impossible for traditional BO while retaining the optimization rate in digital twins of chemical reactions.

The proposed BtB methodology has a few limitations. First, BtB can drastically increase the required number of iterations to obtain a successful set of conditions if certain boundaries are prevented from expanding. Second, BtB is under-optimized due to the requirements for convergence being repetitions of similar conditions and does not expand the boundaries more than 100% of the parameter’s range which can slow the optimization campaign. Further development on the workflow can improve the optimization rate which is not noticeable in smaller design spaces displayed in this study but will be seen in larger design spaces that require high iteration amounts to optimization.

## Conclusions

We have developed a new method (BtB) for autonomous optimization involving boundary expansions of the Bayesian optimization (BO) design spaces. This method allows for poorly defined design spaces to have a minimal influence on optimization campaigns and obtain optimal values that traditional optimization methods would not be able to achieve. We describe the workflow and how the data analysis step between each BO iteration functions determine when boundaries should expand their direction and magnitude. The most impactful improvement from current BO approaches is the reduction of the reliance on expert knowledge for a carefully constructed design space. This improvement allows the acquisition function (expected improvement) to find the true global optimum instead of remaining confined to the initially established design space.

The development of the BtB methodology increases the capabilities of self-optimization algorithms by reducing the requirement for domain expertise. Individual parameter boundary expansion prevents the design space from getting too large too quickly, preventing BO from exploring unnecessary locations and further reducing the number of experiments required to reach the final optimal conditions. BtB is not limited to BO algorithms and can be applied to any optimization strategy that requires the design space assignment. This allows for transferability to optimization problems outside of process chemistry to fields such as, but not limited to, materials science, formulation, and machine learning hyperparameter optimization. The reduction in required expertise benefits automated chemistry platforms by increasing autonomy due to the increased flexibility with the design space selection for multiple optimization algorithms.

## Materials and Method

### Computer

All computational work was conducted on an HP Omen laptop equipped with an Intel i7-8750H (2.20 GHz based speed, 6 cores, 12 threads), 16 GB DDR4 (2667 MT/s), and NVIDIA GeForce GTX 1060 (6 GB VRAM).

### Digital Twins

Before testing with costly experiments (e.g., experimental screening, pilot-scale trials, or production-relevant experimentation), we benchmarked and analyzed the capabilities of BO with and without BtB in simulated optimization campaigns utilizing digital twins. The first digital twin we utilized is a benchmark implementation in Summit for a nucleophilic aromatic substitution (S_N_Ar) reaction of difluoronitrobenzene with pyrrolidine.^27,28^ The second is an in-house kinetic model of a multistep continuous manufacturing synthesis platform of a ciprofloxacin intermediate. Both kinetic models display minimal errors with less than 5% error reported within the given operating conditions.^28^ We chose these two digital twins to test our methodology on established kinetic models with common reaction types in medicinal chemistry and pharmaceuticals.

### Bayesian Optimization

All BO processes, baseline and BtB, were performed by utilizing the Summit library (0.8.9)^27^ in Python (3.9). The single-objective Bayesian optimization (SOBO) was the designated baseline function due to it being a wrapper for GPyOpt. The algorithm was run utilizing the default Matern 5.2 kernel, acquisition function of expected improvement, optimizer type of lfbgs, and model type of Gaussian processes. Transforms are applied in the SOBO method for the Hone et al.^28^ digital twin to combine the two output spaces time yield (STY) and environmental factor (E-factor).

### Breaking The Boundaries

The BtB methodology involves boundary conditions and observed data analysis before each iteration of the BO. As shown in Figure 6a, the BtB workflow has multiple primary steps to analyze whether boundary expansion is required for certain parameters.

**Figure 6:**
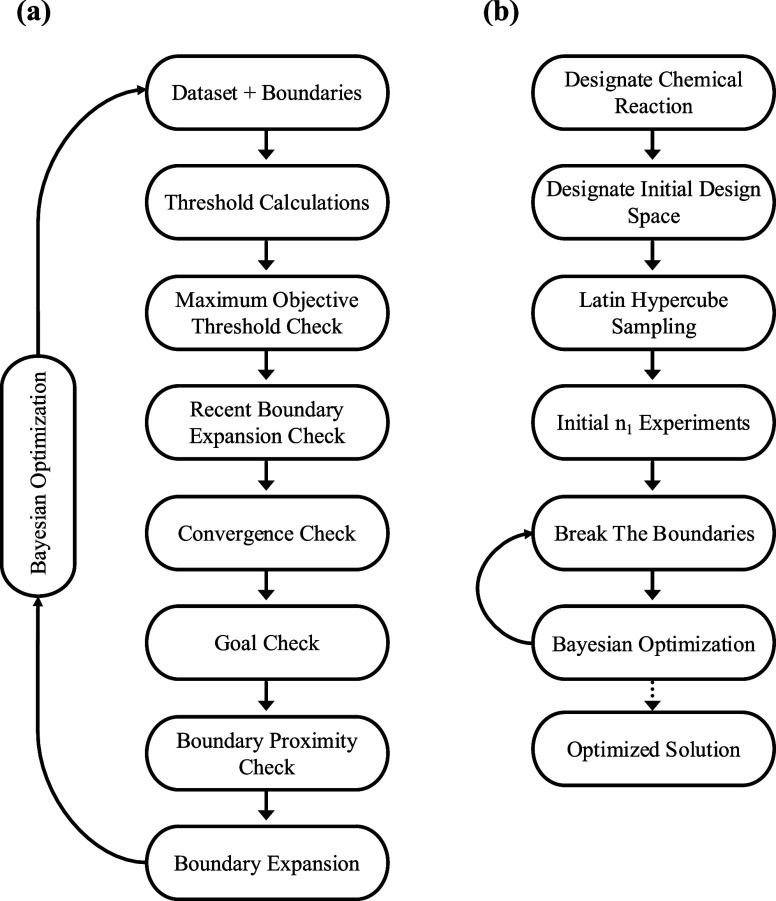
Schematic description of the BtB workflow in the context of chemistry optimization. (a) BtB workflow consists of multiple checks and calculations to determine qualifications for boundary expansion and returns updated boundaries to BO to resume the optimization loop. (b) General workflow for chemistry optimization utilizing BtB workflow with BO.

Beginning every iteration, BtB creates a threshold boundary for the designated objective function (output concentration or STY and *E*-factor) and the current design space boundaries. The threshold for the objective boundary is created utilizing the assumed upper and lower limits of the objective function. The threshold for each parameter is created using the given design space boundaries. Thresholds are calculated to determine the relative distance of observed data points to each other and boundaries:

$$T_{i_{p}}=K_{T,p}\Delta b_{i_{p}}$$ 2

where *T*_*i*_p__ is the threshold at iteration *i* of parameter p, *K*_T,p_ is the threshold coefficient for parameter p, and Δ*b*_*i*_p__ is the difference between the upper and lower boundary values at iteration *i* of parameter p. Once all the boundary thresholds are established, the data proceeds to check the data’s state and the current optimum’s location. The first check is to determine the proximity of the current optimum to the objective function maximum by utilizing the threshold of the objective function:

$$T_{i_{\mathrm{Obj}}}>|y_{i}^{*}-b_{i_{\mathrm{Obj}}}^{U}|$$ 3

where $y_{i}^{*}$ is the current optimum at iteration *i* and $b_{i_{\mathrm{Obj}}}^{U}$ is the upper boundary condition. If the function is true, the optimization process is halted, and it is determined that the optimization campaign discovered the assumed maximum of the objective function. If the function is false, the algorithm looks in past logs to determine whether boundary expansion has occurred in the previous *n* iterations:

$$\sum_{k=\max(0,i-n)}^{i-1}e\mathrm{xpansion}\;(k)\;>\;0$$ 4

where expansion is 1 if there has been an expansion at iteration *k* and 0 if there is none, if the function is true then the remaining steps are bypassed, and optimization resumes. If it is false, then convergence checks for the objective function:

$${\mathrm{y̅}}_{i}^{*}=\frac{1}{m}\overset{m}{\underset{k=1}{\sum }}y_{i,k}^{*}$$ 5

$$\frac{1}{m}\sum_{k=1}^{m}|y_{i,k}^{*}-{\mathrm{y̅}}_{i}^{*}|>T_{i_{\mathrm{Obj}}}$$ 6

where $y_{i,k}^{*}$ is the *k*th observed optimum value and m is the number of objective functions to compare to determine convergence, if the function is true, indicating no convergence, then the remaining steps are bypassed, and optimization resumes. If it is false, it proceeds to the goal check where $y_{i}^{*}$ is compared to an established objective threshold value to determine if the current optimum is sufficient for the optimization goal. If it exceeds the goal, optimization is automatically halted unless the check is disabled. If it does not exceed the goal, it proceeds to the boundary expansion checks to determine the proximity to the boundaries of the design space.

$$\left|x_{i_{p}}^{*}-b_{i_{p}}^{U}\right|\geq T_{i_{p}}$$ 7

$$\left|x_{i_{p}}^{*}-b_{i_{p}}^{L}\right|\geq T_{i_{p}}$$ 8

where $x_{i_{p}}^{*}$ is the input parameter p at iteration *i* that yields the observed optimum. All parameters are analyzed against the current upper and lower boundaries for each parameter to determine the proximity. If eqs 7 and 8 are true, then the parameter is not in proximity to the boundaries, which prohibits boundary expansion. If all parameters result in true functions, then the optimization campaign is halted in automated optimization due to plateauing unless the option is disabled. If any parameter is false for the equations, then it is recorded in a list to be processed in the boundary expansion functions:

$$b_{i+1_{p}}^{U}=b_{i_{p}}^{U}+K_{b}\Delta b_{i_{p}}$$ 9

$$b_{i+1_{p}}^{L}=b_{i_{p}}^{L}-K_{b}\Delta b_{i_{p}}$$ 10

where *K*_b_ is the expansion coefficient. The updated boundary is carried onto the following iteration for the BO. There is an optional check on any hard boundaries set on the optimization campaign, which is to account for known physical limitations of the design space such as a hard upper limit for temperature or pressure to ensure safe operating conditions. The full BO campaign resumes after all the checks are completed or bypassed by certain checks. This allows for all the appropriate parameters in the design space to be expanded to move toward a global optimum regardless of the initial design space if it is within a physically feasible location.

## Supplementary Material



## Data Availability

All data generated in this work is available in Supporting Information. All code utilized in this work is available on GitHub. https://github.com/Roper-Research-Group/Breaking-the-Boundaries

## Notes

Further details on workflow, code preparation, and individual case study result figures (PDF)
