# Supplementary material for: Breaking the Boundaries of Bayesian Optimization Utilizing Continuous Chemistry Digital Twins
Source: Org Process Res Dev. 2026 May 21;30(6):1483–94. doi: 10.1021/acs.oprd.5c00449 (PMC13288922; doi:10.1021/acs.oprd.5c00449)
Supplement: Supplementary file 1 [file op5c00449_si_001.pdf]

Breaking the Boundaries of Bayesian Optimization Utilizing Continuous Chemistry Digital Twins

Bao Tuan Chau[1], Yuma Miyai[2], Thomas D Roper[1,3]*

1. Center for Pharmaceutical Engineering and Sciences Virginia Commonwealth University, Richmond, VA, 23284

2. Department of Chemical Engineering, Massachusetts Institute of Technology, Cambridge, MA, 02139

3. Department of Chemical and Life Sciences Engineering, Virginia Commonwealth University, Richmond, VA, 23284

*Email: tdroper@vcu.edu

Contents

Simulation structure and code workflow 2

Breaking-the-Boundaries simulation workflow 2

2,4-difuloronitrobenzene digital twin code preparation 2

Ciprofloxacin intermediate digital twin code preparation 2

2,4-difluoronitrobenzene digital twin case study 4

Case study result summary 4

Case study 1 result figures 5

Case study 2 result figures 9

Case study 3 result figures 14

Ciprofloxacin intermediate digital twin case study 18

Case study result summary 18

Case study 1 result figures 21

Case study 2 result figures 25

Case study 3 result figures 29

Case study 4 result figures 33

Case study 5 result figures 38

Case study 6 result figures 42
Case study 7 result figures 46
Case study 8 result figures 50
Case study 9 result figures 54
Safety statement 58

Simulation structure and code workflow

Breaking-the-Boundaries simulation workflow

We utilized five different Python scripts to run our Breaking-the-Boundaries (BtB) method with Bayesian optimization (BO). It consists of the BtB Methodology scripts, Summit_Optimizer and Yuma_Optimizer, the simulation scripts, Summit_Simulation and Yuma_Simulation, and a final runner script, Optimizer_Runner. The workflow of the scripts allows for the automated optimization of the digital twins of the chemical reactions and the outputs of the process.

2,4-difuloronitrobenzene digital twin code preparation

We utilized published external digital twins from Summit[1] to analyze our BTB-BO methodology. In the Summit_Simulation.ipynb, we adapted their SnarBenchmark() function to iterate over all experiments suggested by our BTB-BO methodology to emulate the iterative optimization campaign. The SnarBenchmark() function allows for the extraction of parameter conditions (Tau, Equiv_Pldn, Conc_Dfnb, and Temperature) to run the experiments in silico and append the results to an empty list which replaces the results columns (Sty, E_Factor) in the working dataset. This cycle is run for 21 iterations, where run 1 creates the latin hypercube sampled initial screening set of 10 experiments and the remaining 20 iterations completes the iterative optimization campaign.

Ciprofloxacin intermediate digital twin code preparation

To contrast with the previous digital twin, we utilized internal digital twin of a continuous manufacturing of ciprofloxacin intermediate. In Yuma_Simulation.ipynb, we added minimal changes to the original code, the only changes are to extract the average of the last 10 outputs (Output concentration) of the digital twin once it has reached steady state and add it to the dataset. The input into the digital twin is a constant dataset that contains 1250 rows of the parameter conditions (Acrylate flowrate, Fluoro flowrate, Cyclo flowrate, and Temperature) to simulate 1250 sensor readings. 1250 rows were established to ensure steady state by the end of the simulation for the lowest and highest flowrates in the applicable design space of the digital twin. Optimizer_Runner.ipynb is utilized to automatically run all case studies by stringing together Yuma_Optimizer.ipynb with Yuma_Simulation.ipynb for 21 or 31 iterations depending on the case study. The optimization cycle follows the previous digital twin.

Design space hypervolume calculations

For all design space hypervolume calculations, we assume that the design spaces can be represented as a hyperrectangle/hypercube allowing us to use Equation S1 for our calculations. We take the normalized hypervolume (Equation S2) between the case studies and the original full design space to see the initial starting size.

$$V = \prod_{i-1}^{d} (x_i^{max} - x_i^{min})$$

$$V_{norm} = \prod_{i-1}^{d} (\frac{x_i^{max} - x_i^{min}}{X_i^{max} - X_i^{min}})$$

Table S1: Initial design space hypervolume for each case study

Case Study	Case Study Range	Percent of Full Space (%)
2,4-difluoronitrobenzene	1-2	50
2,4-difluoronitrobenzene	3	25
Ciprofloxacin Intermediate	1-3	2.8
Ciprofloxacin Intermediate	4-9	0.55

Additional case studies to analyze the influence of BtB parameters

Three additional case studies were completed to analyze the influence of BtB parameters on the optimization campaigns. Case study SI 1 targets the expansion coefficient by reducing the value from 1 or 100% of the range to 0.25 or 25% of the range. The reduction in the expansion coefficient of BtB causes the optimization rate to drastically decrease due to the lower volume of design space being expanded. This requires the SI case study to need 10 additional iterations to reach the same optimal solution as the default parameters for BtB.

Case study SI 2 adjusts the objective score threshold value from 0.04 to 0.16 decreasing the requirement for BtB to determine convergence. This reduction does not make a large influence in this specific case study where we would expect the optimization rate to increase due to the more lax requirement. Case study SI 3 changes the Expected Improvement acquisition function to a common alternative, Probability of Improvement. This change results in minimal differences in optimization rate in this specific case study.

The changing from default parameters of BtB could lead to differences in optimization rate through aggressiveness of boundary expansion or differing optimization methods. In our case studies, the default BtB parameters yielded high performance in optimization rate and boundary expansion, but it might not be the same for every optimization problem.

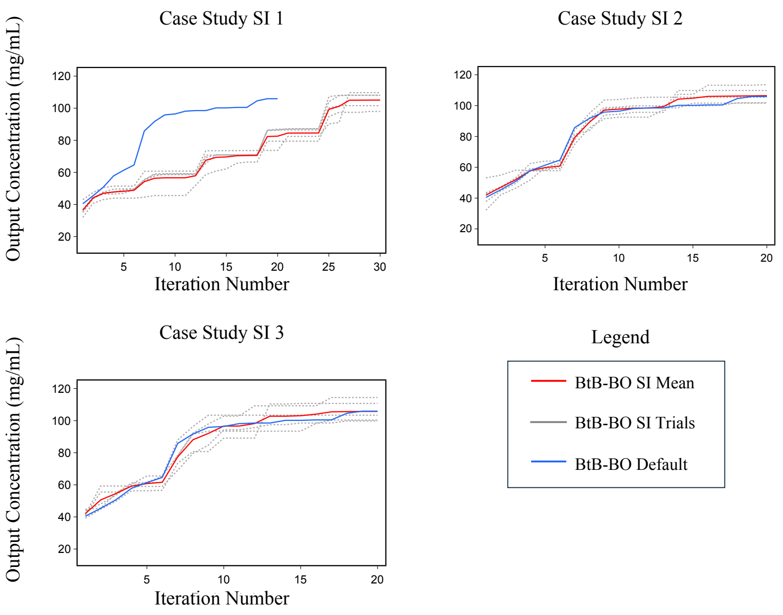

Figure S1: Additional case studies using Ciprofloxacin intermediate case study 9 as the baseline (blue) against varying parameters for BtB-BO (red)

2,4-difluoronitrobenzene digital twin case study

Case study result summary

The results from all case studies for the 2,4-difluoronitrobenzene case studies are presented below with Table S2 displaying each individual trial and the amount of improvement over the screening set and Figures S1-27 displaying all the raw data.

Table S2: Results from individual trials for each 2,4-difluoronitrobenzene digital twin case study

Case Study	Trial Number	Max Screening Value	Max Seen Value
1	1	0.167	0.956
1	2	0.167	1.052
1	3	0.167	1.054
1	4	0.167	1.052
1	5	0.167	1.056
2	1	0.240	1.058
2	2	0.240	1.059
2	3	0.240	1.040
2	4	0.240	1.040
2	5	0.240	1.059
3	1	0.063	1.059
3	2	0.063	1.059
3	3	0.063	1.059
3	4	0.063	1.059
3	5	0.063	1.059

Case study 1 result figures

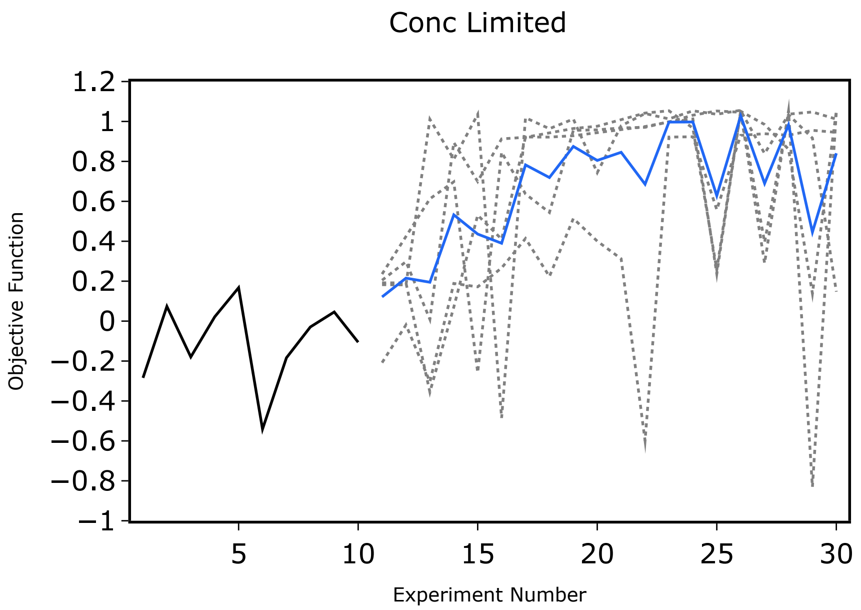

Figure S2: 2,4-difluoronitrobenzene digital twin case study 1 raw dataset plot

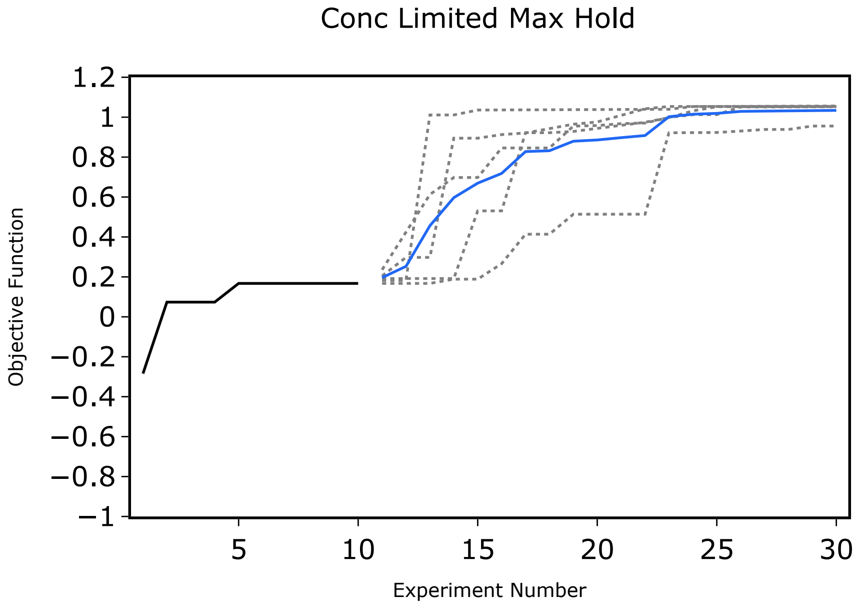

Figure S3: 2,4-difluoronitrobenzene digital twin case study 1 max-hold transform of raw dataset

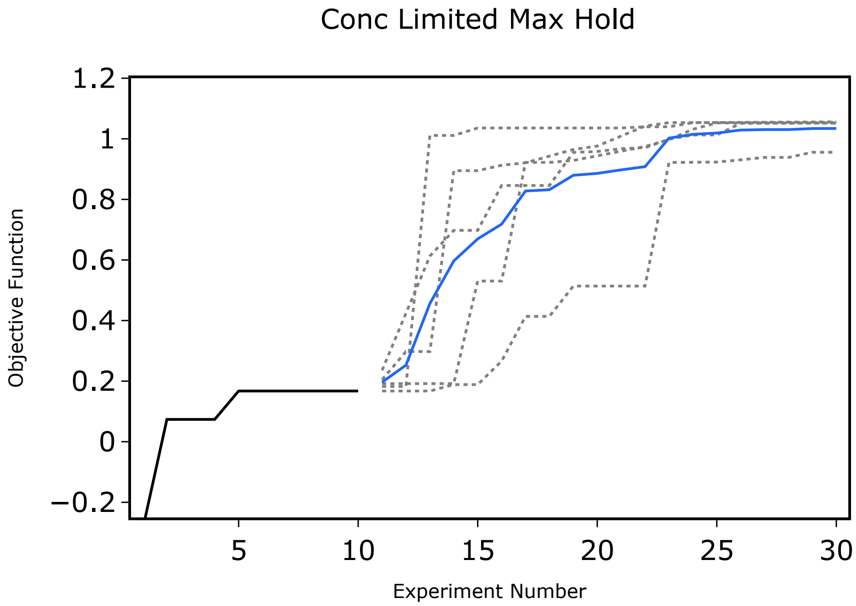

Figure S4: 2,4-difluoronitrobenzene digital twin case study 1 zoomed max-hold transform of raw data

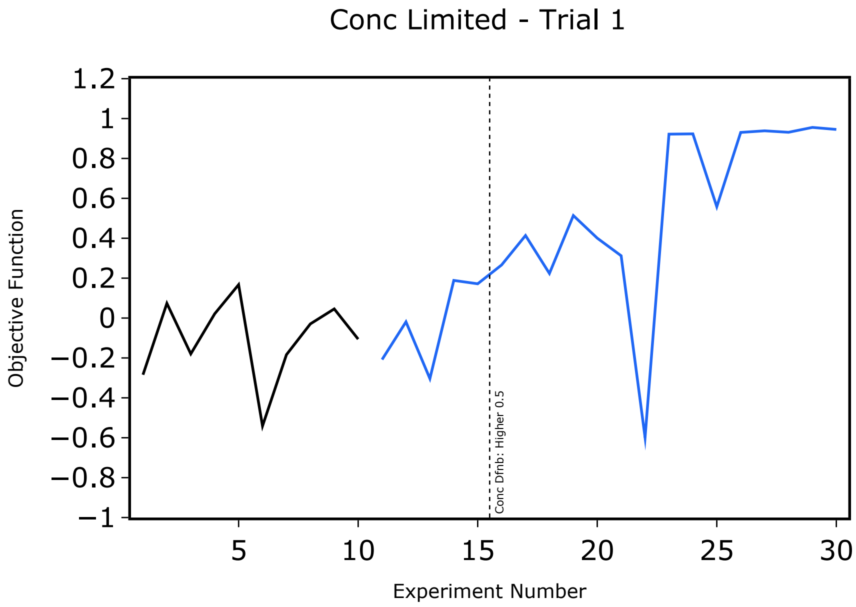

Figure S5: 2,4-difluoronitrobenzene digital twin case study 1 trial 1 raw data with expansion annotation

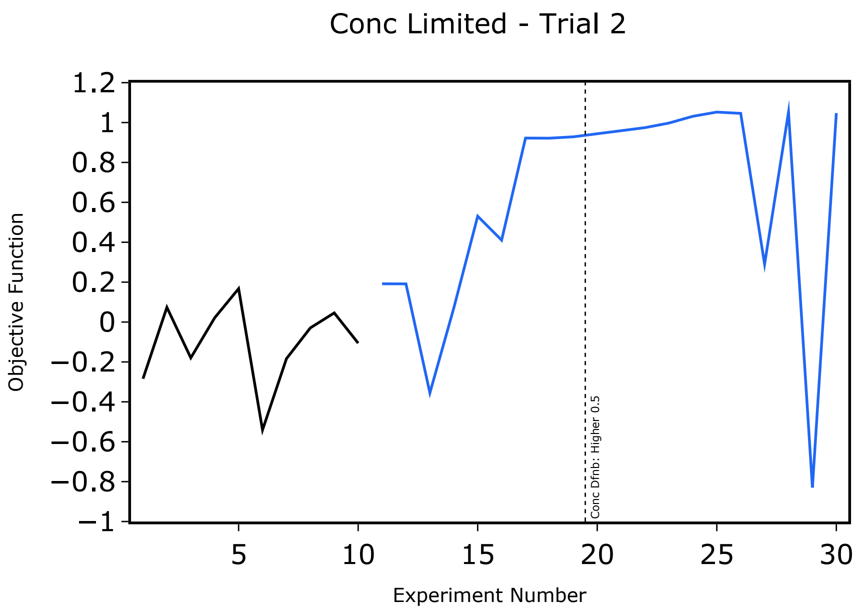

Figure S6: 2,4-difluoronitrobenzene digital twin case study 1 trial 2 raw data with expansion annotation

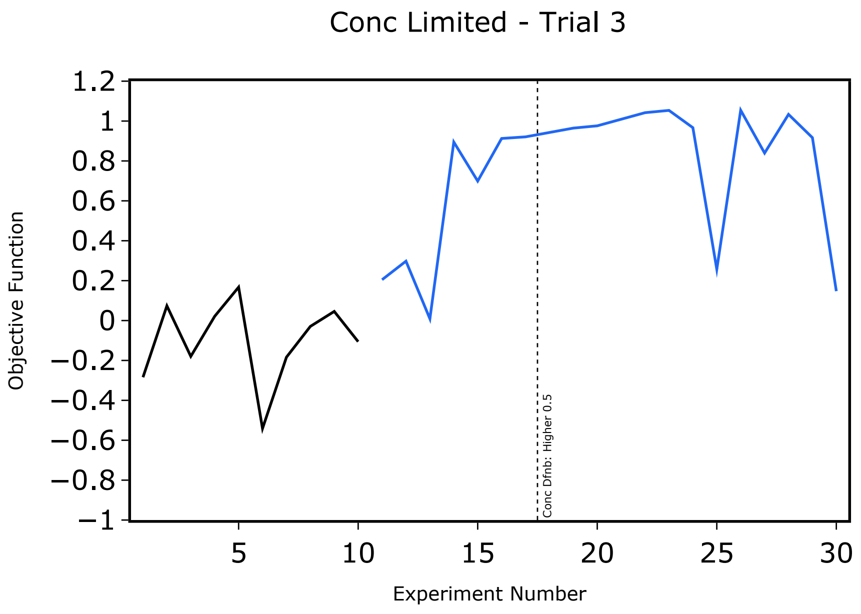

Figure S7: 2,4-difluoronitrobenzene digital twin case study 1 trial 3 raw data with expansion annotation

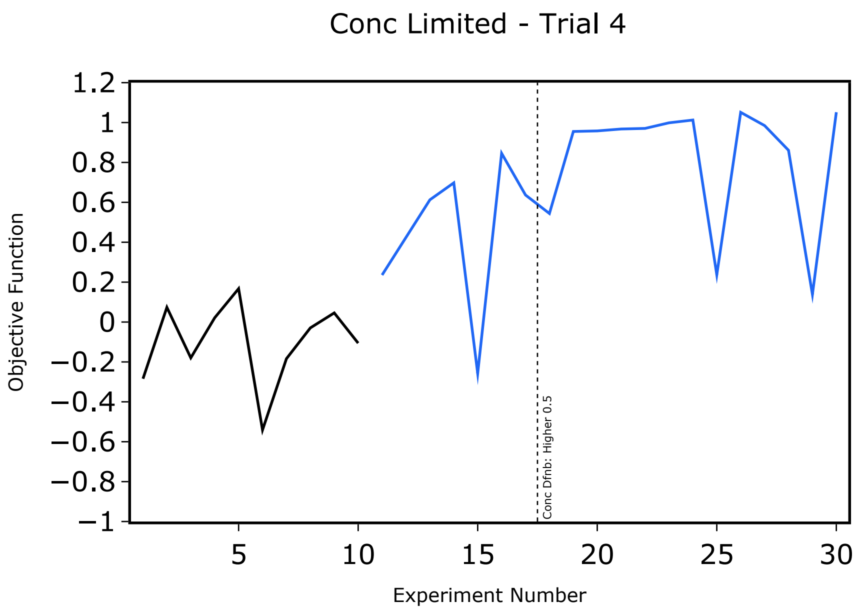

Figure S8: 2,4-difluoronitrobenzene digital twin case study 1 trial 4 raw data with expansion annotation

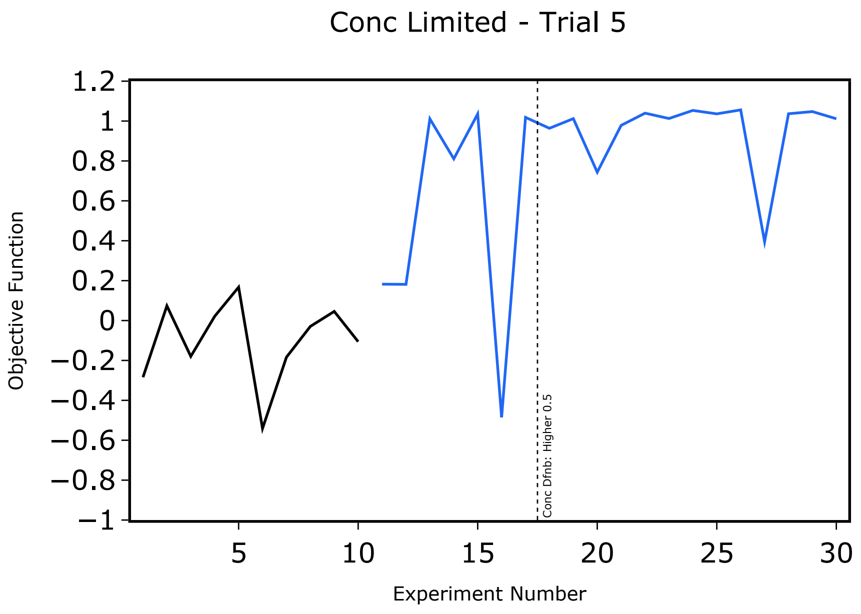

Figure S9: 2,4-difluoronitrobenzene digital twin case study 1 trial 5 raw data with expansion annotation

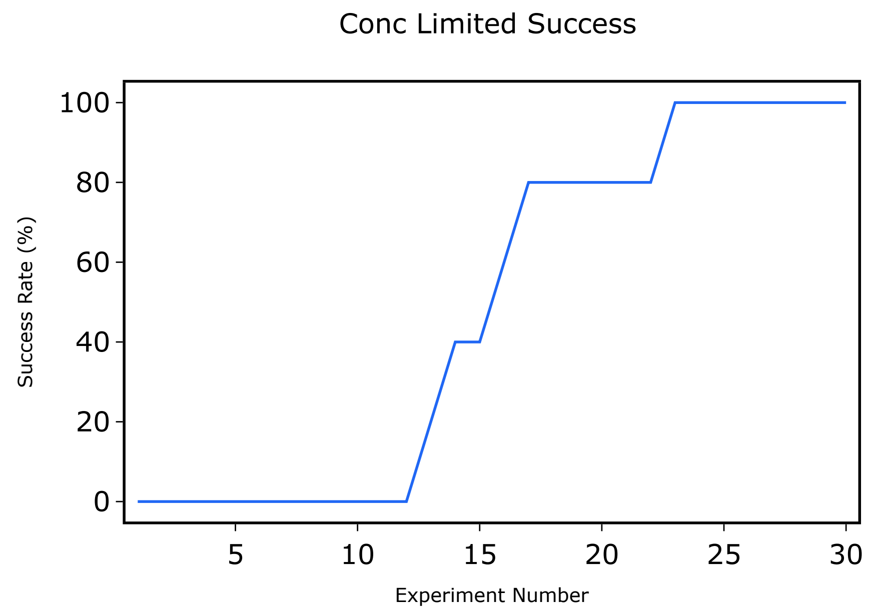

Figure S10: 2,4-difluouronitrobenzene digital twin case study 1 success rate plot

Case study 2 result figures

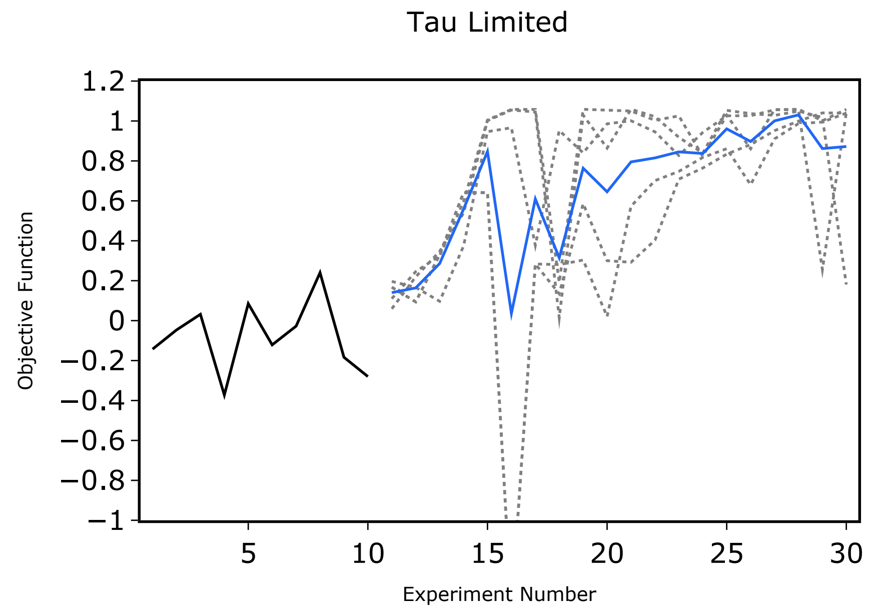

Figure S11: 2,4-difluoronitrobenzene digital twin case study 2 raw data

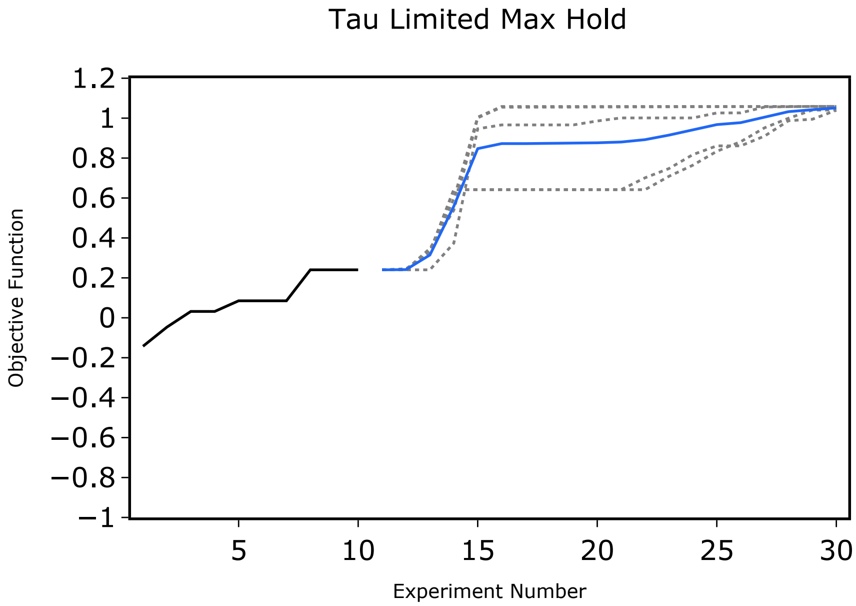

Figure S12: 2,4-difluoronitrobenzene digital twin case study 2 max-hold transform of raw data

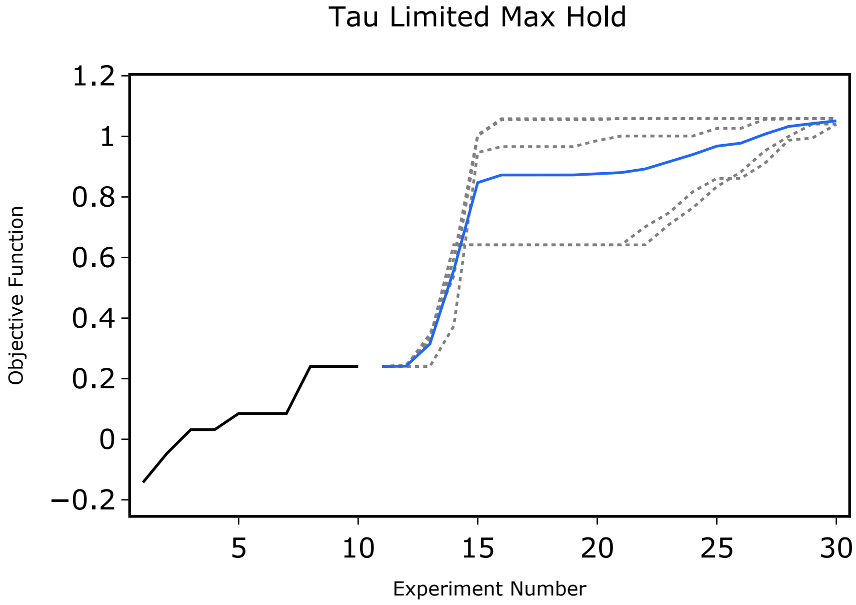

Figure S13: 2,4-difluoronitrobenzene digital twin case study 2 zoomed max-hold transform of raw data

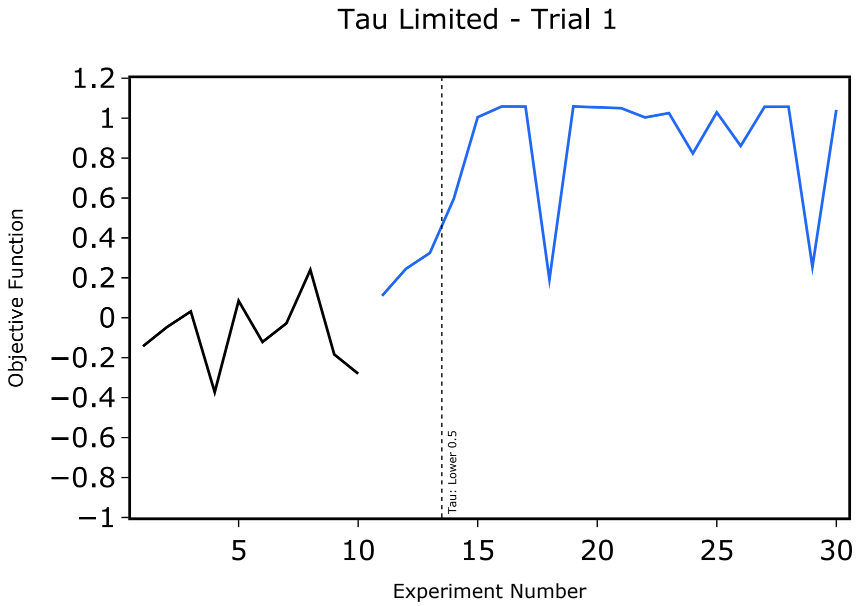

Figure S14: 2,4-difluoronitrobenzene digital twin case study 2 trial 1 raw data with expansion annotation

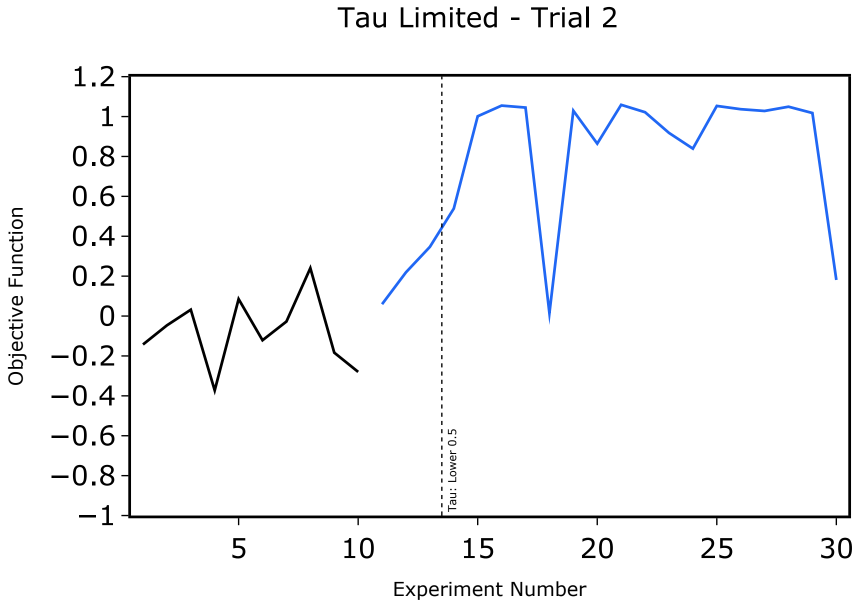

Figure S15: 2,4-difluoronitrobenzene digital twin case study 2 trial 2 raw data with expansion annotation

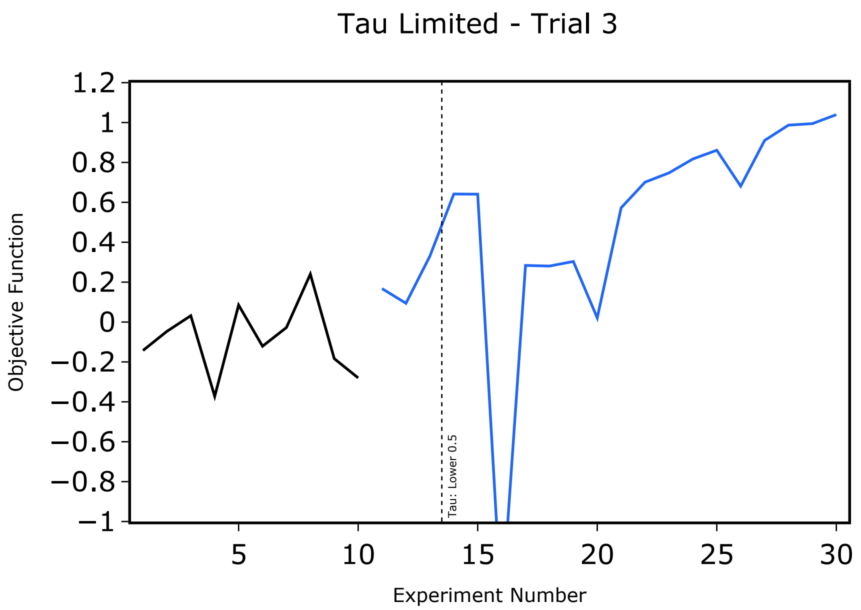

Figure S16: 2,4-difluoronitrobenzene digital twin case study 2 trial 3 raw data with expansion annotation

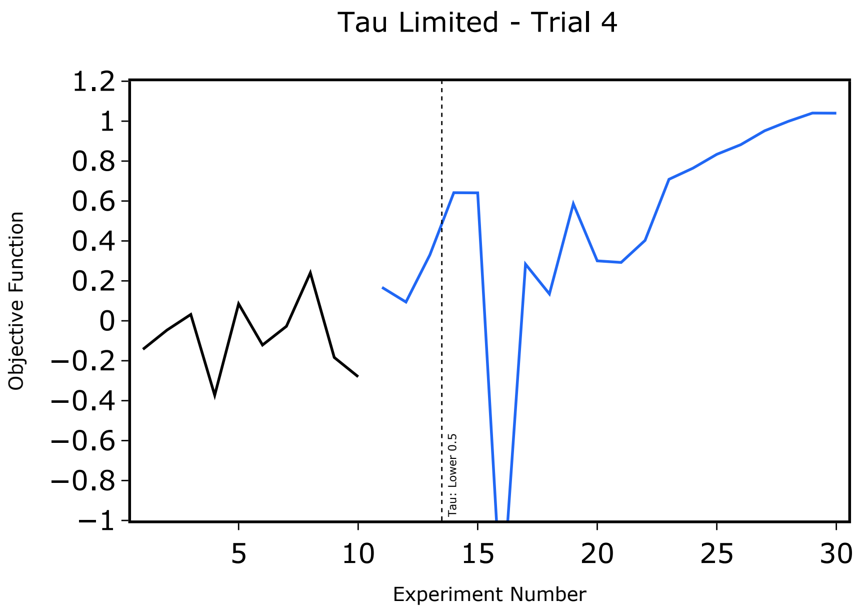

Figure S17: 2,4-difluoronitrobenzene digital twin case study 2 trial 4 raw data with expansion annotation

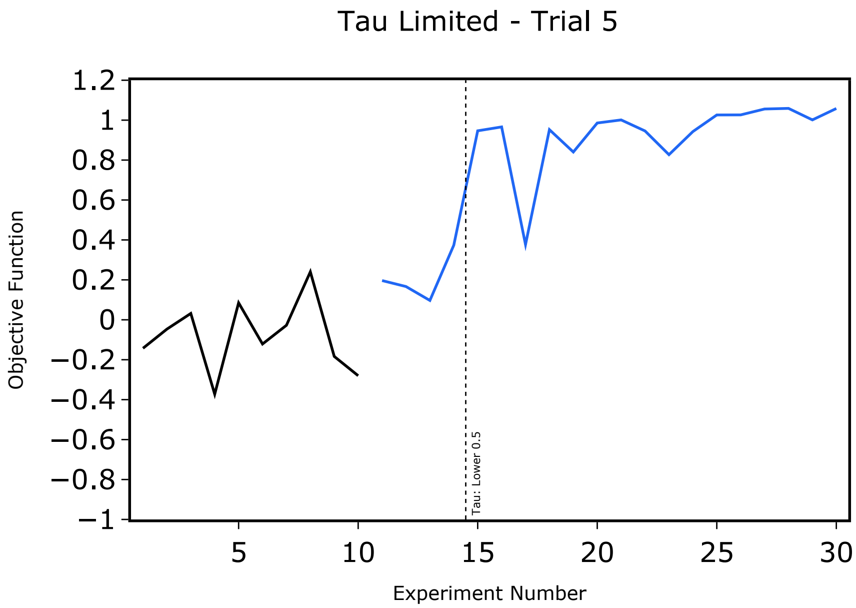

Figure S18: 2,4-difluoronitrobenzene digital twin case study 2 trial 5 raw data with expansion annotation

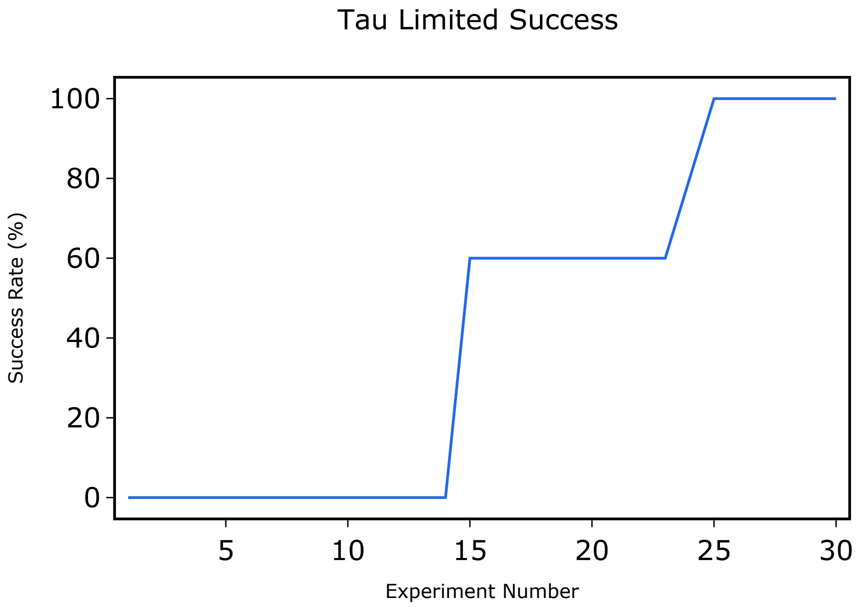

Figure S19: 2,4-difluoronitrobenzene digital twin case study 2 success rate plot

Case study 3 result figures

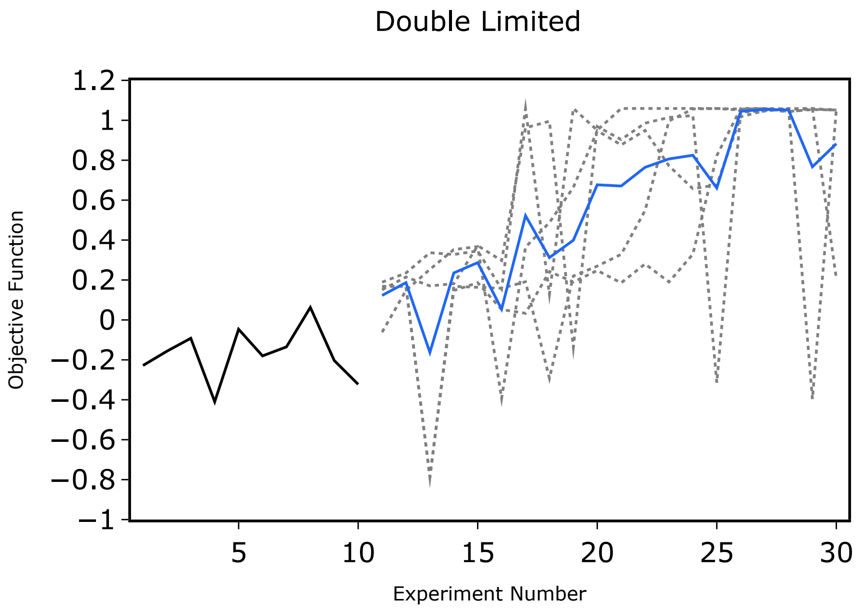

Figure S20: 2,4-difluoronitrobenzene digital twin case study 3 raw data

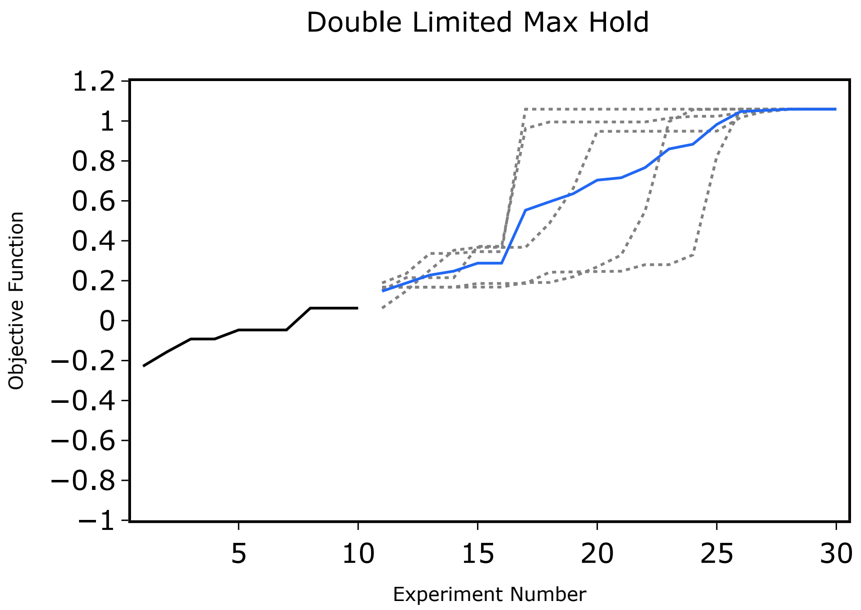

Figure S21: 2,4-difluoronitrobenzene digital twin case study 3 max-hold transform of raw data

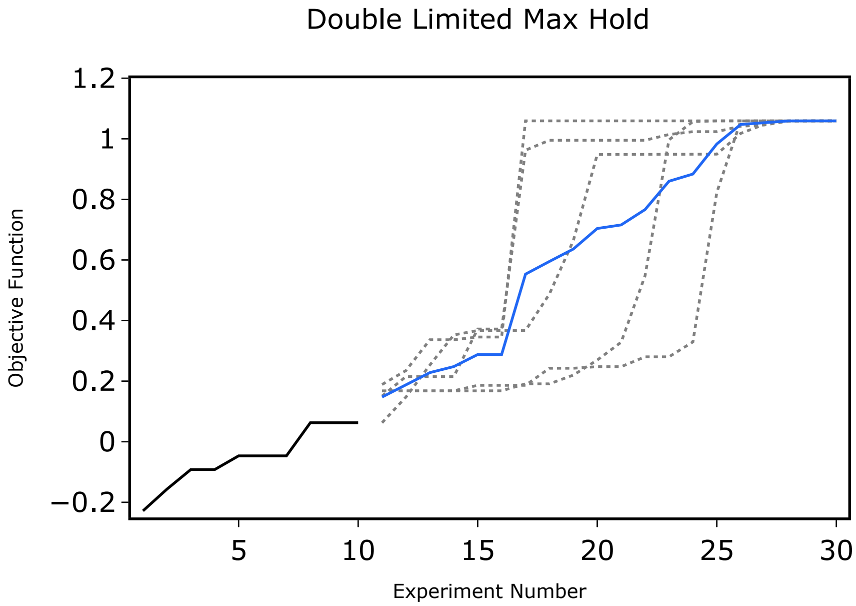

Figure S22: 2,4-difluoronitrobenzene digital twin case study 3 zoomed max-hold transform of raw data

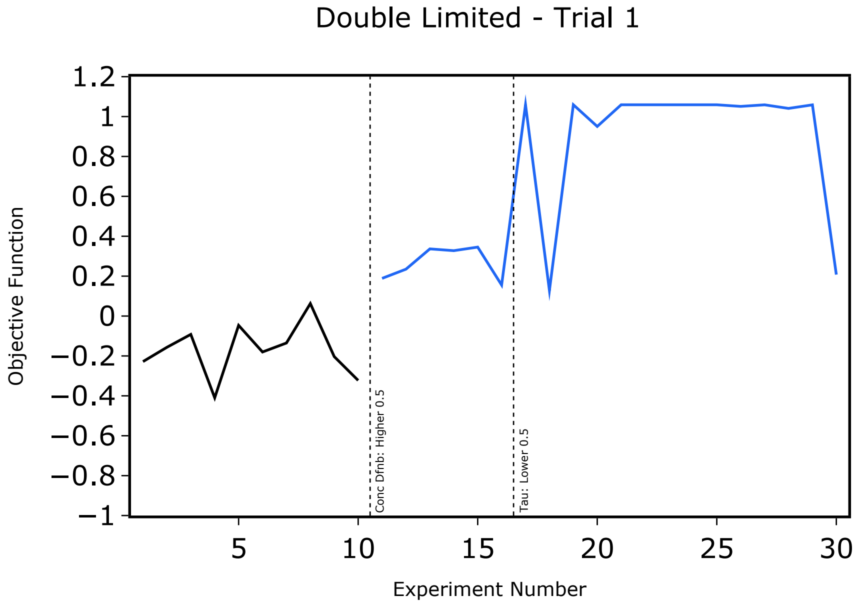

Figure S23: 2,4-difluoronitrobenzene digital twin case study 3 trial 1 raw data with expansion annotation

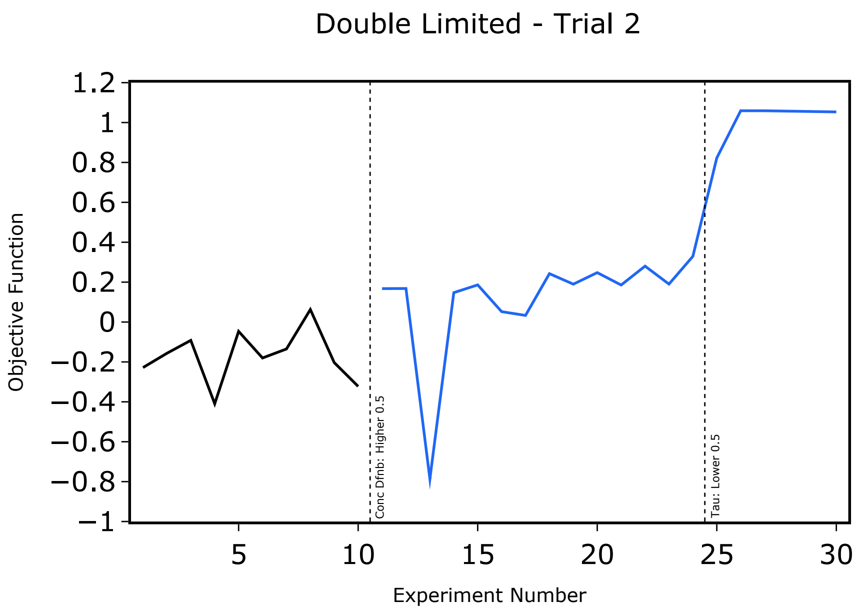

Figure S24: 2,4-difluoronitrobenzene digital twin case study 3 trial 2 raw data with expansion annotation

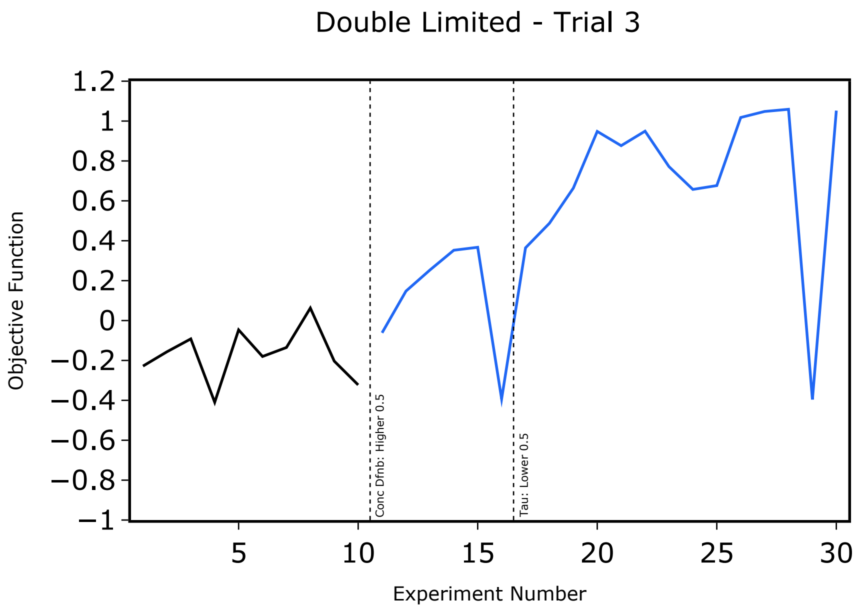

Figure S25: 2,4-difluoronitrobenzene digital twin case study 3 trial 3 raw data with expansion annotation

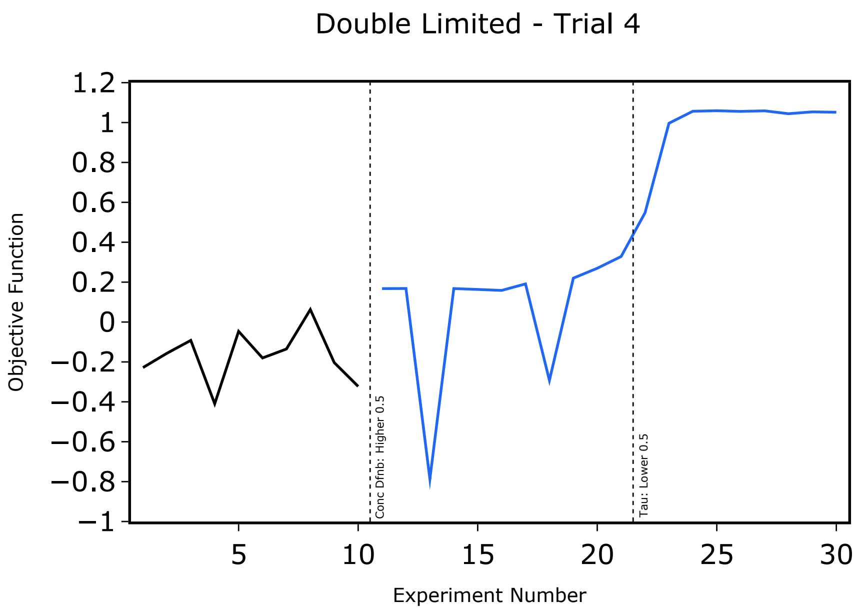

Figure S26: 2,4-difluoronitrobenzene digital twin case study 3 trial 4 raw data with expansion annotation

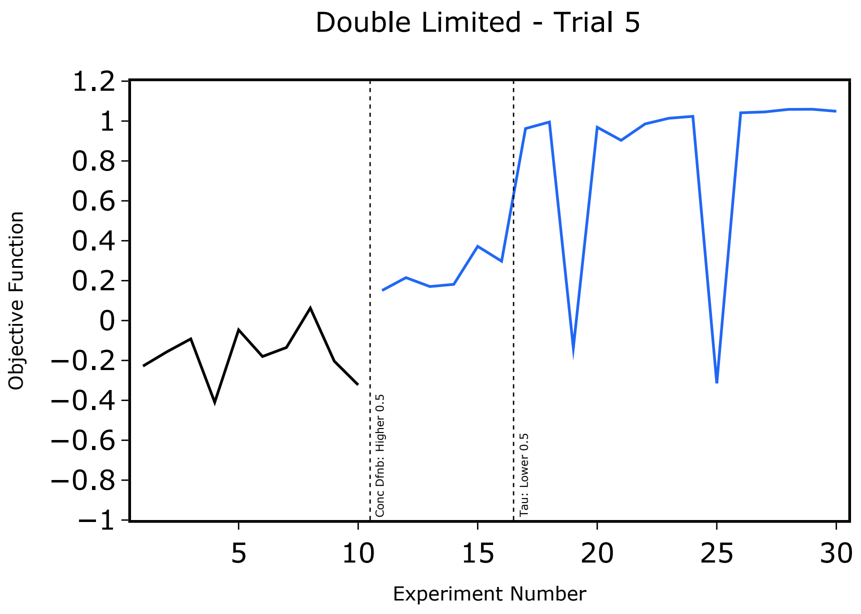

Figure S27: 2,4-difluoronitrobenzene digital twin case study 3 trial 5 raw data with expansion annotation

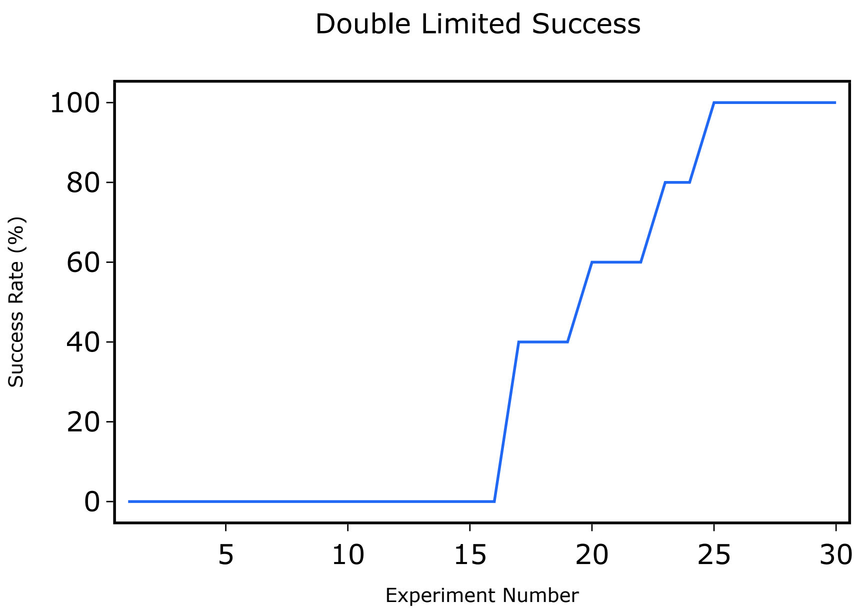

Figure S28: 2,4-difluoronitrobenzene digital twin case study 3 success rate plot

Ciprofloxacin intermediate digital twin case study

Case study result summary

The results from all case studies for the ciprofloxacin case studies are presented below with Table S3 displaying each configuration type, individual trial and the amount of improvement over the screening set and Figures S28-99 displaying all the raw data. The results also include comparisons with traditional BO with the same design space as the screening set and traditional BO with the full design space but with the same screening set to see how the methodology would compare to current standard methods.

Table S3: Results from individual trials for each ciprofloxacin intermediate digital twin case study. "F" indicates a full design space, BTB for Breaking-the-Boundaries configuration, and Hard for BtB disabled.

Case Study	Trial Number	Configuration	Max Screening Value	Max Seen Value
1	1	BTB	47.81	112.89
1	2	BTB	47.81	113.28
1	3	BTB	47.81	112.81
1	4	BTB	47.81	112.66
1	5	BTB	47.81	112.61
1	Baseline	Hard	47.81	61.69
1	F	Hard	47.81	123.49
2	1	BTB	44.16	100.93
2	2	BTB	44.16	103.48

2	3	BTB	44.16	98.21
2	4	BTB	44.16	103.48
2	5	BTB	44.16	98.17
2	Baseline	Hard	44.16	51.41
2	F	Hard	44.16	89.24
3	1	BTB	48.29	115.20
3	2	BTB	48.29	113.55
3	3	BTB	48.29	113.10
3	4	BTB	48.29	104.43
3	5	BTB	48.29	104.27
3	Baseline	Hard	48.29	52.05
3	F	Hard	48.29	108.26
4	1	BTB	38.54	121.37
4	2	BTB	38.54	119.74
4	3	BTB	38.54	122.11
4	4	BTB	38.54	114.04
4	5	BTB	38.54	119.65
4	1S	BTB	38.54	76.00
4	2S	BTB	38.54	119.74
4	3S	BTB	38.54	122.11
4	4S	BTB	38.54	114.04
4	5S	BTB	38.54	118.07
4	Baseline	Hard	38.54	44.56
4	Full	Hard	38.54	109.95
5	1	BTB	34.72	112.39
5	2	BTB	34.72	114.73
5	3	BTB	34.72	115.40
5	4	BTB	34.72	123.96
5	5	BTB	34.72	110.95
5	1S	BTB	34.72	112.51
5	2S	BTB	34.72	72.14
5	3S	BTB	34.72	111.34
5	4S	BTB	34.72	114.36
5	5S	BTB	34.72	70.69
5	Baseline	Hard	34.72	37.13
5	Full	Hard	34.72	89.07
6	1	BTB	41.20	106.13
6	2	BTB	41.20	111.38
6	3	BTB	41.20	111.57
6	4	BTB	41.20	111.71
6	5	BTB	41.20	116.04
6	1S	BTB	41.20	86.31
6	2S	BTB	41.20	80.20
6	3S	BTB	41.20	75.12
6	4S	BTB	41.20	111.71
6	5S	BTB	41.20	87.85

6	Baseline	Hard	41.20	46.40
6	Full	Hard	41.20	98.38
7	1	BTB	38.54	109.66
7	2	BTB	38.54	114.77
7	3	BTB	38.54	115.11
7	4	BTB	38.54	114.85
7	5	BTB	38.54	116.25
7	1F	Hard	38.54	111.83
7	2F	Hard	38.54	116.88
7	3F	Hard	38.54	112.53
7	4F	Hard	38.54	109.69
7	5F	Hard	38.54	110.98
7	Baseline	Hard	38.54	44.56
8	1	BTB	34.72	98.31
8	2	BTB	34.72	103.65
8	3	BTB	34.72	97.71
8	4	BTB	34.72	104.70
8	5	BTB	34.72	98.73
8	1F	Hard	34.72	105.74
8	2F	Hard	34.72	84.44
8	3F	Hard	34.72	102.28
8	4F	Hard	34.72	103.05
8	5F	Hard	34.72	107.99
8	Baseline	Hard	34.72	37.11
9	1	BTB	41.20	114.13
9	2	BTB	41.20	108.37
9	3	BTB	41.20	100.17
9	4	BTB	41.20	100.28
9	5	BTB	41.20	114.51
9	1F	Hard	41.20	98.00
9	2F	Hard	41.20	95.20
9	3F	Hard	41.20	97.76
9	4F	Hard	41.20	108.47
9	5F	Hard	41.20	109.37
9	Baseline	Hard	41.20	46.40

Case study 1 result figures

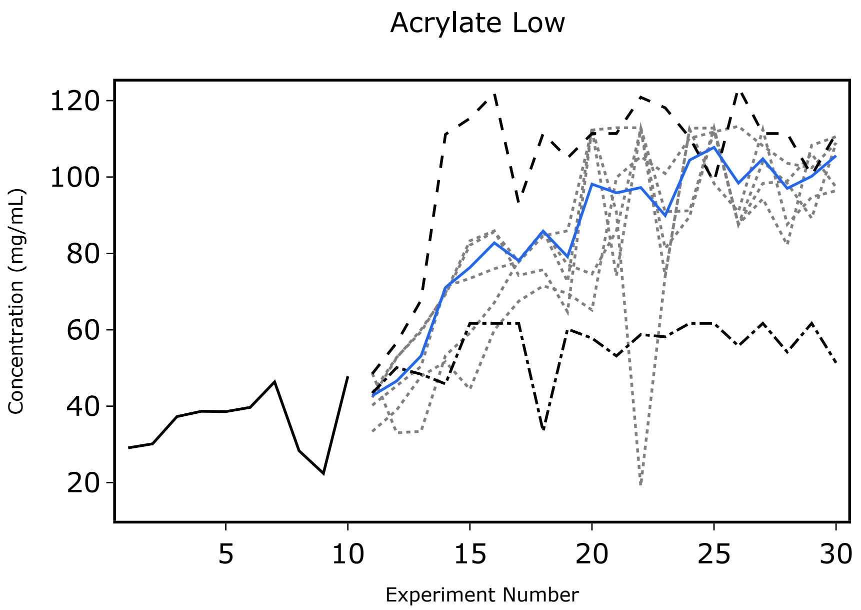

Figure S29: Ciprofloxacin intermediate digital twin case study 1 raw data

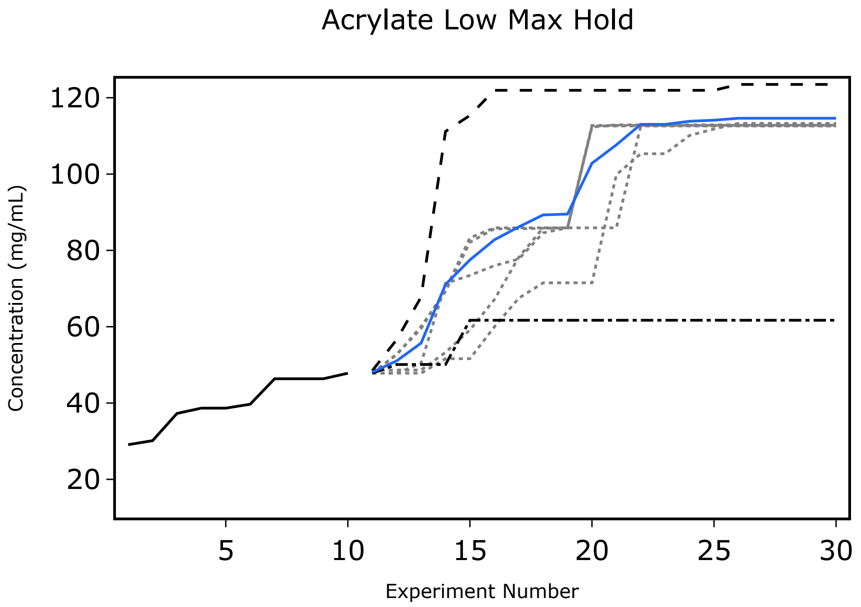

Figure S30: Ciprofloxacin intermediate digital twin case study 1 max-hold transform of raw data

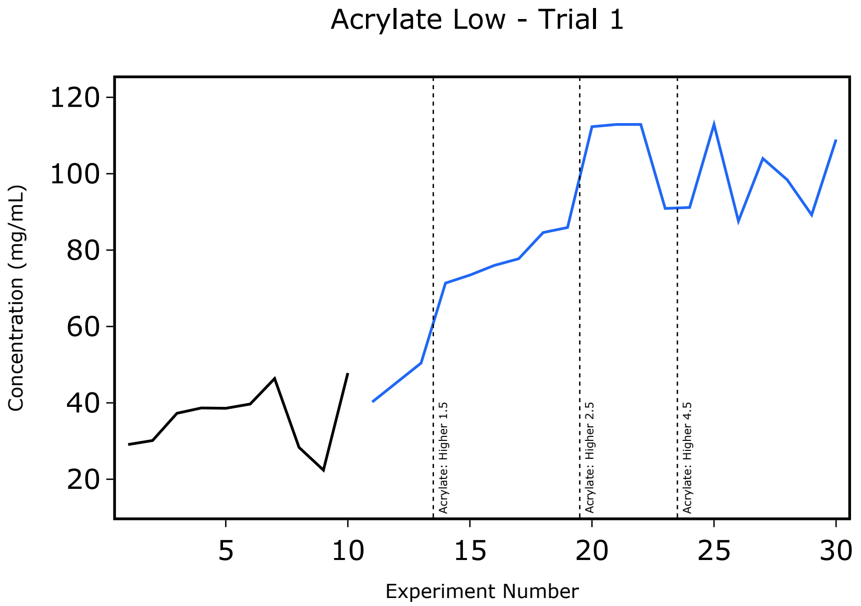

Figure S31: Ciprofloxacin intermediate digital twin case study 1 trial 1 raw data with expansion annotations

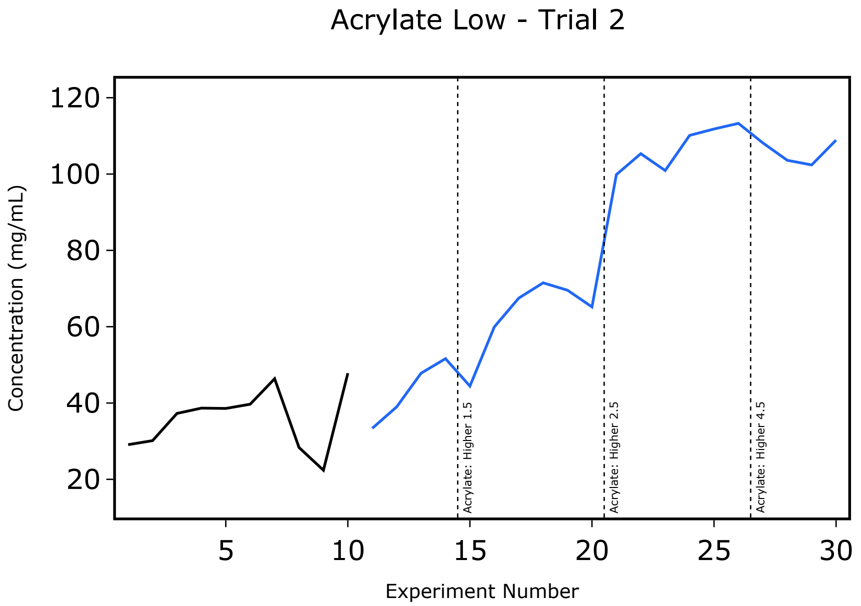

Figure S32: Ciprofloxacin intermediate digital twin case study 1 trial 2 raw data with expansion annotations

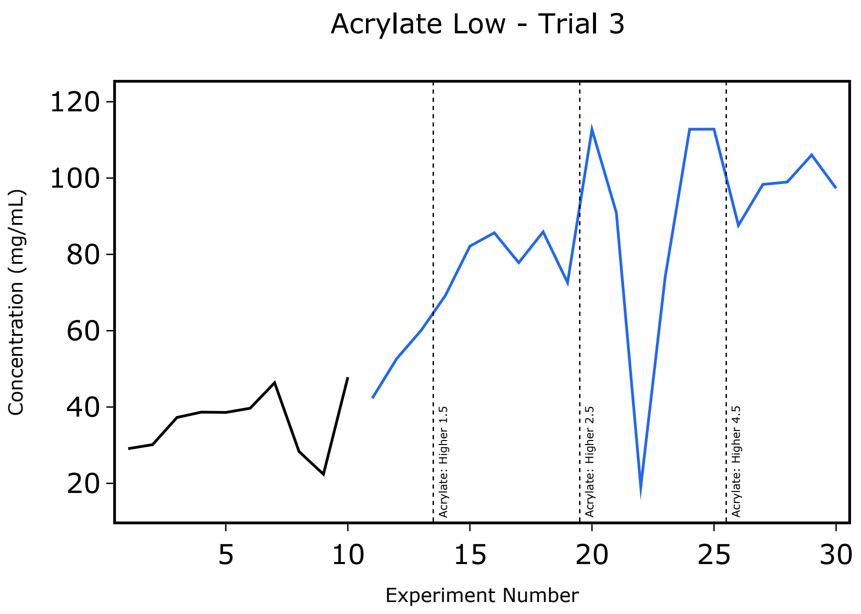

Figure S33: Ciprofloxacin intermediate digital twin case study 1 trial 3 raw data with expansion annotations

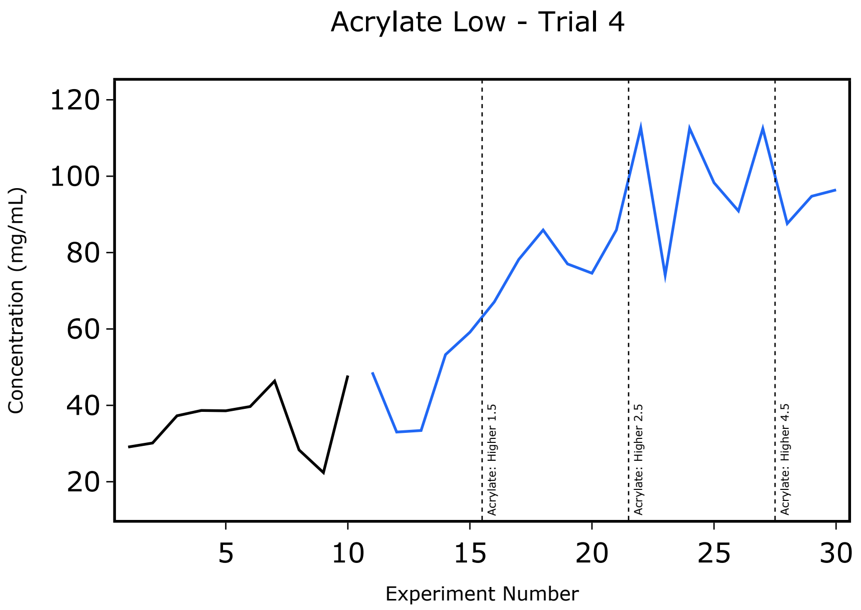

Figure S34: Ciprofloxacin intermediate digital twin case study 1 trial 4 raw data with expansion annotations

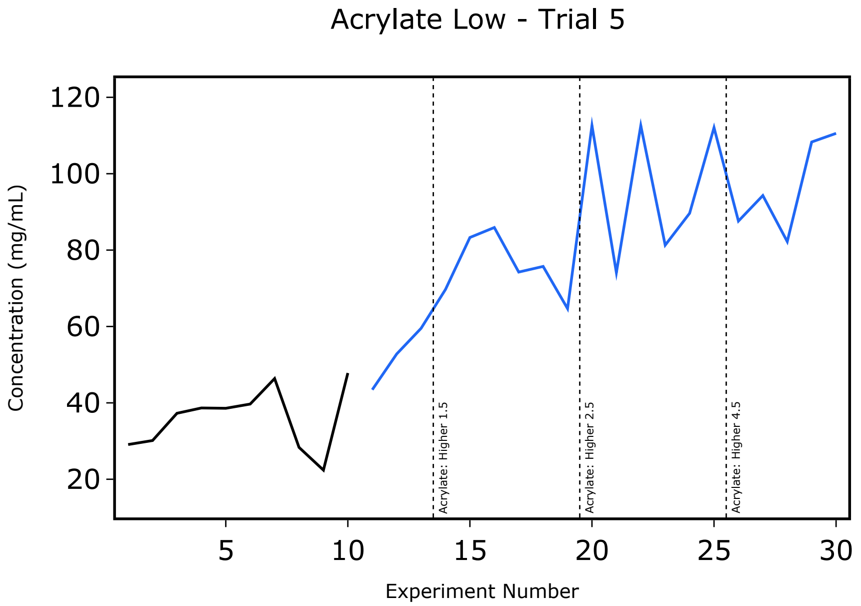

Figure S35: Ciprofloxacin intermediate digital twin case study 1 trial 5 raw data with expansion annotations

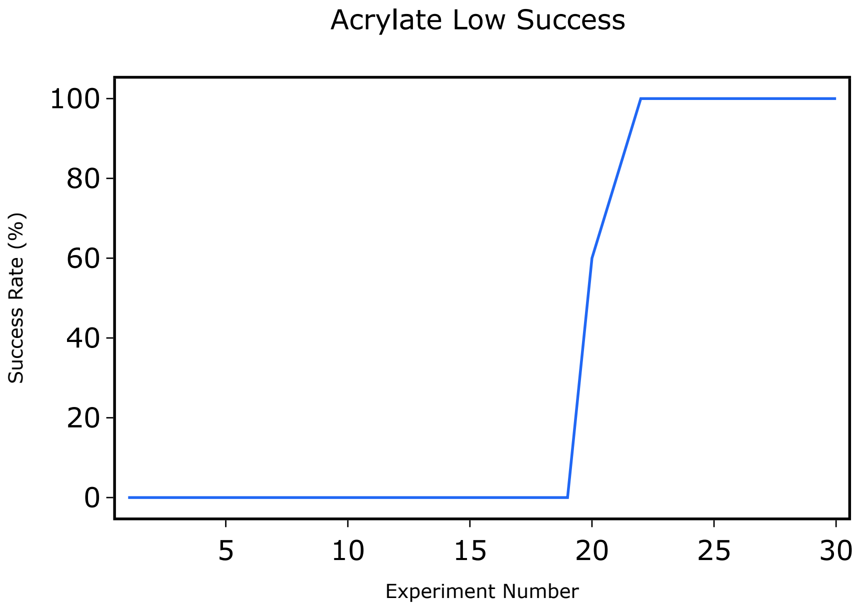

Figure S36: Ciprofloxacin intermediate digital twin case study 1 success rate plot

Case study 2 result figures

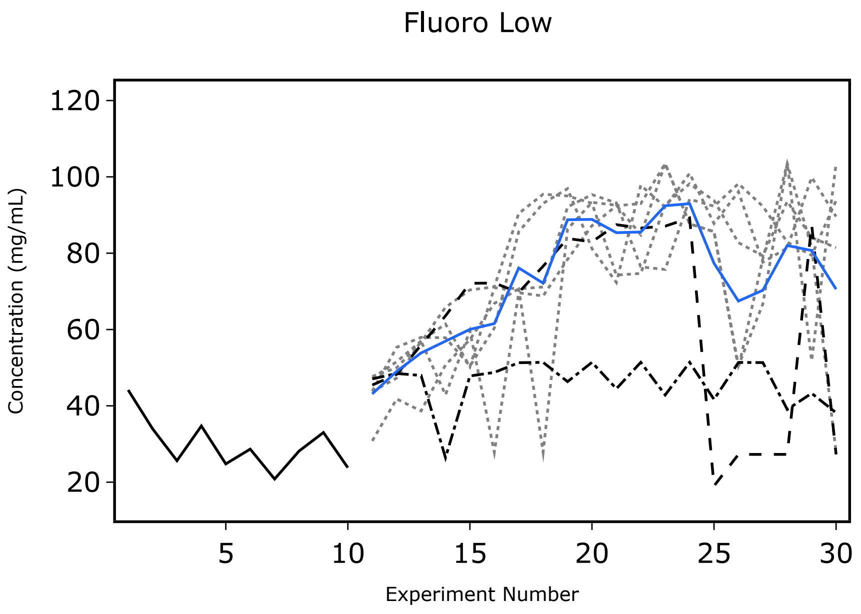

Figure S37: Ciprofloxacin intermediate digital twin case study 2 raw data

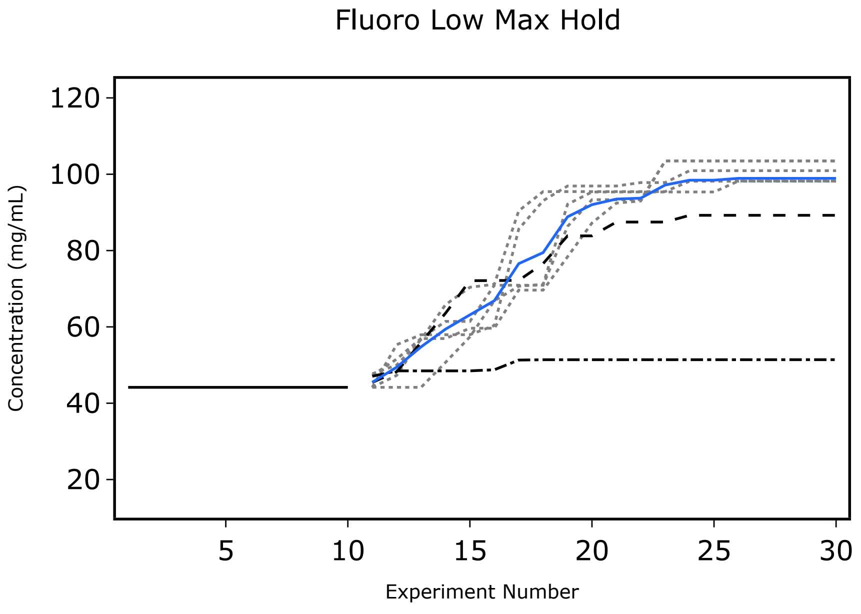

Figure S38: Ciprofloxacin intermediate digital twin case study 2 max-hold transform of raw data

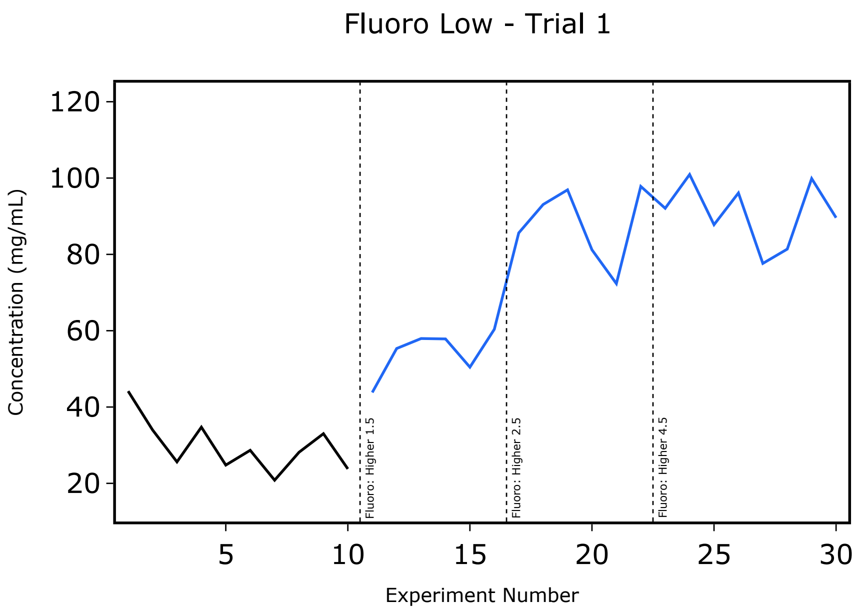

Figure S39: Ciprofloxacin intermediate digital twin case study 2 trial 1 raw data with expansion annotations

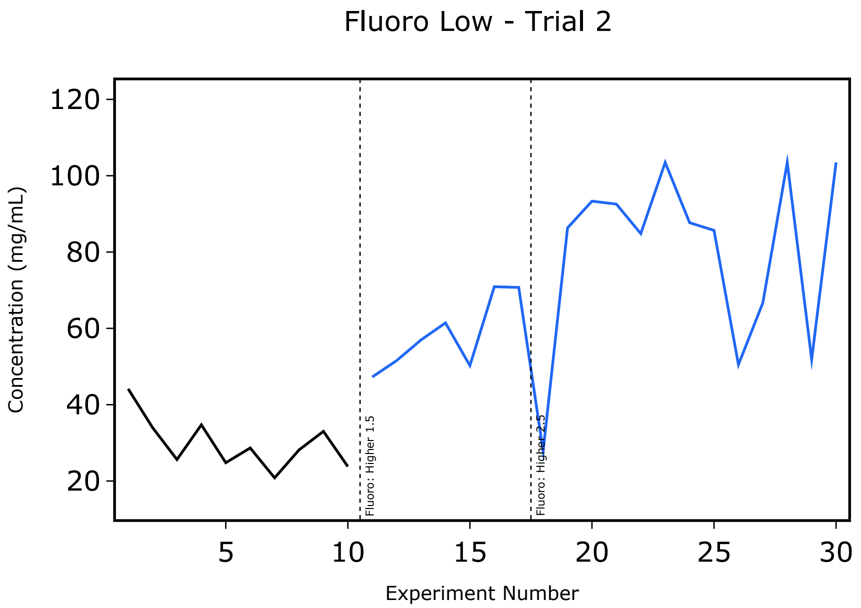

Figure S40: Ciprofloxacin intermediate digital twin case study 2 trial 2 raw data with expansion annotations

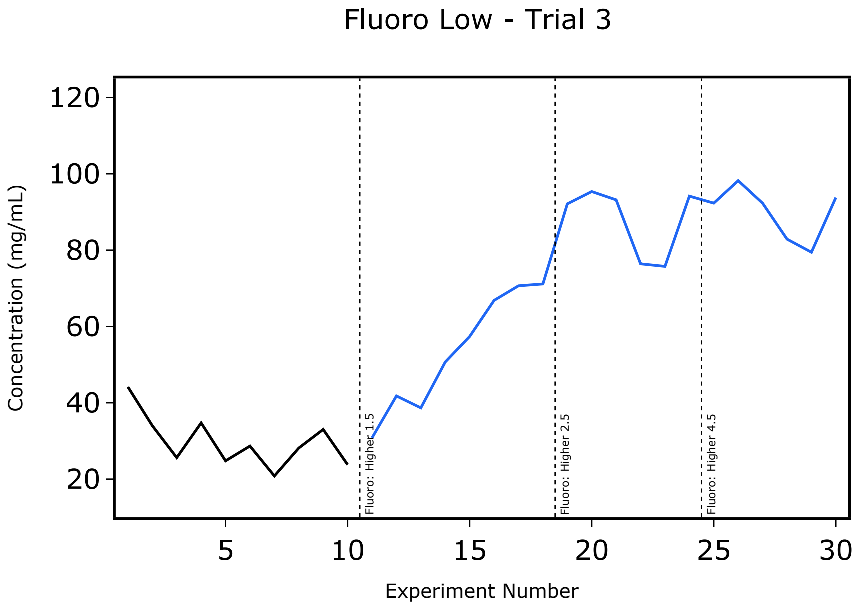

Figure S41: Ciprofloxacin intermediate digital twin case study 2 trial 3 raw data with expansion annotations

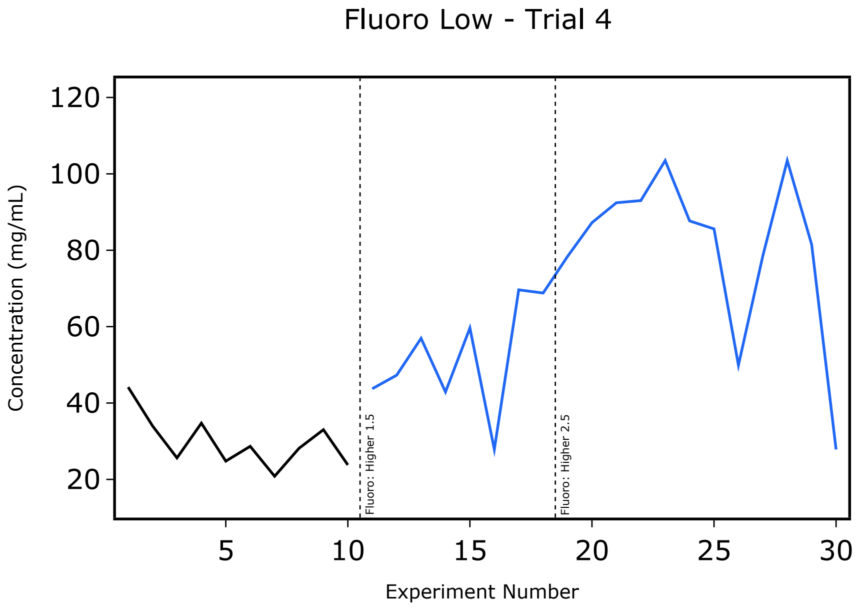

Figure S42: Ciprofloxacin intermediate digital twin case study 2 trial 4 raw data with expansion annotations

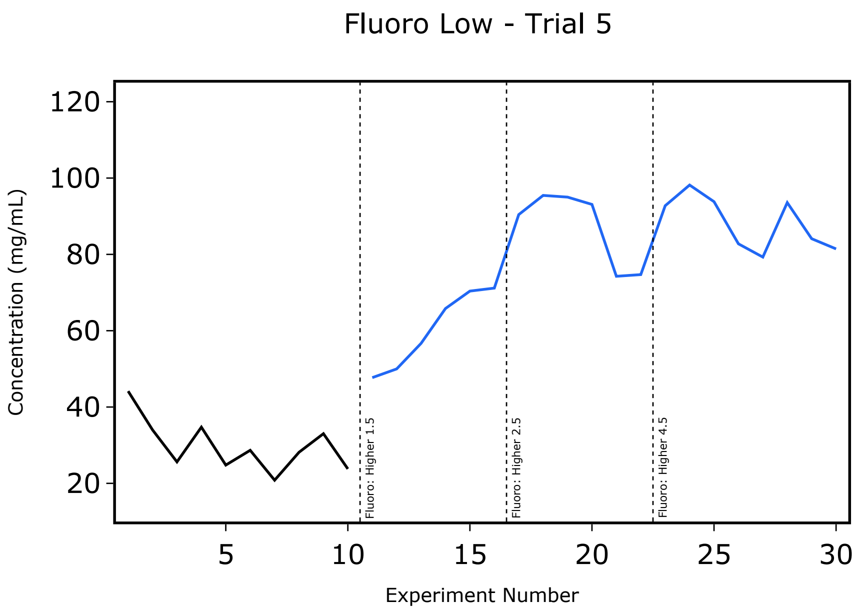

Figure S43: Ciprofloxacin intermediate digital twin case study 2 trial 5 raw data with expansion annotations

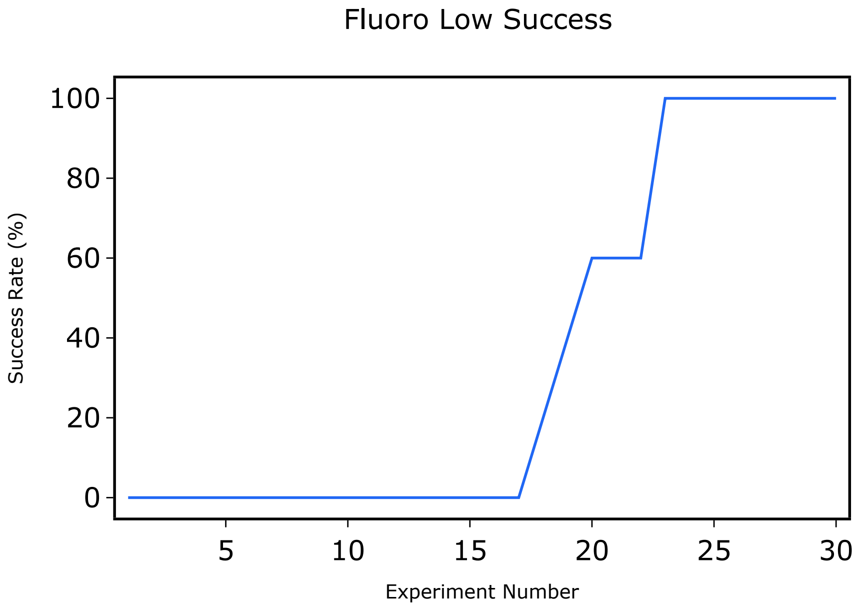

Figure S44: Ciprofloxacin intermediate digital twin case study 2 success rate plot

Case study 3 result figures

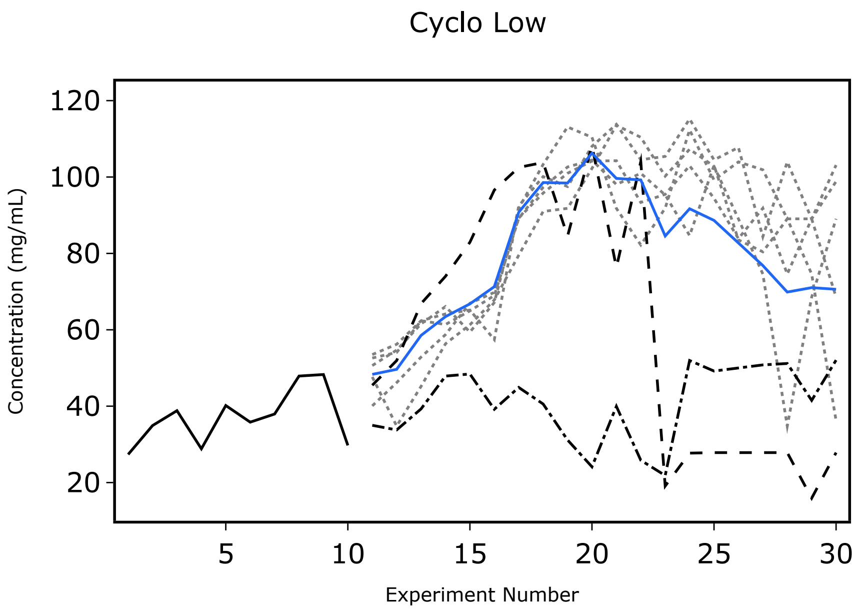

Figure S45: Ciprofloxacin intermediate digital twin case study 3 raw data

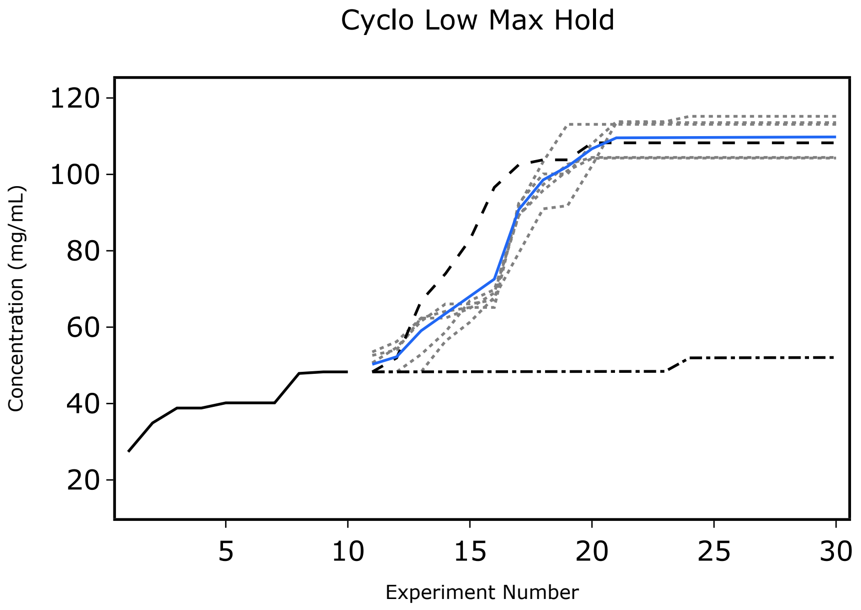

Figure S46: Ciprofloxacin intermediate digital twin case study 3 max-hold transform of raw data

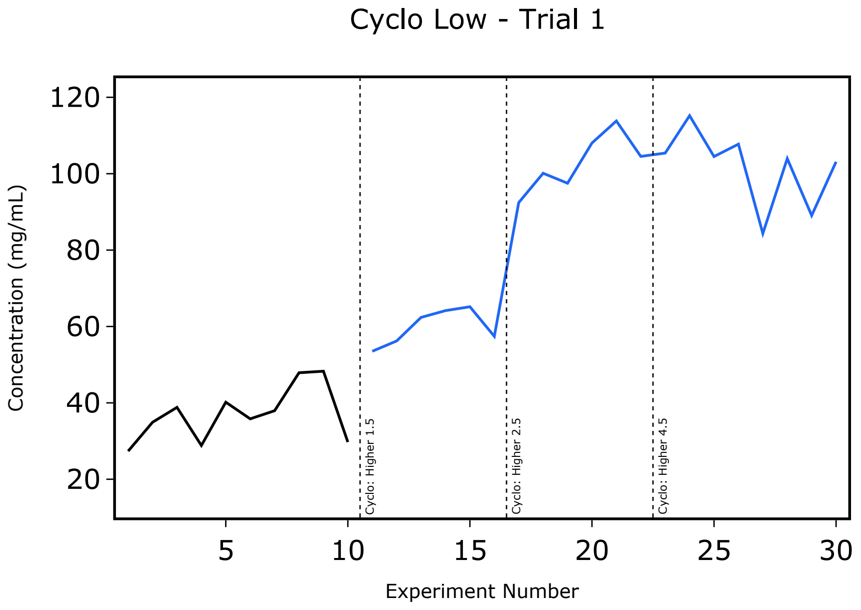

Figure S47: Ciprofloxacin intermediate digital twin case study 3 trial 1 raw data with expansion annotations

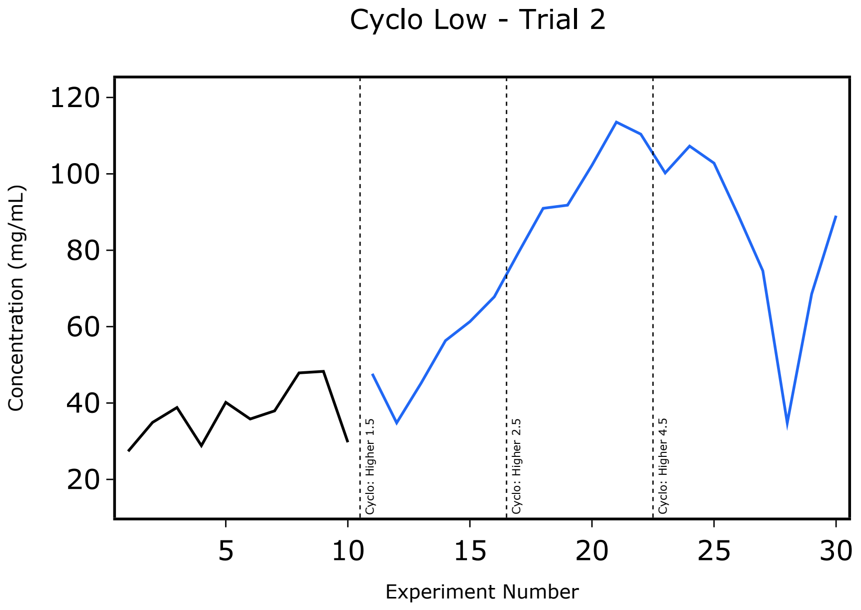

Figure S48: Ciprofloxacin intermediate digital twin case study 3 trial 2 raw data with expansion annotations

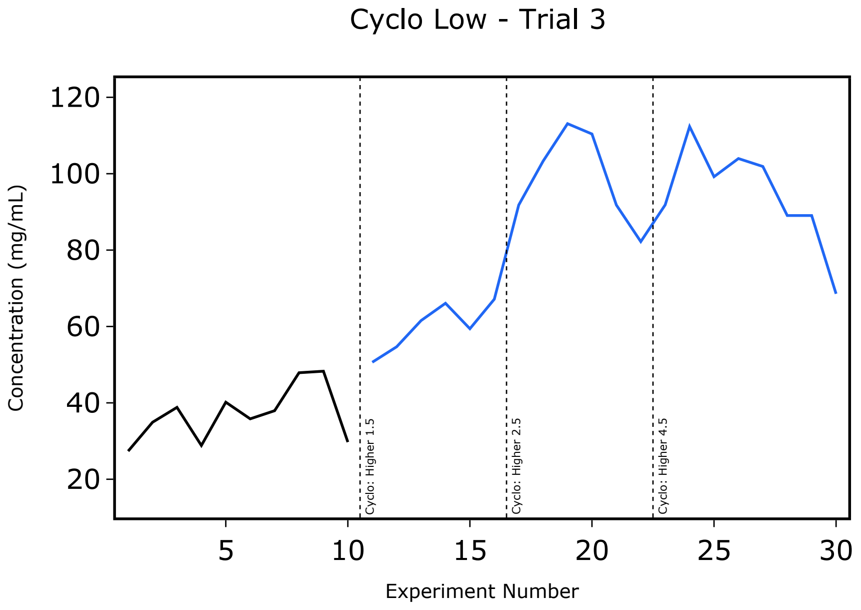

Figure S49: Ciprofloxacin intermediate digital twin case study 3 trial 3 raw data with expansion annotations

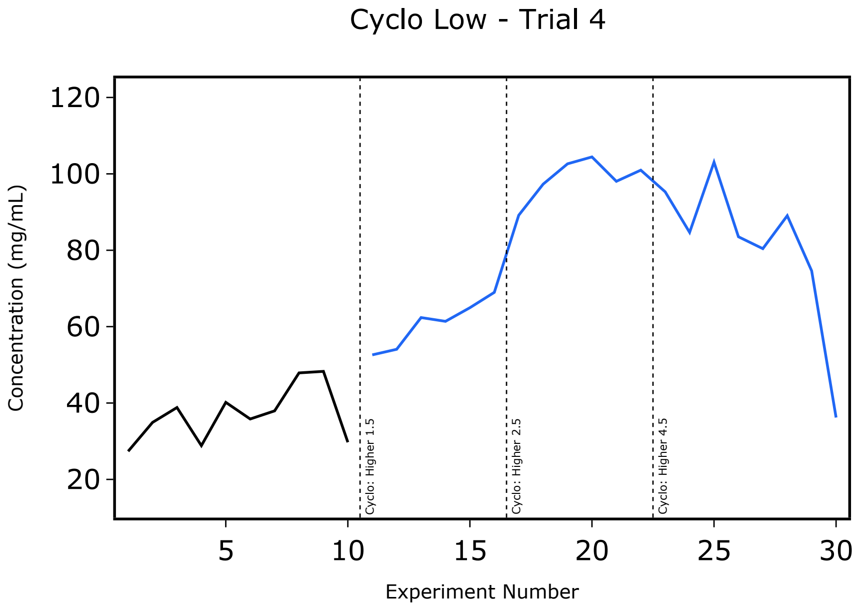

Figure S50: Ciprofloxacin intermediate digital twin case study 3 trial 4 raw data with expansion annotations

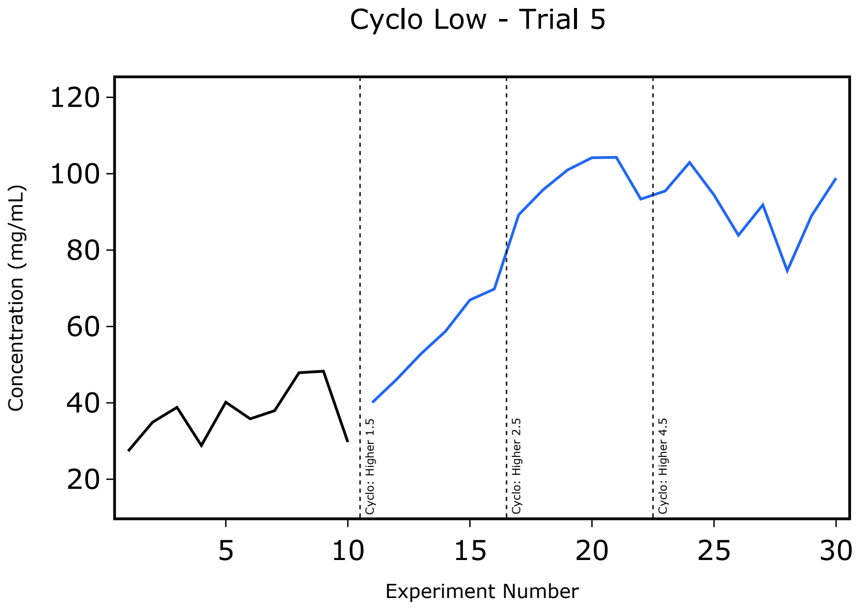

Figure S51: Ciprofloxacin intermediate digital twin case study 3 trial 5 raw data with expansion annotations

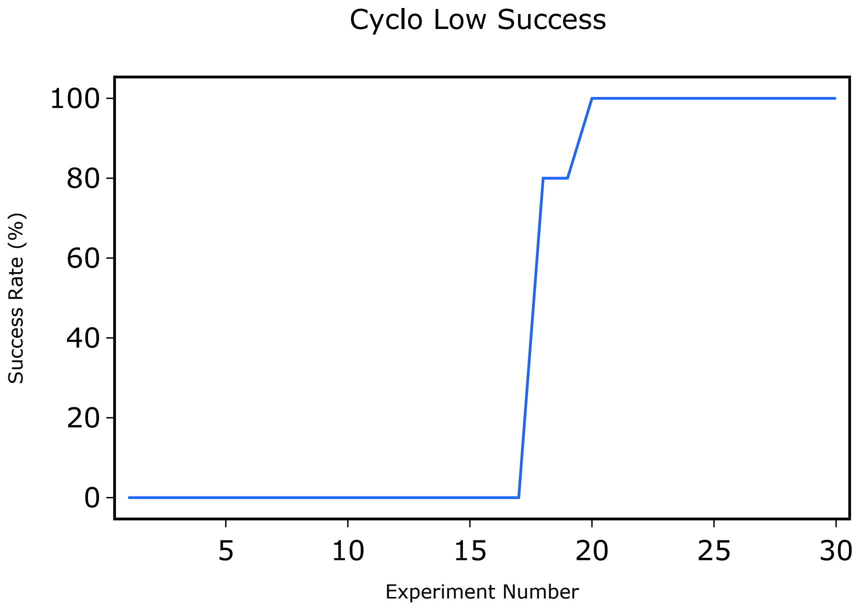

Figure S52: Ciprofloxacin intermediate digital twin case study 3 success rate plot

Case study 4 result figures

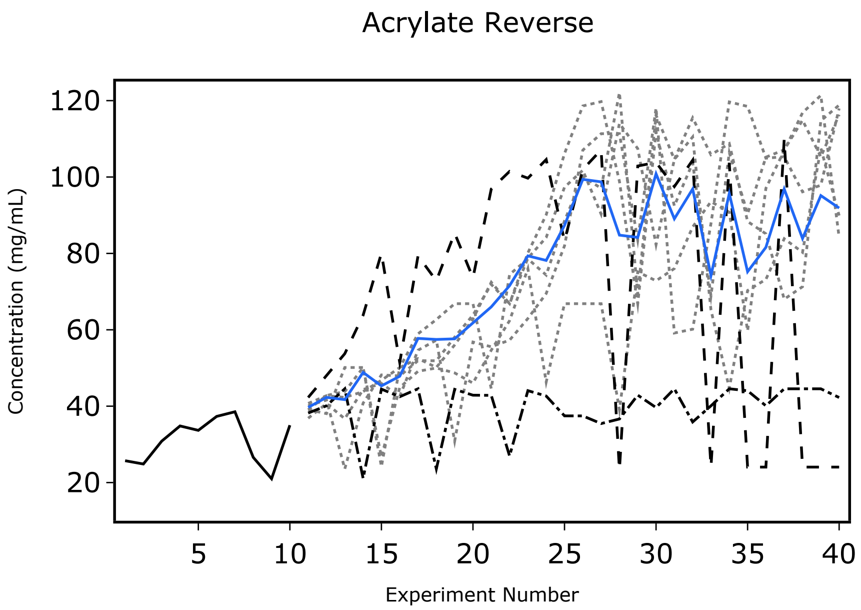

Figure S53: Ciprofloxacin intermediate digital twin case study 4 raw data

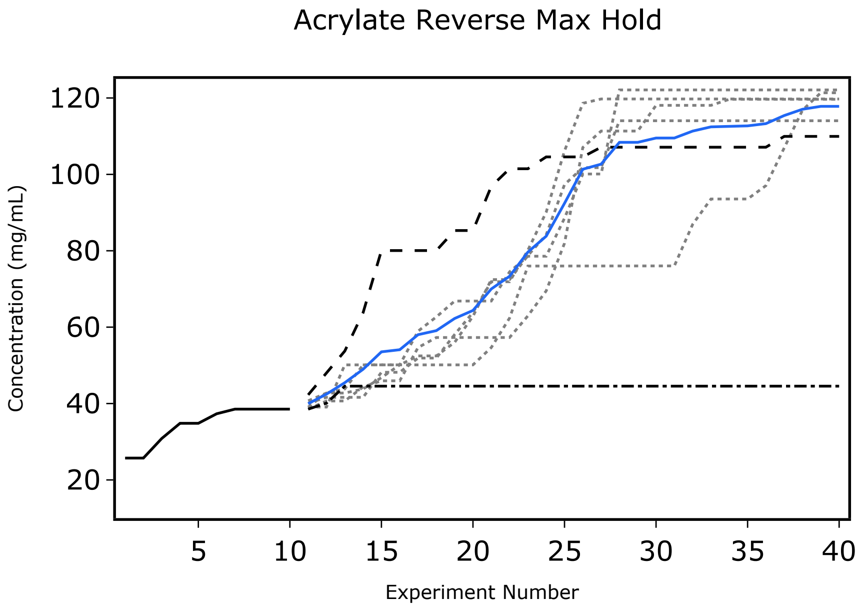

Figure S54: Ciprofloxacin intermediate digital twin case study 4 max-hold transform of raw data

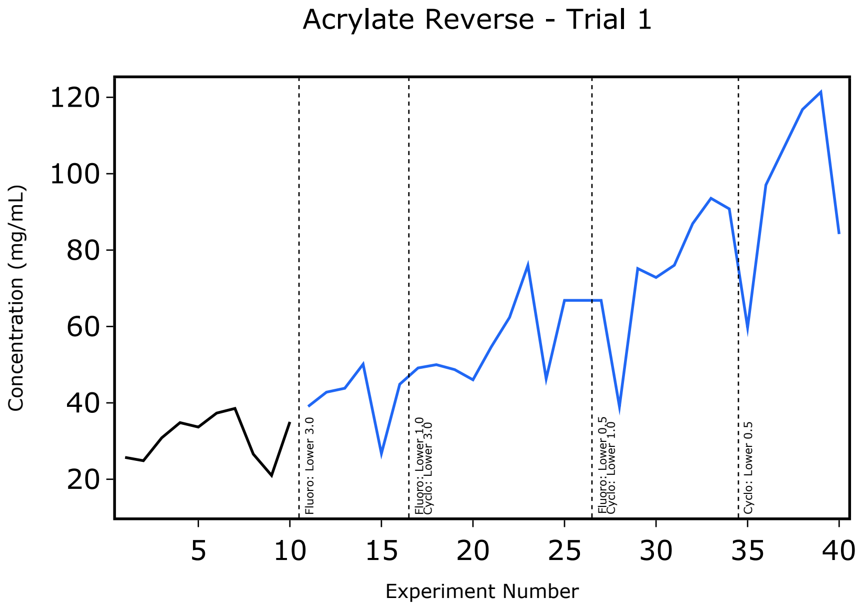

Figure S55: Ciprofloxacin intermediate digital twin case study 4 trial 1 raw data with expansion annotations

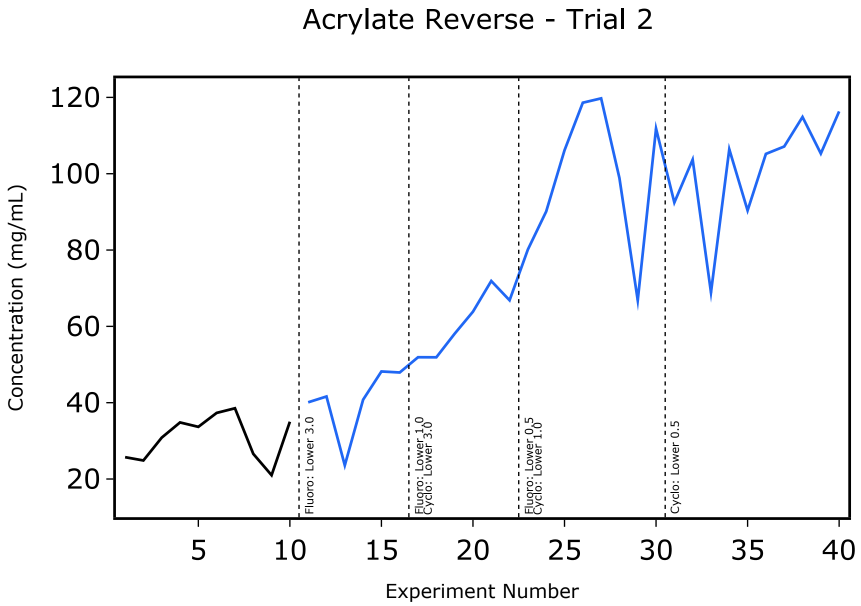

Figure S56: Ciprofloxacin intermediate digital twin case study 4 trial 2 raw data with expansion annotations

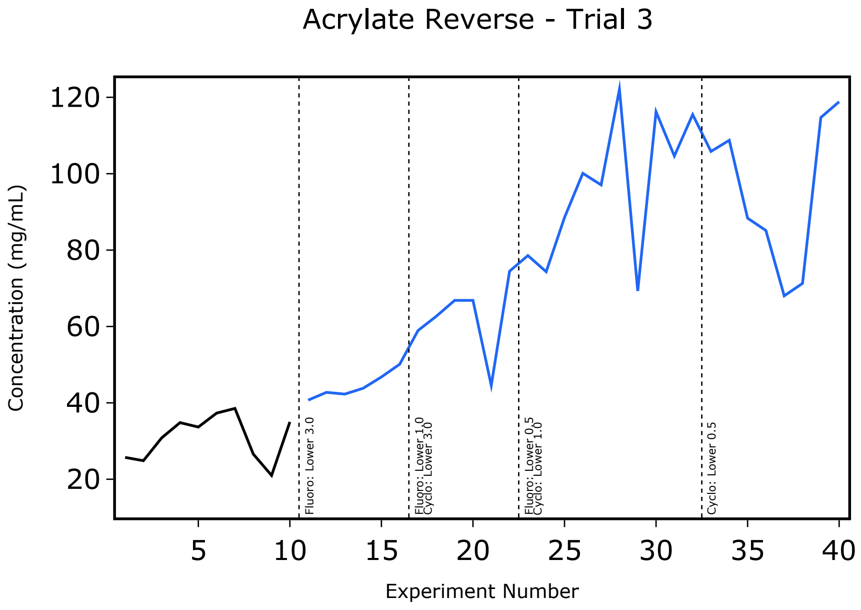

Figure S57: Ciprofloxacin intermediate digital twin case study 4 trial 3 raw data with expansion annotations

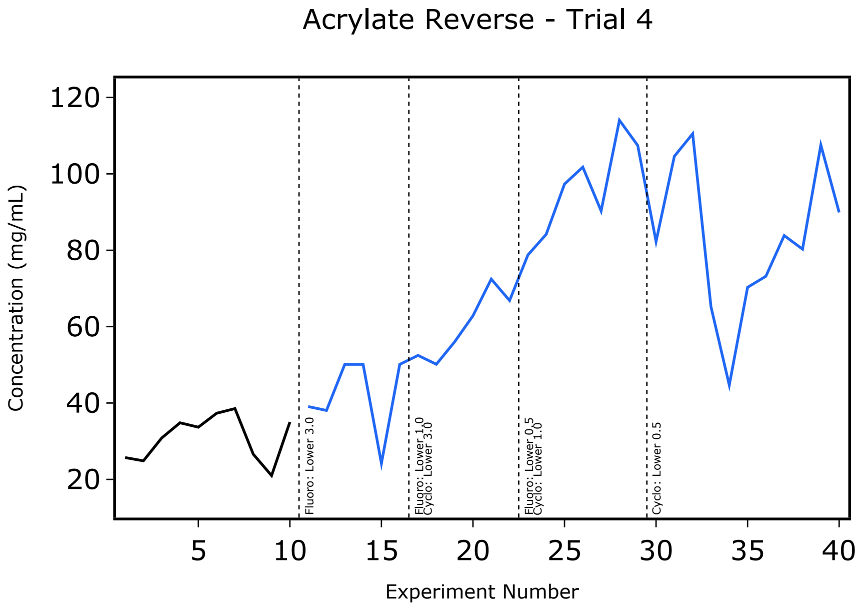

Figure S58: Ciprofloxacin intermediate digital twin case study 4 trial 4 raw data with expansion annotations

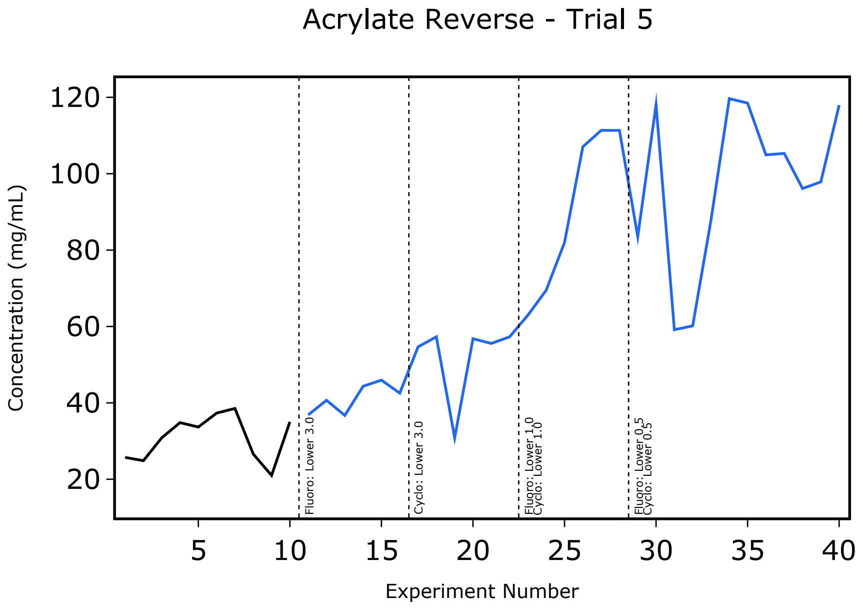

Figure S59: Ciprofloxacin intermediate digital twin case study 4 trial 5 raw data with expansion annotations

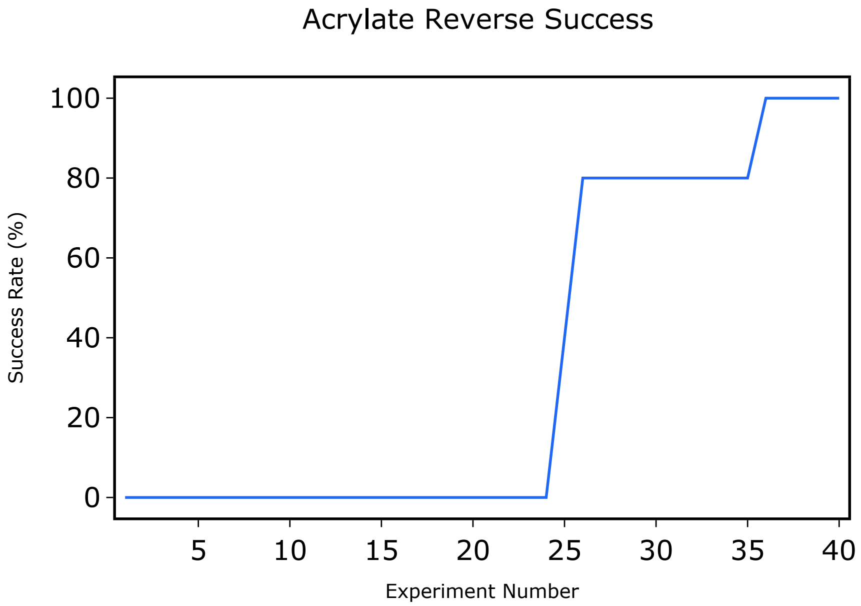

Figure S60: Ciprofloxacin intermediate digital twin case study 4 success rate plot

Case study 5 result figures

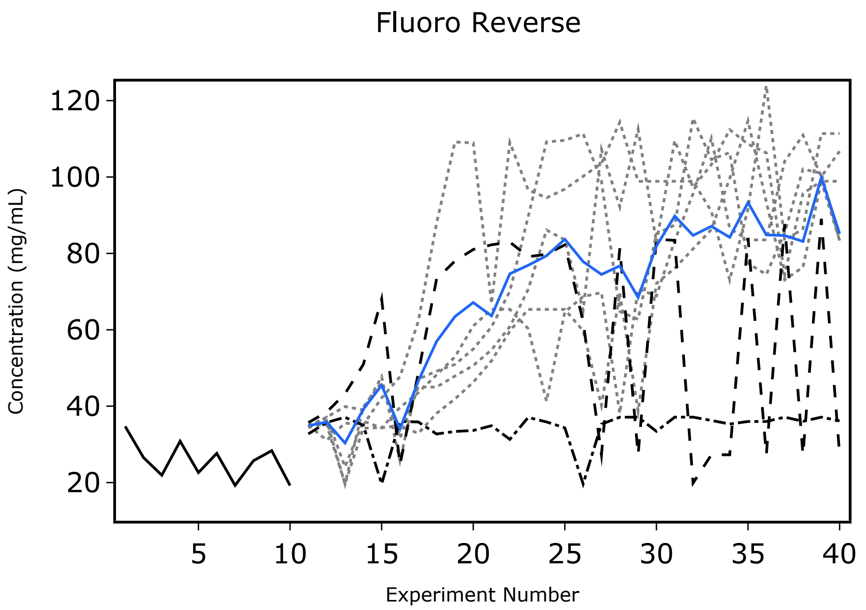

Figure S61: Ciprofloxacin intermediate digital twin case study 5 raw data

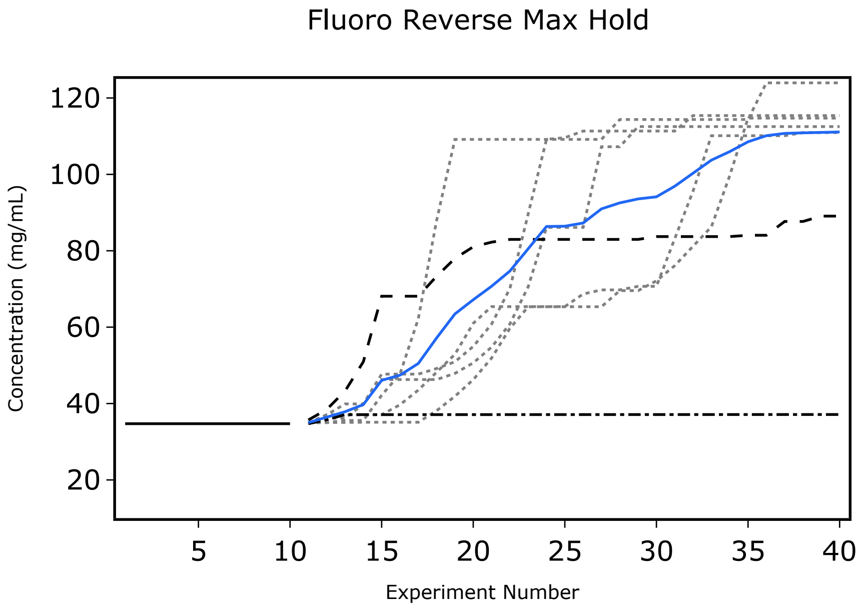

Figure S62: Ciprofloxacin intermediate digital twin case study 5 max-hold transform of raw data

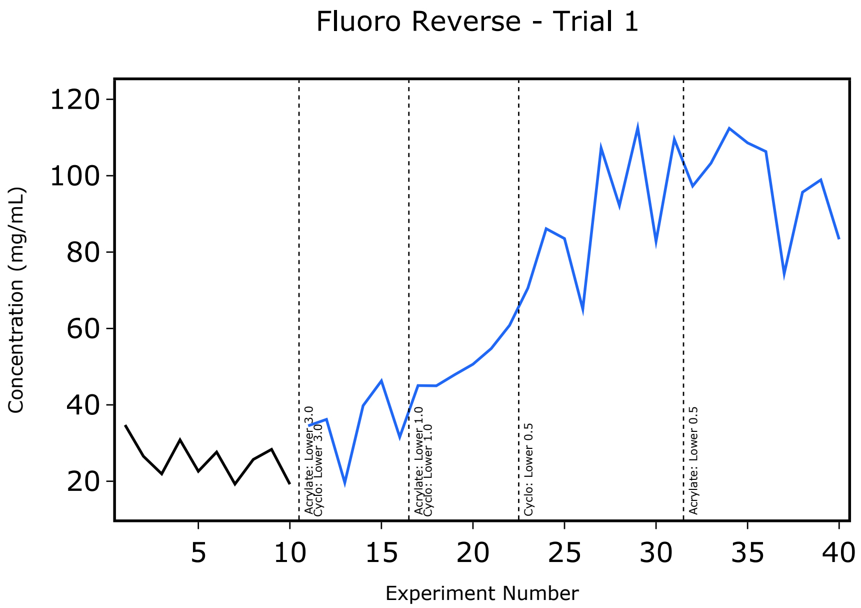

Figure S63: Ciprofloxacin intermediate digital twin case study 5 trial 1 raw data with expansion annotations

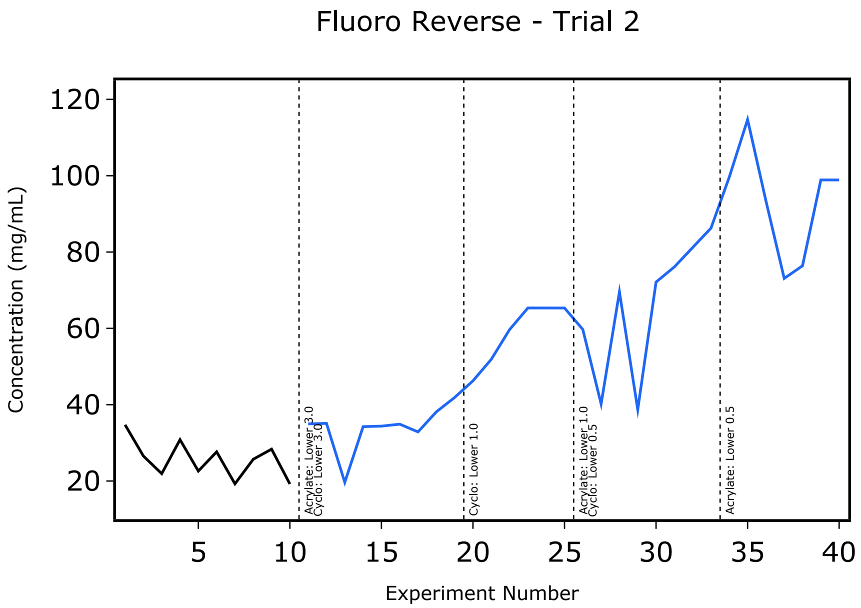

Figure S64: Ciprofloxacin intermediate digital twin case study 5 trial 2 raw data with expansion annotations

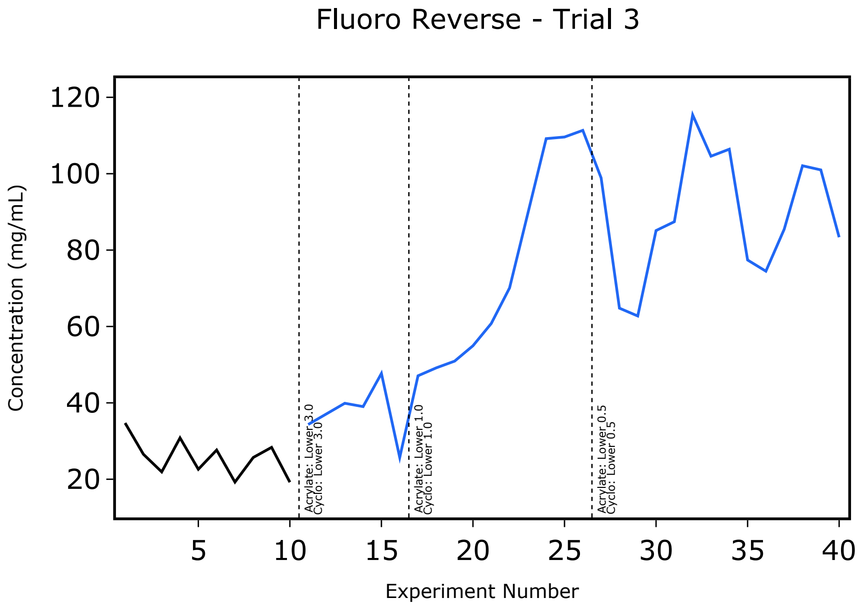

Figure S65: Ciprofloxacin intermediate digital twin case study 5 trial 3 raw data with expansion annotations

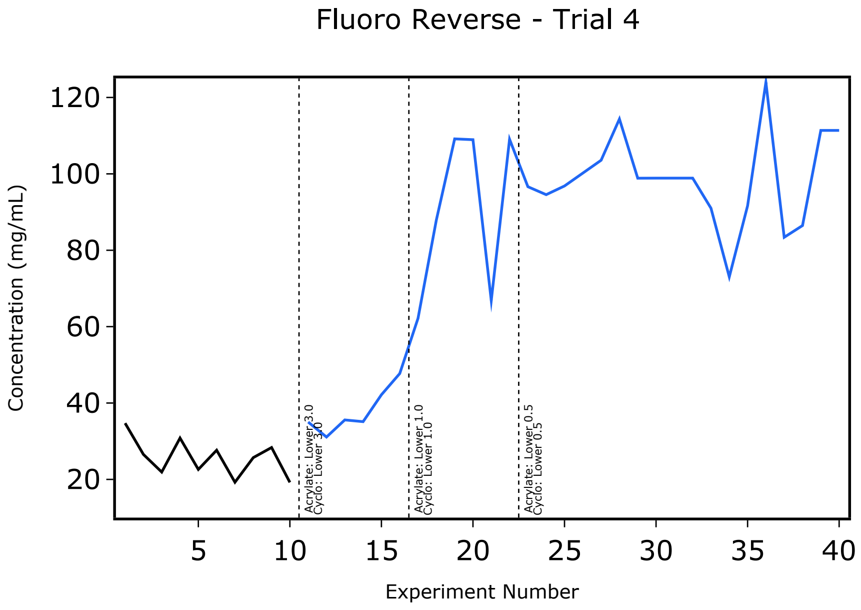

Figure S66: Ciprofloxacin intermediate digital twin case study 5 trial 4 raw data with expansion annotations

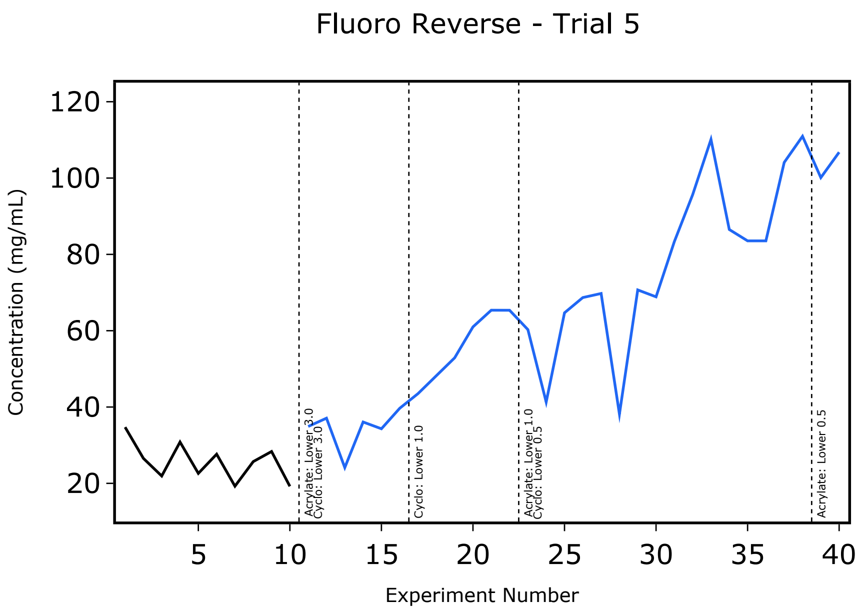

Figure S67: Ciprofloxacin intermediate digital twin case study 5 trial 5 raw data with expansion annotations

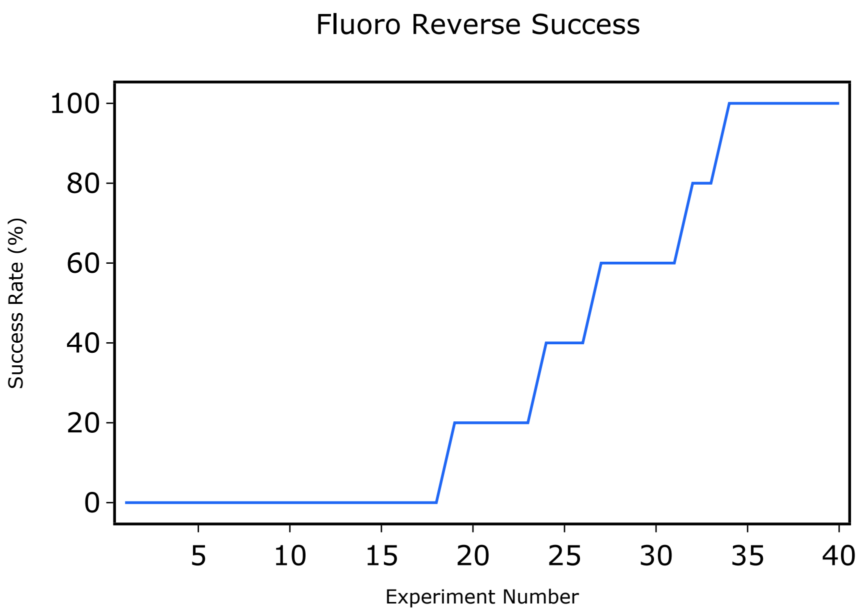

Figure S68: Ciprofloxacin intermediate digital twin case study 5 success rate plot

Case study 6 result figures

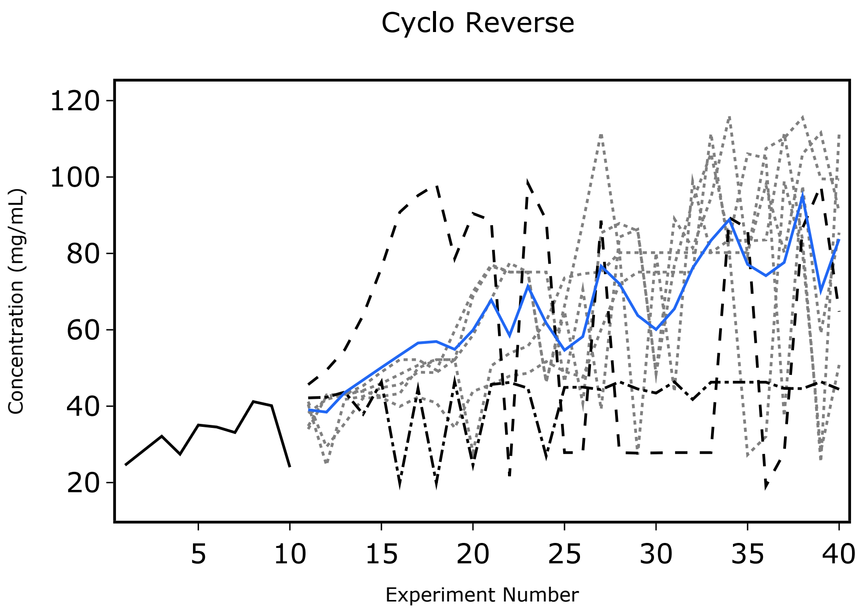

Figure S69: Ciprofloxacin intermediate digital twin case study 6 raw data

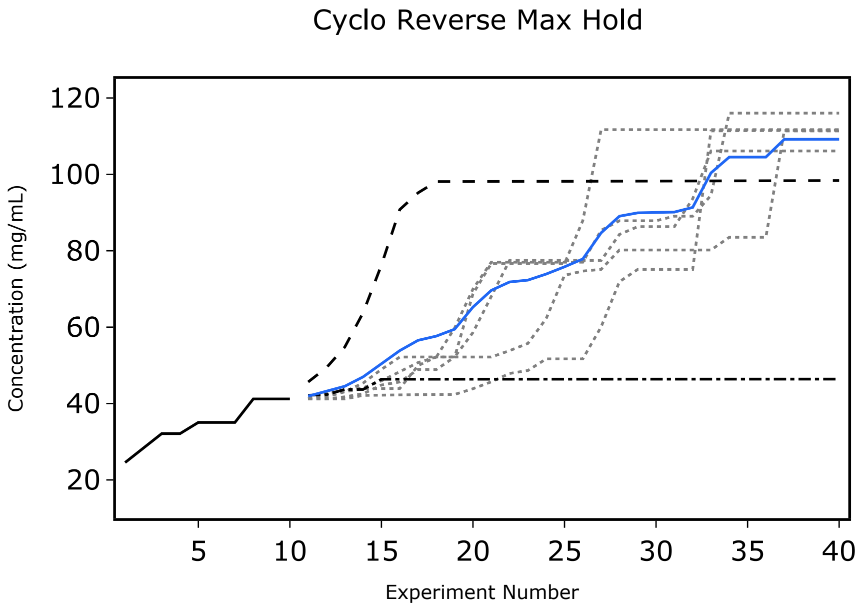

Figure S70: Ciprofloxacin intermediate digital twin case study 6 max-hold transform of raw data

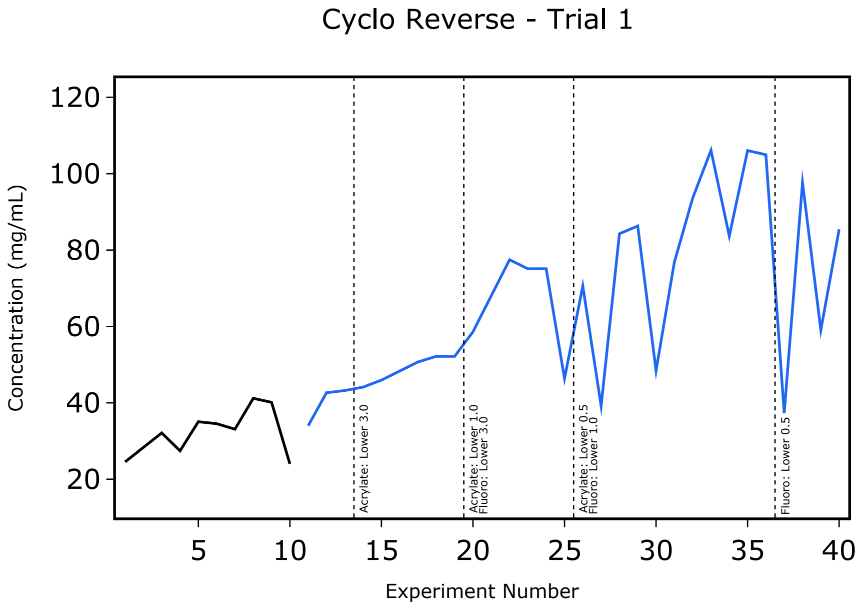

Figure S71: Ciprofloxacin intermediate digital twin case study 6 trial 1 raw data with expansion annotations

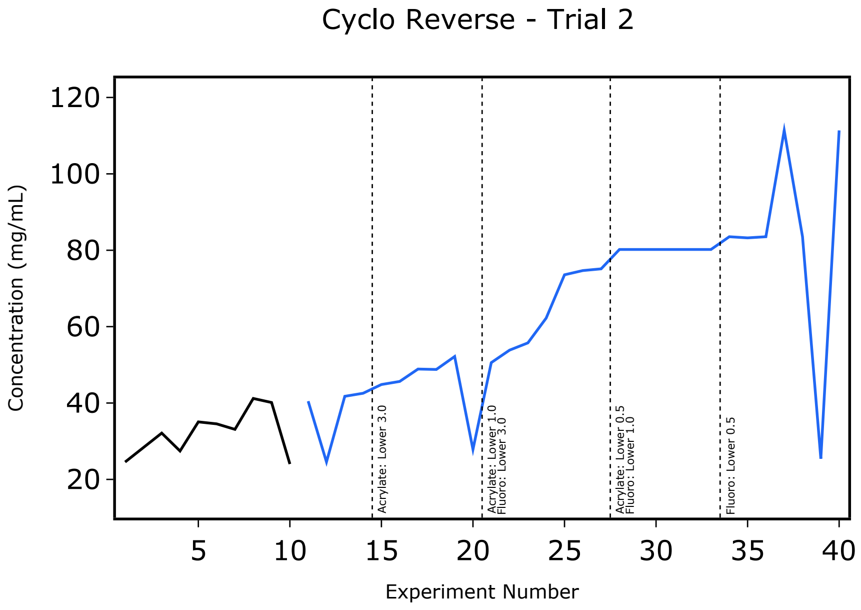

Figure S72: Ciprofloxacin intermediate digital twin case study 6 trial 2 raw data with expansion annotations

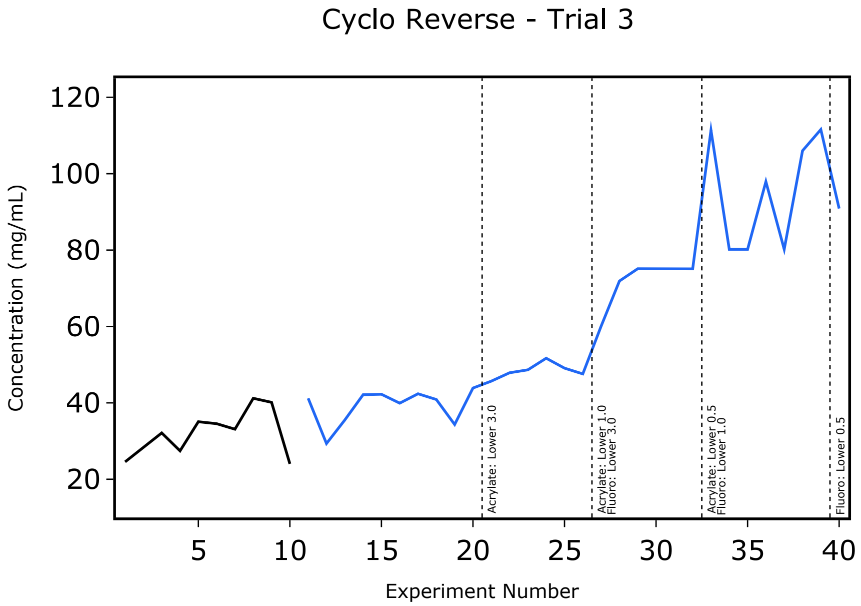

Figure S73: Ciprofloxacin intermediate digital twin case study 6 trial 3 raw data with expansion annotations

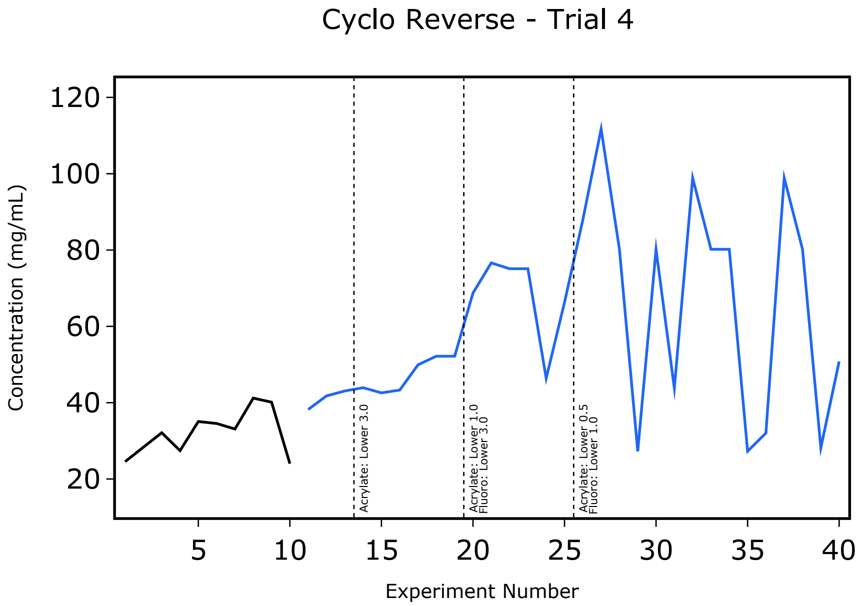

Figure S74: Ciprofloxacin intermediate digital twin case study 6 trial 4 raw data with expansion annotations

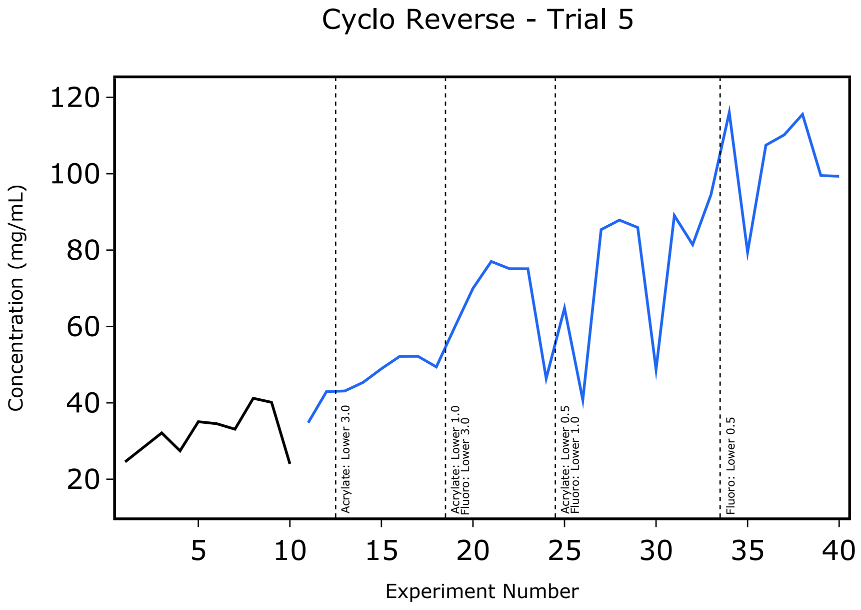

Figure S75: Ciprofloxacin intermediate digital twin case study 6 trial 5 raw data with expansion annotations

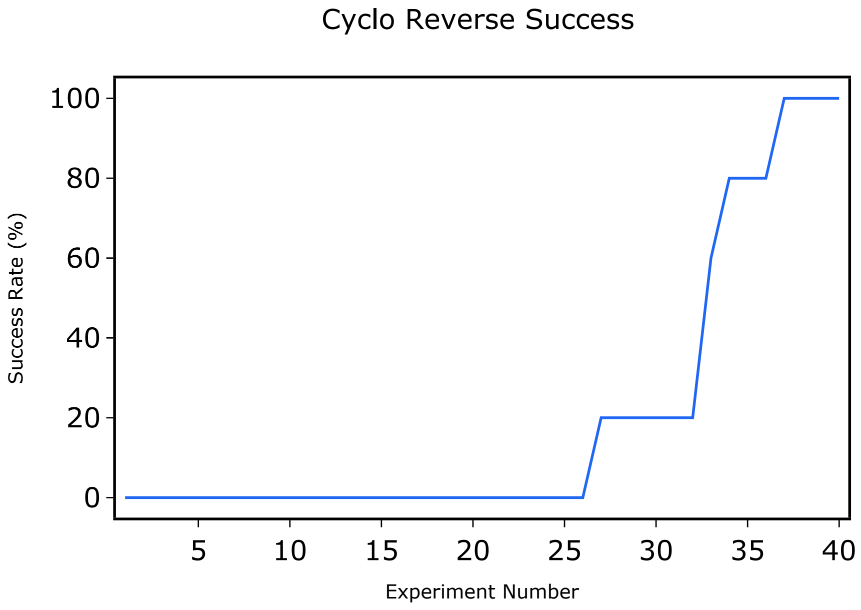

Figure S76: Ciprofloxacin intermediate digital twin case study 6 success rate plot

Case study 7 result figures

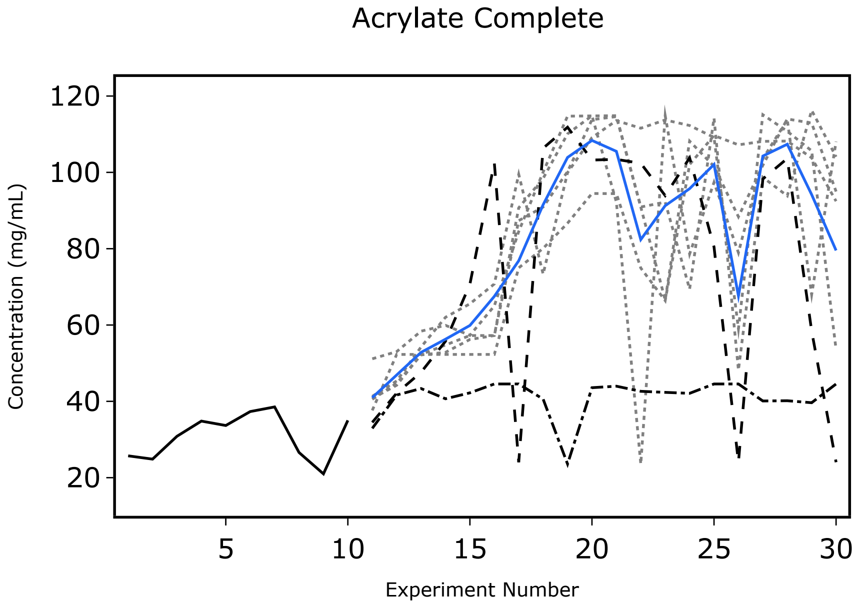

Figure S77: Ciprofloxacin intermediate digital twin case study 7 raw data

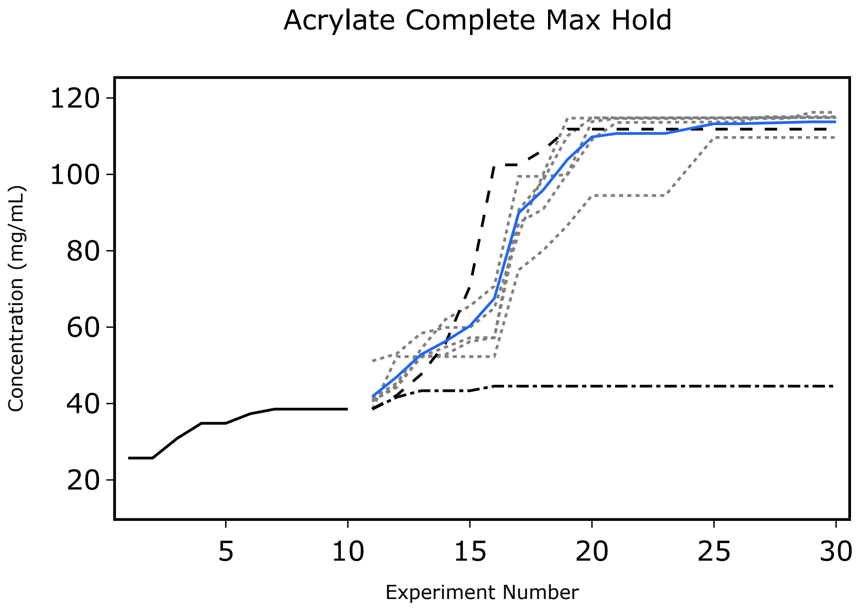

Figure S78: Ciprofloxacin intermediate digital twin case study 7 max-hold transform of raw data

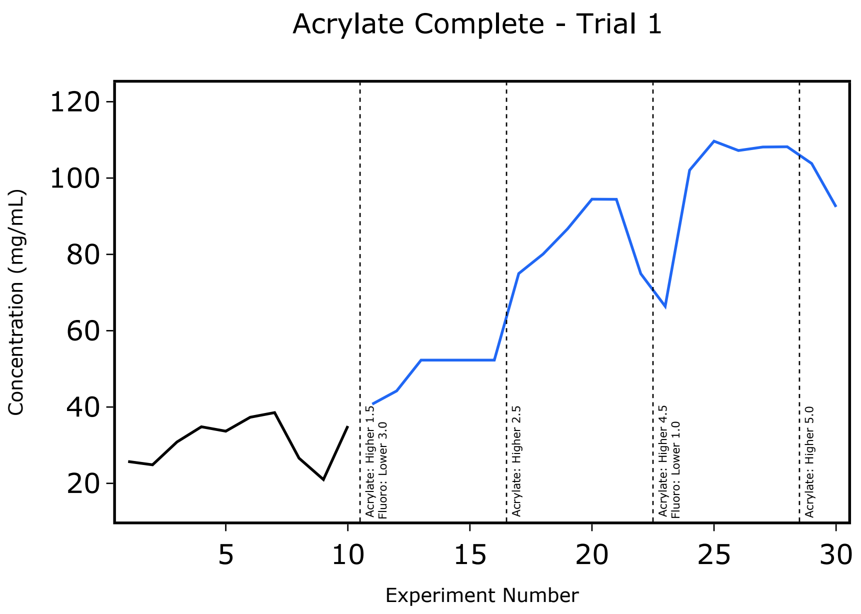

Figure S79: Ciprofloxacin intermediate digital twin case study 7 trial 1 raw data with expansion annotations

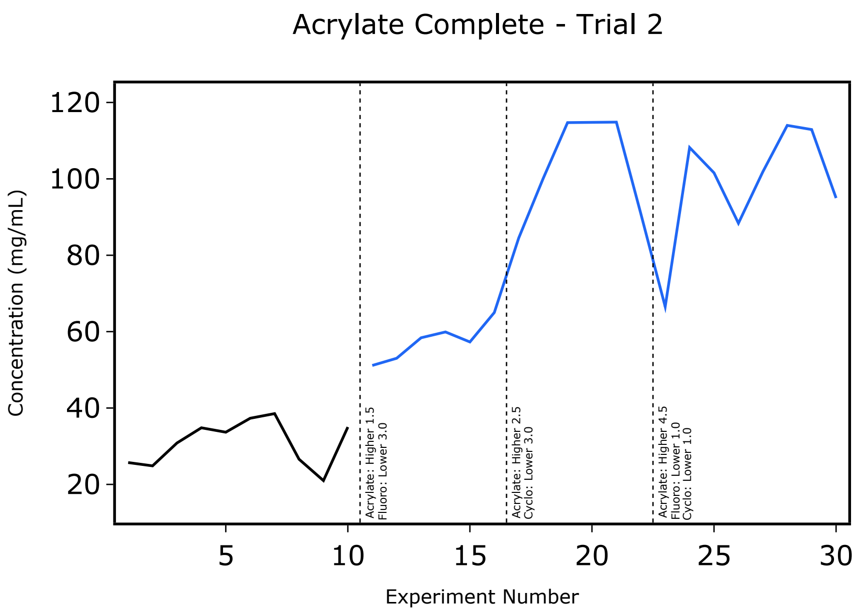

Figure S80: Ciprofloxacin intermediate digital twin case study 7 trial 2 raw data with expansion annotations

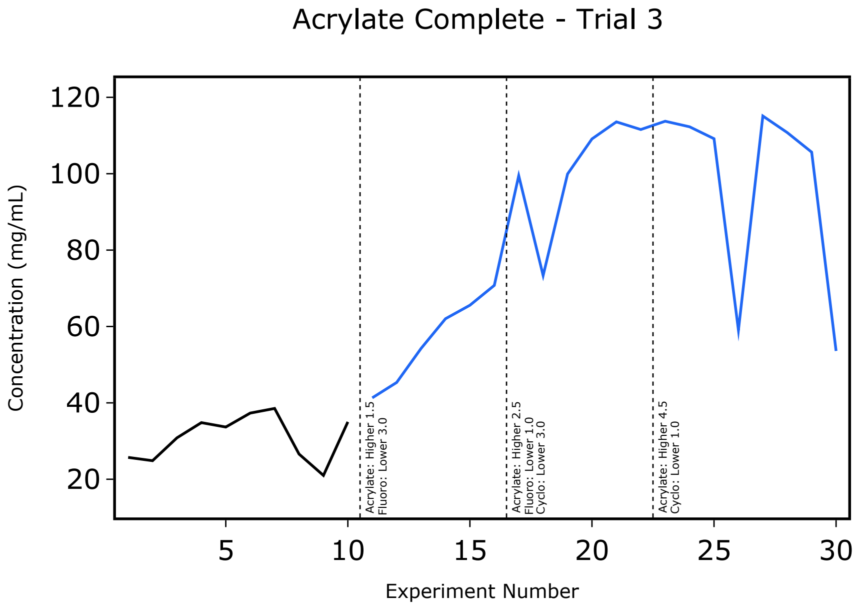

Figure S81: Ciprofloxacin intermediate digital twin case study 7 trial 3 raw data with expansion annotations

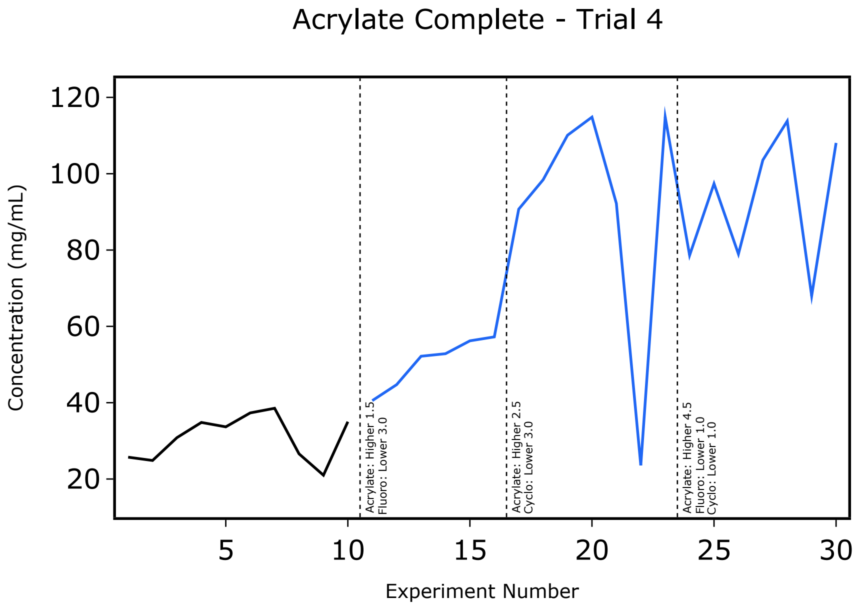

Figure S82: Ciprofloxacin intermediate digital twin case study 7 trial 4 raw data with expansion annotations

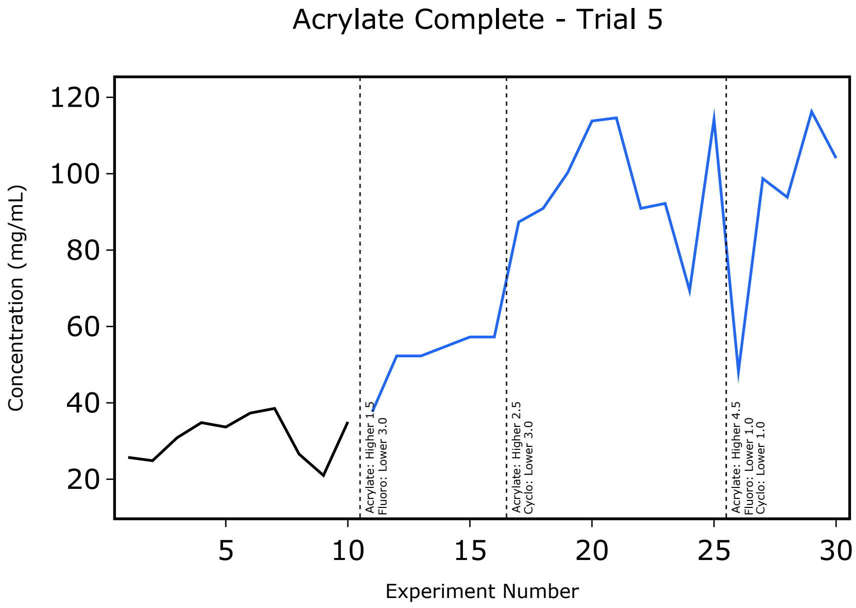

Figure S83: Ciprofloxacin intermediate digital twin case study 7 trial 5 raw data with expansion annotations

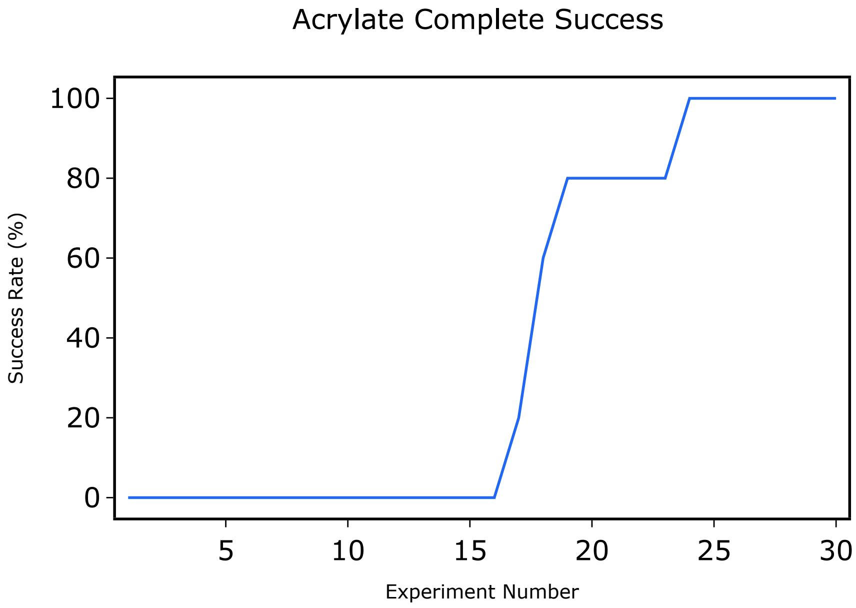

Figure S84: Ciprofloxacin intermediate digital twin case study 7 success rate plot

Case study 8 result figures

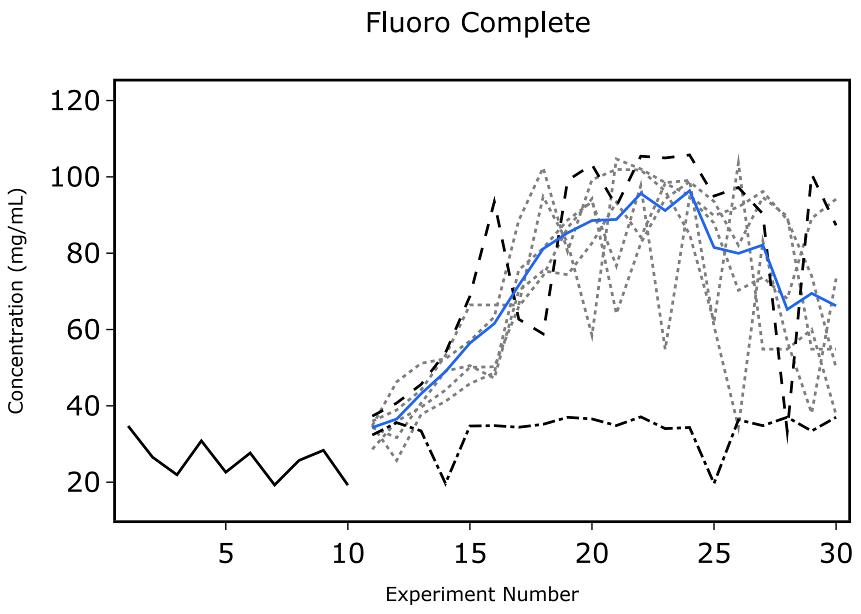

Figure S85: Ciprofloxacin intermediate digital twin case study 8 raw data

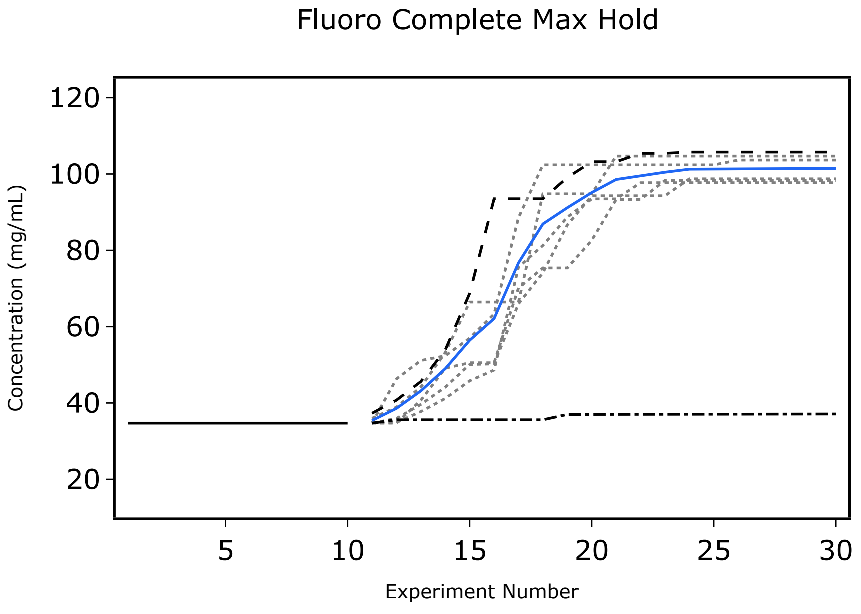

Figure S86: Ciprofloxacin intermediate digital twin case study 8 max-hold transform of raw data

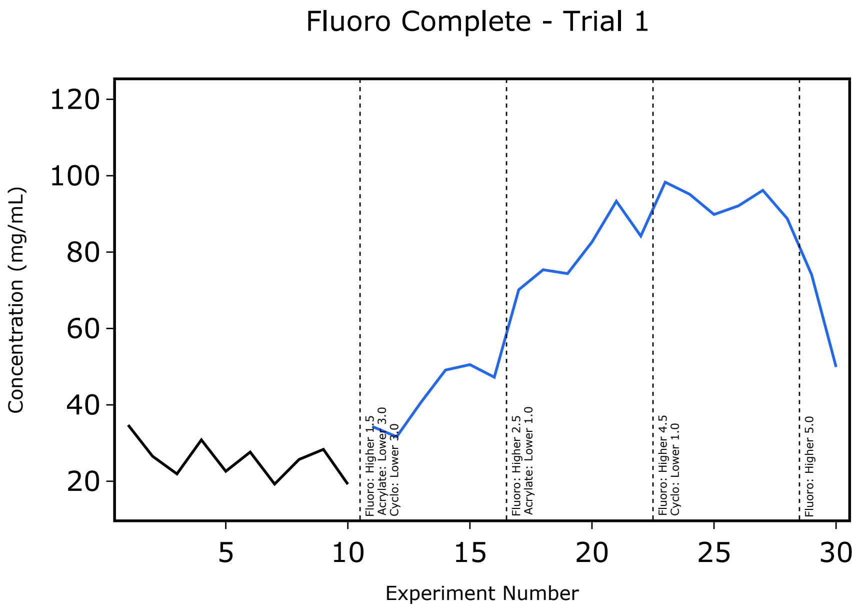

Figure S87: Ciprofloxacin intermediate digital twin case study 8 trial 1 raw data with expansion annotations

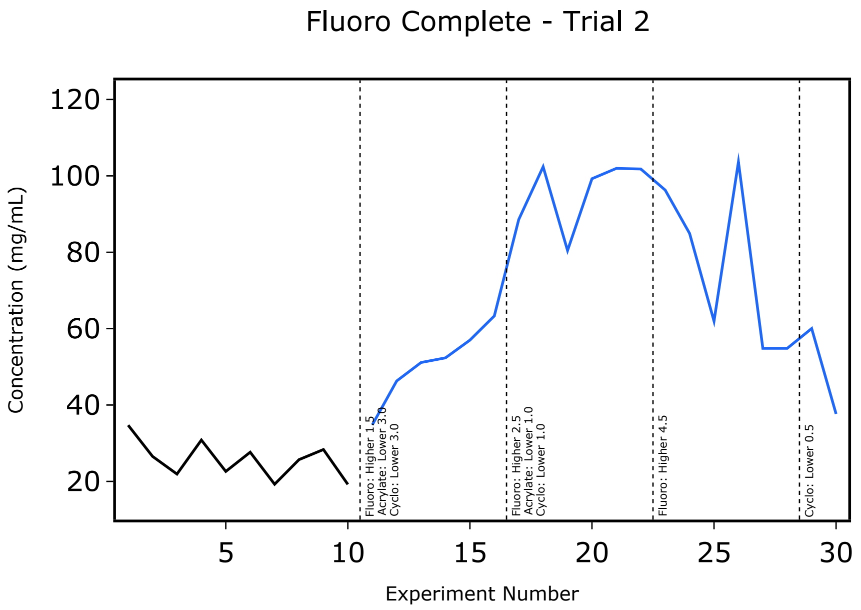

Figure S88: Ciprofloxacin intermediate digital twin case study 8 trial 2 raw data with expansion annotations

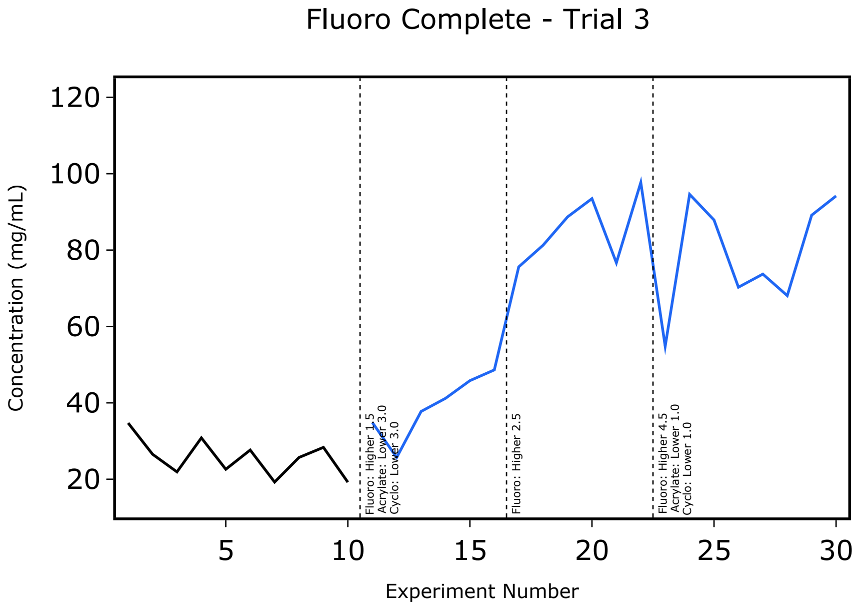

Figure S89: Ciprofloxacin intermediate digital twin case study 8 trial 3 raw data with expansion annotations

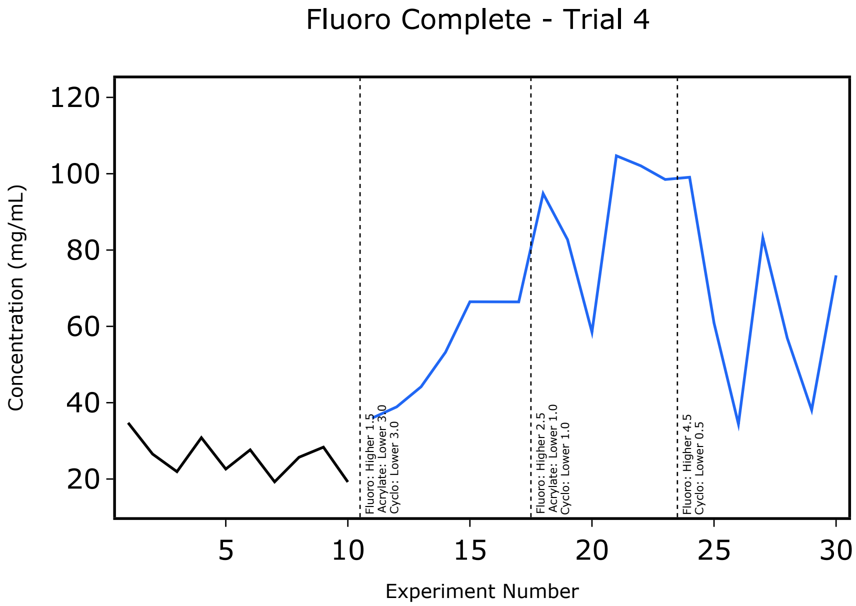

Figure S90: Ciprofloxacin intermediate digital twin case study 8 trial 4 raw data with expansion annotations

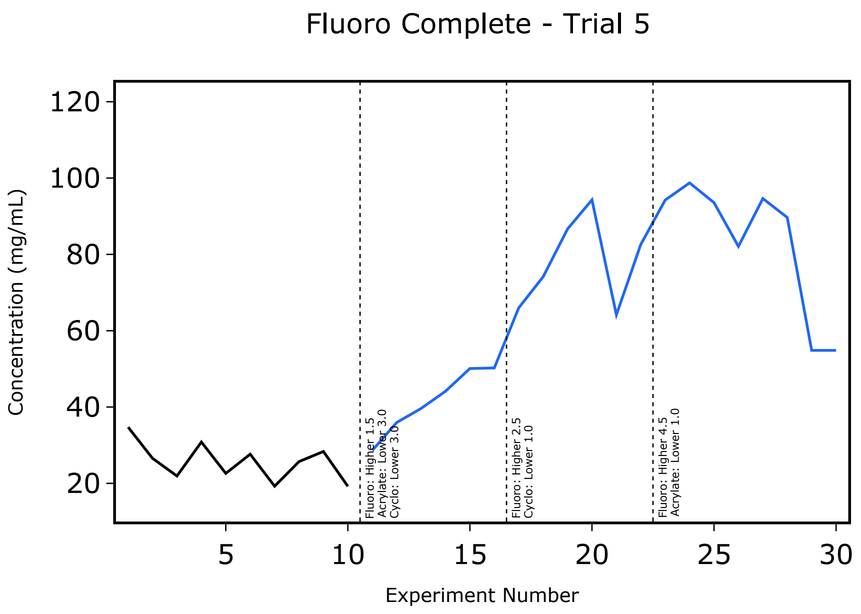

Figure S91: Ciprofloxacin intermediate digital twin case study 8 trial 5 raw data with expansion annotations

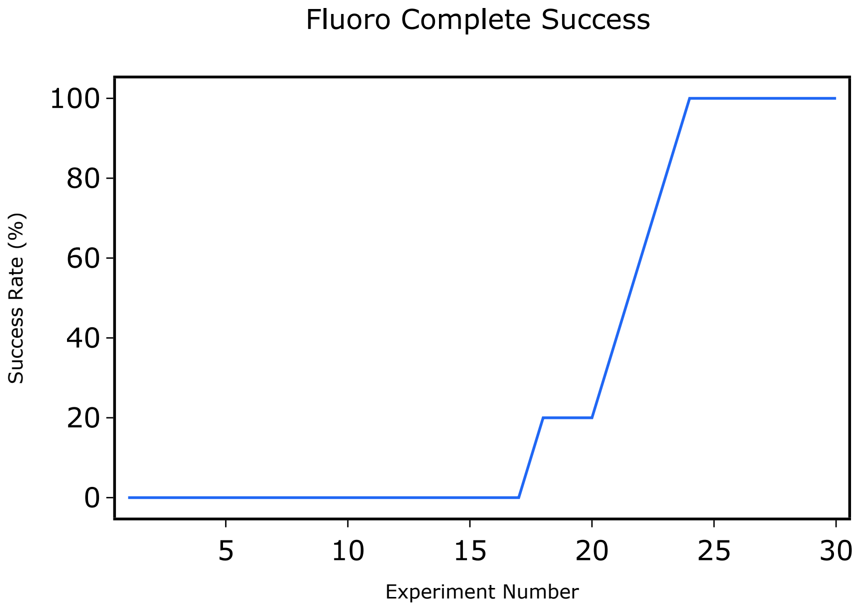

Figure S92: Ciprofloxacin intermediate digital twin case study 8 success rate plot

Case study 9 result figures

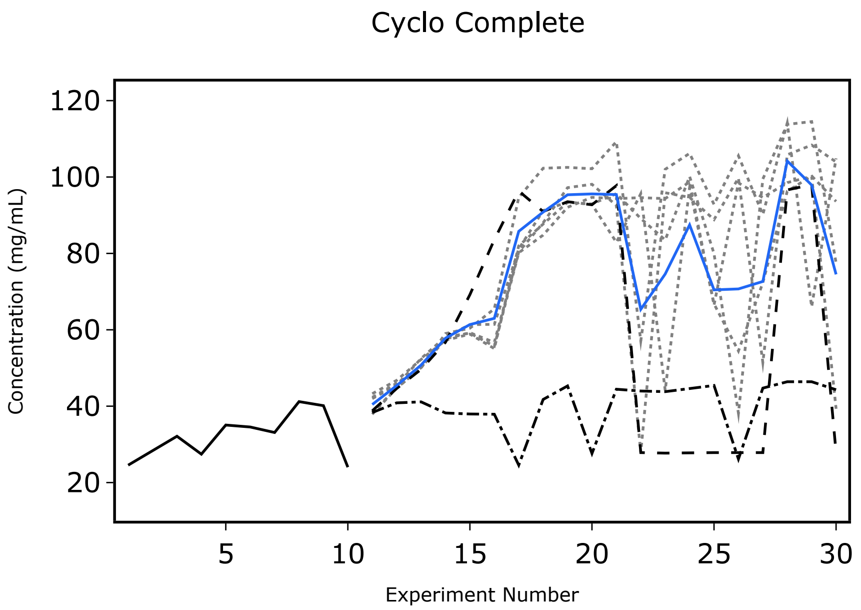

Figure S93: Ciprofloxacin intermediate digital twin case study 9 raw data

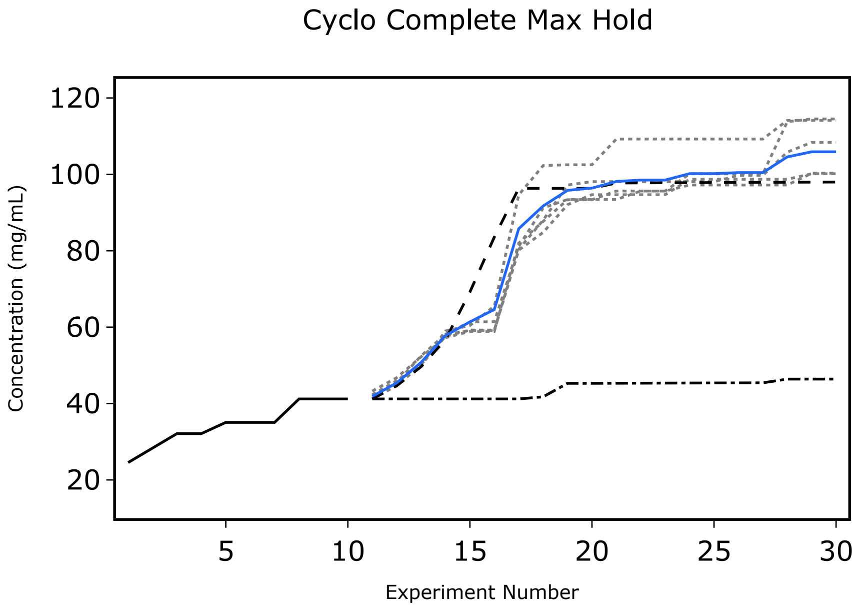

Figure S94: Ciprofloxacin intermediate digital twin case study 9 max-hold transform of raw data

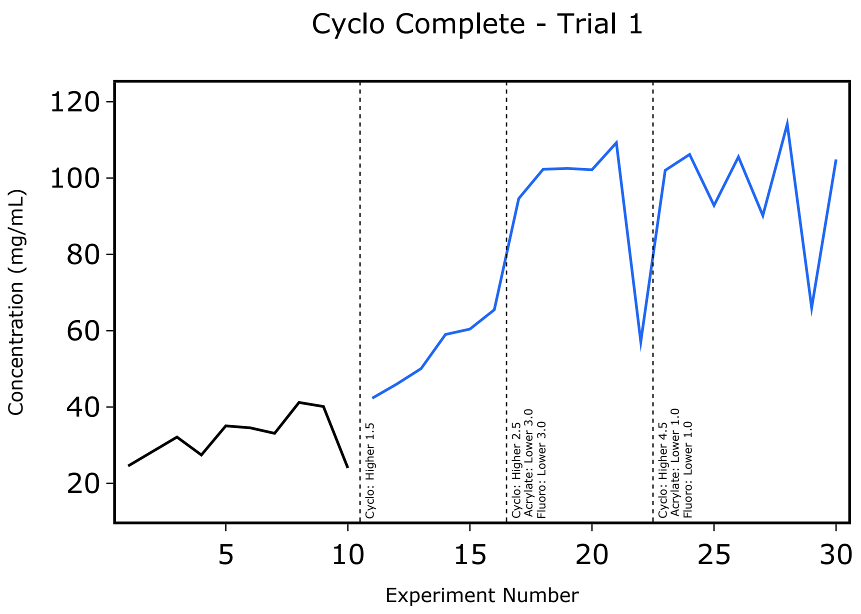

Figure S95: Ciprofloxacin intermediate digital twin case study 9 trial 1 raw data with expansion annotations

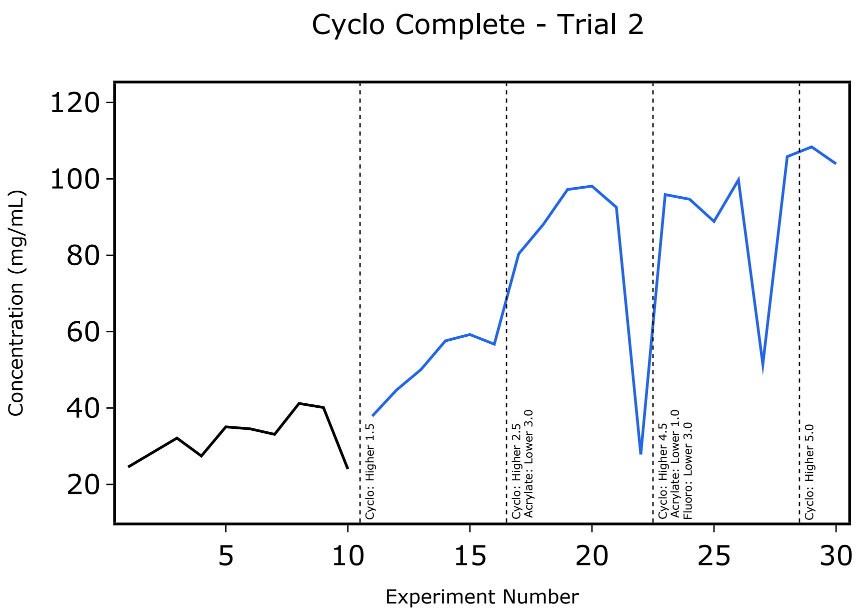

Figure S96: Ciprofloxacin intermediate digital twin case study 9 trial 2 raw data with expansion annotations

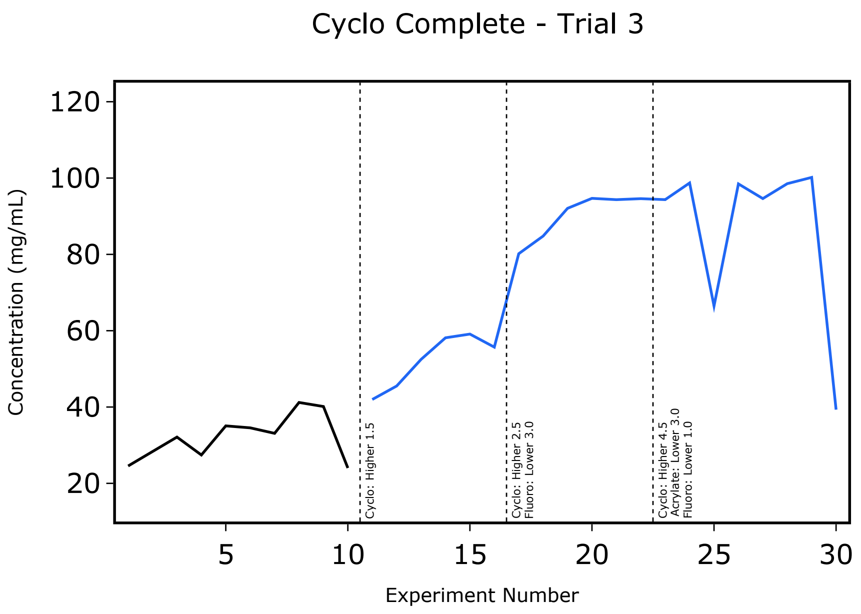

Figure S97: Ciprofloxacin intermediate digital twin case study 9 trial 3 raw data with expansion annotations

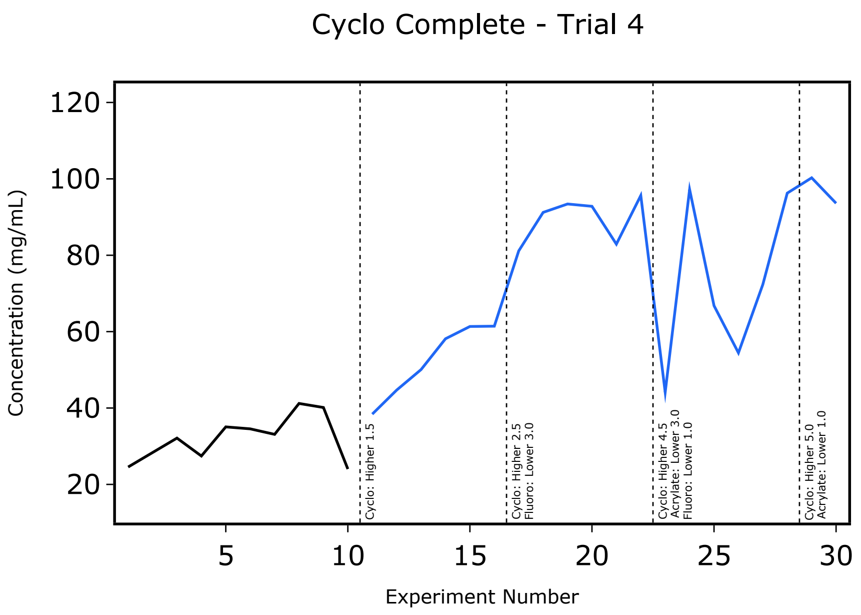

Figure S98: Ciprofloxacin intermediate digital twin case study 9 trial 4 raw data with expansion annotations

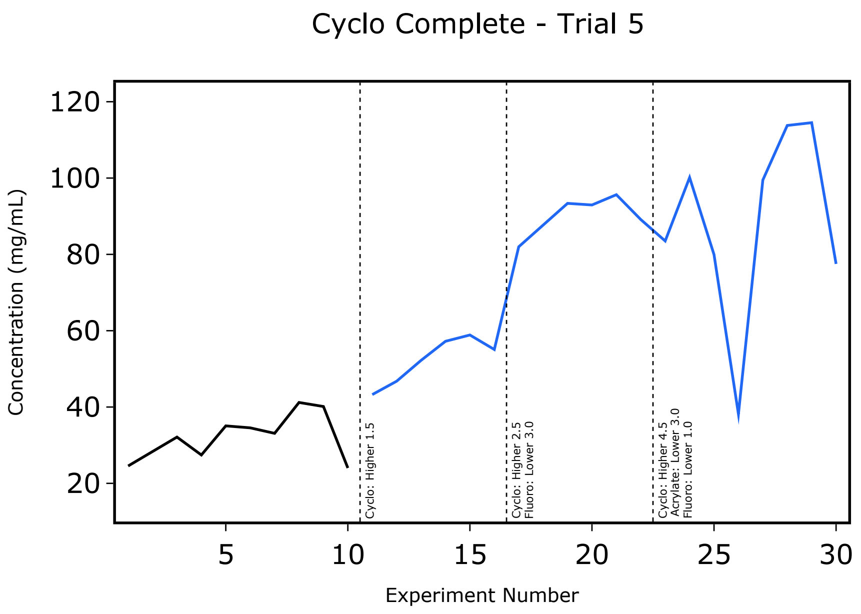

Figure S99: Ciprofloxacin intermediate digital twin case study 9 trial 5 raw data with expansion annotations

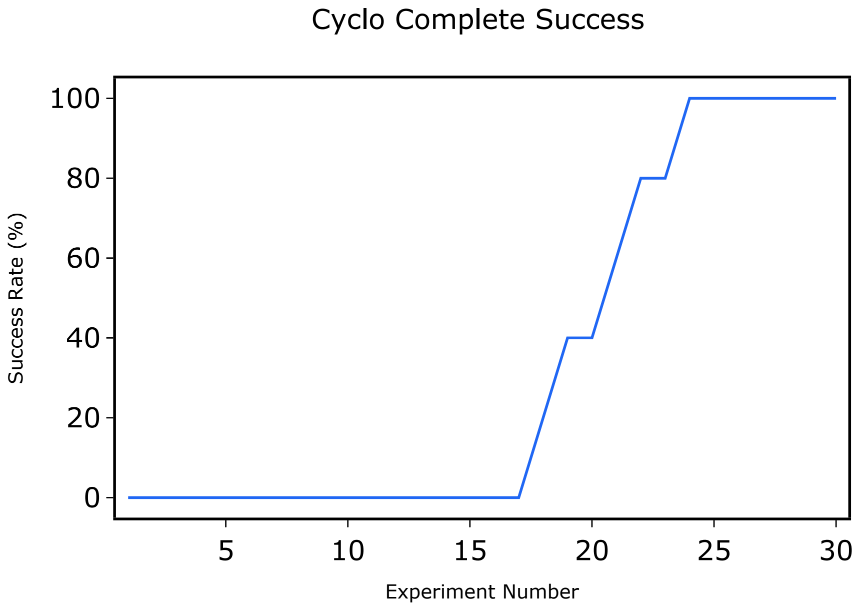

Figure S100: Ciprofloxacin intermediate digital twin case study 9 success rate plot

Safety statement

There were no unexpected or unusually high safety hazards encountered during the experimentation.

References

(1) Felton, K. C.; Rittig, J. G.; Lapkin, A. A. Summit: Benchmarking Machine Learning Methods for Reaction Optimisation. *Chemistry–Methods* **2021**, *1* (2), 116–122. https://doi.org/10.1002/cmtd.202000051.
